# Storing redox equivalent in the phenalenyl backbone towards catalytic multi-electron reduction†
†Electronic supplementary information (ESI) available: Detailed experimental procedures, spectra (NMR, and HRMS), crystallographic details, and the coordinates of the computed structures. CCDC 1828004 and 1846773. For ESI and crystallographic data in CIF or other electronic format see DOI: 10.1039/c9sc02057h


**DOI:** 10.1039/c9sc02057h

**Published:** 2019-06-10

**Authors:** Mrinal Bhunia, Sumeet Ranjan Sahoo, Bikash Kumar Shaw, Shefali Vaidya, Anand Pariyar, Gonela Vijaykumar, Debashis Adhikari, Swadhin K. Mandal

**Affiliations:** a Department of Chemical Sciences , Indian Institute of Science Education and Research-Kolkata , Mohanpur-741246 , India . Email: swadhin.mandal@iiserkol.ac.in; b Department of Chemical Sciences , Indian Institute of Science Education and Research-Mohali , SAS Nagar-140306 , India . Email: adhikari@iisermohali.ac.in

## Abstract

Storing and transferring electrons for multi-electron reduction processes are considered to be the key steps in various important chemical and biological transformations. In this work, we accomplished multi-electron reduction of a carboxylic acid *via* a hydrosilylation pathway where a redox-active phenalenyl backbone in Co(PLY-O,O)_2_(THF)_2_, stores electrons and plays a preponderant role in the entire process. This reduction proceeds by single electron transfer (SET) from the mono-reduced ligand backbone leading to the cleavage of the Si–H bond. Several important intermediates along the catalytic reduction reaction have been isolated and well characterized to prove that the redox equivalent is stored in the form of a C–H bond in the PLY backbone *via* a ligand dearomatization process. The ligand's extensive participation in storing a hydride equivalent has been conclusively elucidated *via* a deuterium labelling experiment. This is a rare example where the ligand orchestrates the multielectron reduction process leaving only the metal to maintain the conformational requirements and fine tunes the electronics of the catalyst.

## Abstract 2

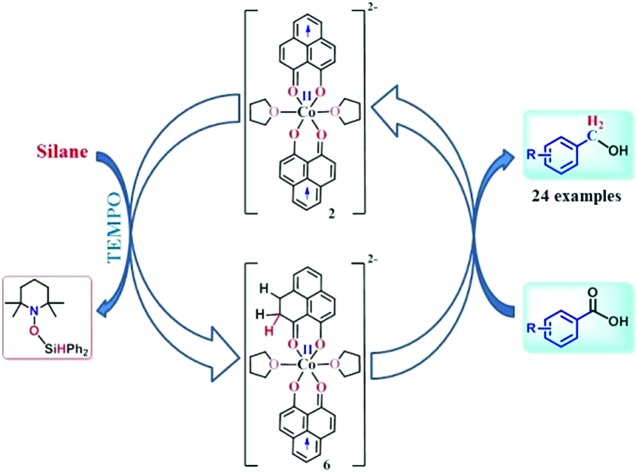
Redox equivalent storage in the phenalenyl backbone towards catalytic multi-electron reduction.

## Introduction

Multi-electron reduction processes are ubiquitous in nature and are involved in extremely important chemical conversions such as nitrogen to ammonia or carbon dioxide to methanol.^1^ In this context, two major emerging chemical strategies for multi-electron reduction are either to use a multi-metallic cluster^2^ or to coordinate a metal ion by an appropriate redox active ligand,^3^ which actively shares its electrons with the frontier orbitals of the metal for efficient functioning. However, the redox active ligand rarely becomes amenable to conducting chemical events like direct bond formation or to function as a key player to handle this challenging problem of multielectron reduction. The catalytic multielectron reduction of carbonyl groups in carboxylic acids and other acid derivatives is one of the very fundamental techniques in organic transformations.^4^–^11^ Current burgeoning interest in carboxylic acid reduction further relies on the product fatty alcohols and related value-added chemicals starting from fatty acids. Historically, small to medium scale reduction of carboxylic acids utilizes stoichiometric reductants such as sodium borohydride, lithium aluminum hydride *etc.*^12^–^17^ The increased attention to atom economy and greener methods make the use of such stoichiometric reagents unattractive. On the other hand, the use of hydrogen in the presence of transition metal complexes may be very atom efficient,^18^–^23^ yet the flammability and required high pressure of the gas are the major disadvantages in such reduction. On the contrary, catalytic reduction by silanes under mild conditions may be an appealing alternative that circumvents the use of high pressure equipment. Furthermore, the use of silanes as a reductant may offer fine tuning of their reducibility by the substituents on the silicon atom and will likely be used often in the future for challenging organic synthesis. It may be worth noting that carboxylic acid derivatives are often inert toward reduction and except two reports^24^,^25^ which used either high temperature or UV light irradiation, catalytic hydrosilylation of carboxylic acids using base metal catalyst under ambient conditions has scarcely been reported.

**Scheme 1:**
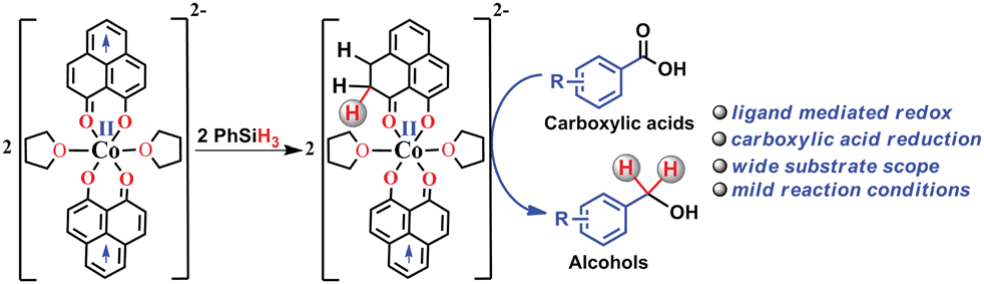
Overview of the present work where the multielectron redox process is predominantly steered by the phenalenyl (PLY) ligand motif.

Herein, we report an efficient cobalt complex which catalyzes the reduction of carboxylic acid under mild conditions. The complex comprises a redox active ligand phenalenyl (PLY), which stores the redox equivalent in a C–H bond *via* ligand dearomatization and promotes a radical catalyzed reduction of carboxylic acid (Scheme 1). Over the last few decades, an ample number of phenalenyl based systems have been developed by Haddon,^26^–^30^ Nakasuji,^31^ Takui,^32^–^34^ Kubo,^35^ Morita,^36^,^37^ and co-workers which heralded a new era in designing organic molecular conductors, molecular batteries,^38^ quantum spin simulators^39^ and spin electronic devices.^40^ We have a long-standing research interest in expanding the applicability of PLY as a redox storage motif and using such a redox delocalized ligand to perform a wide variety of challenging organic transformations in a catalytic fashion.^41^–^45^ During this investigation, we for the first time disclose that a PLY backbone can store electrons *via* a dearomatization process with concomitant formation of an sp^3^ hybridized C–H bond, followed by catalytic hydrogen transfer, to achieve multi-electron reduction. This example unravels such a predominant role of the PLY backbone to steer the multielectron reduction which almost expatriates only the metal to take care of the conformational rigidity of the molecule and fine tune the electronics. Storing the redox equivalent *via* ligand dearomatization also to an extent mimics the way NAD(P)H works in natural systems.

## Results and discussion

**Fig. 1:**
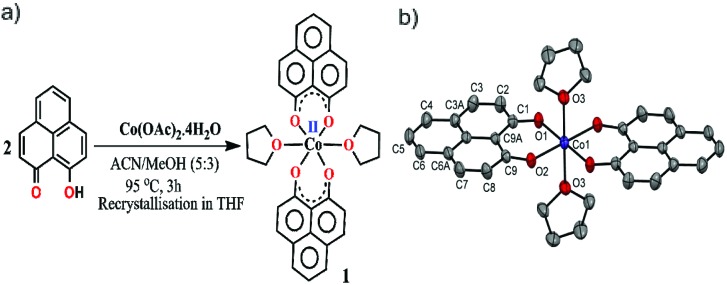
(a) Synthetic scheme for cobalt complex **1**. (b) A perspective ORTEP view of the molecular structure (50% ellipsoid level) of **1**, where hydrogen atoms are omitted for clarity.

The redox active backbone that has been used in this study is 9-hydroxyphenalenone, a subclass of phenalenyl (PLY). The (PLY-O,O)_2_Co(THF)_2_ complex, **1** was prepared by drop-wise addition of methanolic solution of Co(OAc)_2_·4H_2_O to a hot acetonitrile solution of 9-hydroxyphenalenone and refluxing the reaction mixture at 95 °C for 3 h to give a crystalline precipitate (Fig. 1a). Analytically pure deep red colored crystals of **1** were obtained by recrystallizing the reaction product from dry THF at 5 °C in nearly 68% yield. Complex **1** is expectedly paramagnetic and was characterized using an array of analytical tools including elemental analysis, ESI-MS, IR spectroscopy and single crystal X-ray diffraction studies. The atomic connectivity in **1** was firmly established by the single crystal X-ray diffraction studies and it demonstrates an octahedral ligand environment for the six-coordinate cobalt(ii) ion. The ORTEP diagram (Fig. 1b) displays that Co(ii) ion is coordinated to four O-donor atoms of two phenalenone ligands (O1 and O2 atoms as coordination site) and the axial positions are occupied by two weakly bound THF ligands (Co–O3 = 2.210 (2) Å).

**Fig. 2:**
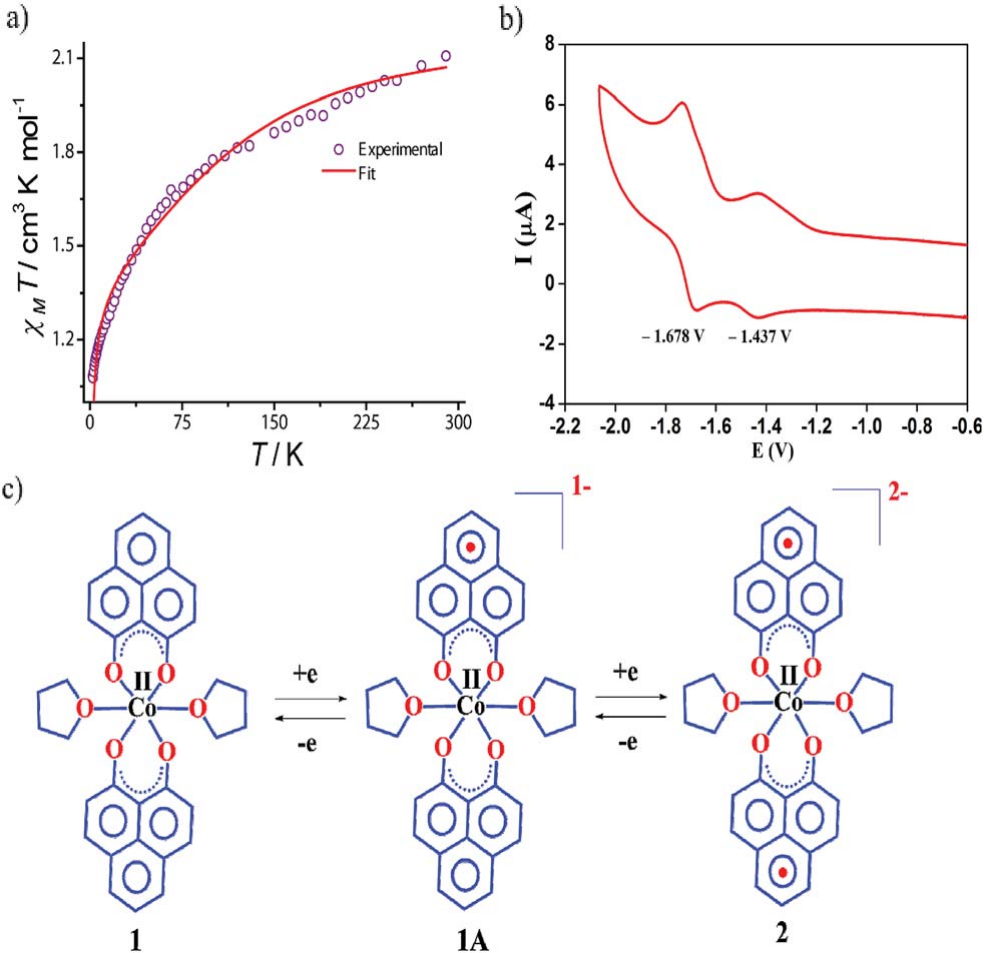
(a) The variable temperature dc magnetic susceptibility data of **1** under 1000 Oe applied dc field. (b) Electrochemical behavior of the complex **1**. CV of **1** in DMSO at a scan rate of 0.1 V s^–1^ was acquired using a Pt working electrode, Pt-wire as the counter and reference electrode, internally referenced to ferrocene (Fc)/ferrocenium (Fc^+^). (c) Two successive one electron reductions showing the formation of phenalenyl-centered radical anions, without affecting the metal's oxidation state.

The dc magnetic susceptibility measured on a polycrystalline sample of **1** discloses a *χ*_m_*T* value of 2.10 cm^3^ mol^–1^ K at 290 K, which gradually decreases to 1.08 cm^3^ mol^–1^ K at 2 K. The room temperature susceptibility is higher than the spin only value (expected 1.875 cm^3^ mol^–1^ K for a high spin Co(ii) assuming *g* = 2) and originates from the significant orbital angular momentum contribution. This is fully anticipated, since the ground term of the Co(ii) is ^4^T_1_, which invokes first-order spin–orbit coupling. A significant anisotropy is present in the system which causes the susceptibility value to drop sharply over lowering the temperature and simulation of experimental data points discloses the zero-field splitting to be *D* = –116 cm^–1^ (Fig. 2a, red line). The electrochemical properties of the previously synthesized transition metal complexes on the same ligand motif revealed that the reductions are primarily ligand based, given the low-energy redox orbitals present on the highly delocalized ligand framework.^41^ To assess the reduction potentials for **1**, we conducted a cyclic voltammetry experiment, which revealed two successive one-electron reversible waves at –1.44 and –1.68 V (*vs.* Fc/Fc^+^ couple, Fig. 2b). The close proximity of these reduction potential values to those of previously reported (PLY-O,O)_2_Ni(THF)_2_ strongly suggests the reductions are primarily ligand based.^43^ The Δ*E*^2–1^ value from the voltammogram is –0.24 V, which is also in close agreement to bis-phenalenyl–boron complexes bearing the same phenalenyl based ligand (the Δ*E*^2–1^ values are –0.29 and –0.28 V). The conformity in the Δ*E*^2–1^ values provides additional support for successive two step one-electron reductions into the phenalenyl moiety (Fig. 2c).^29^

**Fig. 3:**
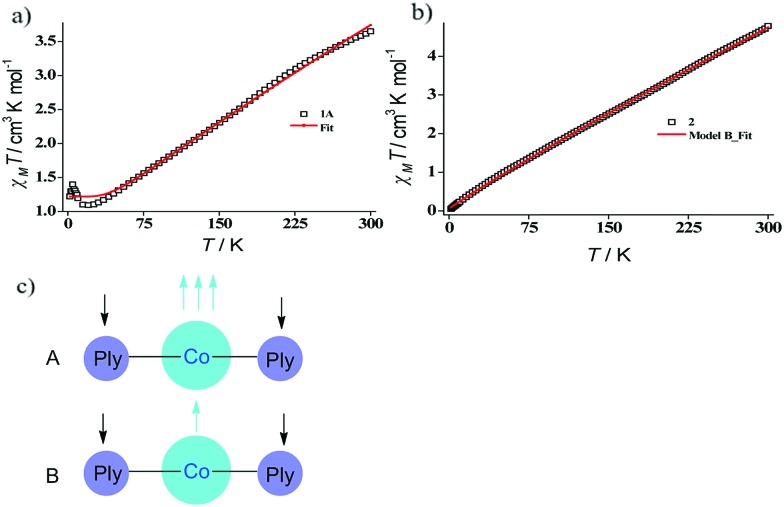
(a) Solid state magnetic susceptibility data for **1A** and the theoretical fit. (b) Solid state magnetic susceptibility data for **2** and the theoretical fit. (c) Two possibilities (models A and B) of reaching overall *S* = 1/2 state for a ML_2_ system such as **2**, where each PLY ligand is mono-reduced. Model B invokes a spin crossover on the Co(ii) center to possess a metal-centric single unpaired electron.

Indeed, we were able to perform the sequential two one-electron reductions chemically and isolated both products to prove that the major locus of the injected electrons is the ligand backbone by studying their detailed magneto-chemical properties (*vide infra*). The chemical reduction of **1** with one equivalent of potassium was carried out in THF and a yellow-green colored solid, **1A** was isolated in good yield (94%). The magnetic susceptibility data for **1A** displays a *χ*_m_*T* value of 3.70 cm^3^ mol^–1^ K at room temperature which also monotonically decreases with lowering temperature (Fig. 3a). The antiferromagnetic coupling between a ligand-centered electron and the metal electron with decreasing temperature, becomes apparent and at a low temperature regime, it describes overall a *S* = 1 ground state. To estimate the magnitude of AF-coupling between metal and ligand electrons, experimental data were fitted with the following Hamiltonian,

1 *H* = –2*JS*_*i*_*S*_*j*_

where *S*_*i*_ is the cobalt-based spin and *S*_*j*_ is the PLY-based spin. The best fit line provides the following parameters; *g* ∼ 2.22, *J* = –46.11 (2) cm^–1^, *N*_α_ = 9.1 × 10^–3^ emu mol^–1^ (TIP), and *ρ* = 4.7% (*ρ* is the amount of paramagnetic impurity).^46^ Pleasingly, the simulated *g* value is in good agreement with experimentally observed *g* values for other anisotropic Co(ii) systems reported previously.^47^ Next, we attempted to reduce **1** by two electrons since we posited that this bis-ligated complex can perform two electron redox processes as suggested by the CV data (*vide supra*). Accordingly, the use of two equivalents of the reductant in THF evinces an immediate color change from maroon to green, which resulted in 92% yield of a solid powder of **2**. The two-electron reduced product **2** is sensitive to both moisture and air but can be stored for weeks under an inert atmosphere. Multiple attempts toward crystallizing **2** failed, likely due to the sensitive nature of the species in solution state for a prolonged time. Our further attempt to trap the potassium by adding a crown ether also did not help the crystallization process. Nevertheless, the product was characterized by an array of UV-vis, IR spectroscopy, magnetochemistry, and theoretical calculations. To understand the electronic structure of **2**, the solid sample was subjected to variable temperature magnetic susceptibility measurement. As expected, the room temperature *χ*_m_*T* value of 5.40 cm^3^ mol^–1^ K supports altogether a non-interacting Co(ii) (*S* = 3/2) metal ion and two radicals on the ligand backbone (Fig. 3b).

The susceptibility value drops precipitously with lowering temperature and at 4 K, the value becomes vanishingly small. This is only probable, when two *S* = 1/2 molecules can interact in a strong AF-coupled manner, since no other coupling mechanism can generate a diamagnetic ground state starting with a metal center of *S* = 3/2 and two ligand-based radicals. Logically, two probable spin-coupling scenarios can be conceived to explain the observed ground state for the coupled system (Fig. 3c). Model A: three cobalt electrons (three α, from high spin Co(ii)) are AF-coupled to two ligand-based β electrons. Model B: one cobalt electron (one α, from low spin Co(ii) upon spin crossover (SCO)) is AF-coupled with two ligand β electrons. To clearly discern these possibilities, we theoretically fitted the data and observed that the nature of fitting improved significantly if the second probability (model B) is traced. The Hamiltonian used for the fitting is as follows:

2 *H* = –2*J*(*S*_*i*_*S*_*j*_ + *S*_*i*_*S*_*k*_)

3
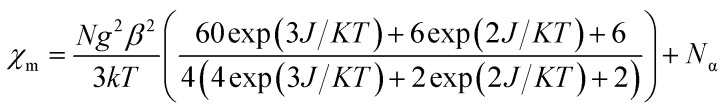
where *S*_*i*_ is the spin for low spin cobalt(ii) (*S*_*i*_ = 1/2), *S*_*j*_ and *S*_*k*_ are ligand-based spins and AF-coupled to *S*_*i*_ = 1/2 on cobalt at equal magnitude. The obtained parameters from the best fit line are *g* ∼ 2.21, *J* = –44.65 (2) cm^–1^ (*θ* = +4.52 cm^–1^), *N*_α_ = 9.1 × 10^–3^ cm^3^ mol^–1^, and *ρ* = 1.2%. The excellent theoretical fit (*R*^2^ = 1.5 × 10^–4^) indicates that a spin cross over (SCO) very likely leads to low-spin cobalt(ii) starting from its high-spin geometry. The fit of the experimental *χ*_m_*T* invoking model A is poor and has been presented in the ESI (Fig. S3†). To substantiate our assertion regarding SCO, we performed a temperature-dependent hysteresis experiment on **2** and found a sizable gap (∼25 K) between heating–cooling traces (Fig. S4, ESI†). The observation of the hysteresis conclusively proves that the Co(ii) center in **2** is undergoing a SCO at approximately 150 K and the electronic structure depicted in model B is a valid description of its ground state.^48^ The phenomenon of SCO in a pseudo-octahedral Co(ii) system is also supported by Chirik's report of SCO in a distorted square planar (APDI)CoCl (APDI = 2,6(RN = CMe)_2_C_5_H_3_N) system at a low temperature regime.^49^ Furthermore, computationally we evaluate that the electronic structure with *S* = 1/2 ground state having low-spin cobalt is significantly more stable than the alternative possibility (*vide infra*). Supposedly, the strong intermolecular AF-coupling between two *S* = 1/2 centers led to near diamagnetism at 4 K which is completely plausible since phenalenyl molecules are known to interact strongly *via* multi-center pancake π-dimer formation.^35^,^50^,^51^ Such a strong intermolecular AF-interaction is likely facilitated by the flat nature of the PLY-ring with an open-shell radical delocalized over the singly occupied molecular orbital (SOMO). Overall, the donated electrons by an external reductant (both in one and two-electron reduction cases) reside on the PLY-ligand backbone and the oxidation state of the cobalt remains unaltered during reduction (Fig. 3a and b). This fact is fully consistent with a redox active PLY-ligand where the highly conjugated ligand backbone harbors the redox equivalent. We have previously observed that the redox active PLY can facilitate single electron transfer (SET) to a class of substrates.^42^–^44^ In recent reports, we established that the reduced phenalenyl radical triggers a SET process to execute transition metal-free C–H functionalization.^42^,^44^ Since redox equivalents can be stored in **2**, we envisaged that similar SET could be augmented to accomplish reduction of carboxylic acids by silane.

Accordingly, we conducted carboxylic acid reduction using silanes, catalyzed by **1** which underwent an *in situ* reduction by potassium. As a representative example, benzoic acid was reduced through the hydrosilylation pathway when two equivalents of PhSiH_3_ were treated in the presence of 5 mol% of complex **1** and 10 mol% of potassium in THF under anaerobic atmosphere. Gratifyingly, a quantitative conversion of the carboxylic acid to benzyl alcohol was realized after 24 h at rt (Table S1, entry 5 as the optimized condition, ESI†) without formation of any other byproduct. Multiple control reactions were conducted to ascertain the necessity of both catalyst **1** and the reductant potassium. The redox active PLY plays a definitive role in the reduction process as interrogated by using a Co(acac)_2_ (acac = acetylacetonate) complex where cobalt maintains similar geometry when coordinated with the THF,^52^ provided a drastically reduced yield (5%) of the corresponding alcohol. To investigate the suitability of other reductants in preparing the catalytically relevant species **2***in situ*, we used both sodium and an organic amine tetrakis(dimethylamino)ethylene (TDAE). Both cases gave very poor yields prompting us to conclude that a strong reductant is necessary to deliver the redox equivalents under moderately large negative potential for successful catalytic reduction of carboxylic acids. Notably, as sodium is sufficiently reducing from a thermodynamic viewpoint, we assume the failure of reducing **1** with sodium originates from kinetic inhibition. To prove this conjecture, the solution of **1** with sodium was sonicated and the resulting material was used for catalysis. A significantly improved yield (34%) as compared to the yield without sonication (11%) of benzyl alcohol supports our assumption that the failure of sodium as a reductant does not stem from a thermodynamic background. Having the optimized reaction conditions in hand, we further explored the substrate scope of various carboxylic acids in the hydrosilylation reaction (Table 1). Under standardized conditions, benzoic acid, 4-methylbenzoic acid and 4-methoxybenzoic acid afforded an excellent isolated yield of the corresponding benzyl alcohols **4a**, **4b** and **4c**, respectively. Benzoic acids containing an electron withdrawing group at the 4-position such as chloro, bromo, iodo and trifluoromethyl also offered good to excellent yields of **4d–4g** (52–91%). Interestingly, we found that our reductive protocol is considerably chemoselective and spares the reduction of highly reducible groups such as 4-nitro and 4-cyano present in the substrate (entries **4h** and **4i**). When the carbonyl group is present along with carboxylic acid, chemoselective reduction of carboxylic acid is still achieved to a fair extent along with minor product from simultaneous reductions. For example, when 4-formylbenzoic acid was treated under optimized reaction conditions, 82% of 4-formylbenzyl alcohol (**4j**) was observed along with 18% of 1,4-phenylenedimethanol. Next, we inquired whether the position of the substituents in carboxylic acids has any influence on the outcome of the reduction. Accordingly, 3-methyl, 3-chloro and 3-bromobenzoic acids were treated under analogous reaction conditions and the respective reduced products **4l**, **4m** and **4n** were isolated in very good yields. Significantly, *ortho*-substituted benzoic acids such as *o*-methoxy, *o*-chloro, *o*-bromo and *o*-iodobenzoic acid were well tolerated with this hydrosilylation method and afforded very good to excellent yield (69–84%) of the reduced products **4o–4r** irrespective of their posing steric encumbrance. However, the presence of a more sterically demanding group such as 2,4,6-trimethyl, reduces the yield of the corresponding benzyl alcohol **4u** to 52%. Under standard conditions, 4-chloro-3-nitrobenzoic acid and 2-chloro-5-nitrobenzoic acid also rendered good yields to the corresponding benzyl alcohols **4w–4x** (63% and 71%, respectively). Essentially, **1** is a precatalyst which is reduced *in situ* by an appropriate reductant, and the reduced ligand backbone advances the SET. To ensure that potassium cation has no special role in catalysis and rather behaves as a countercation, we performed an exchange reaction with a sodium cation resulting in similar activity (86% yield of benzyl alcohol). This fact indicates the role of potassium as a charge balancing countercation.

**Table 1:** Scope of cobalt-catalyzed reduction of carboxylic acids^*a*^

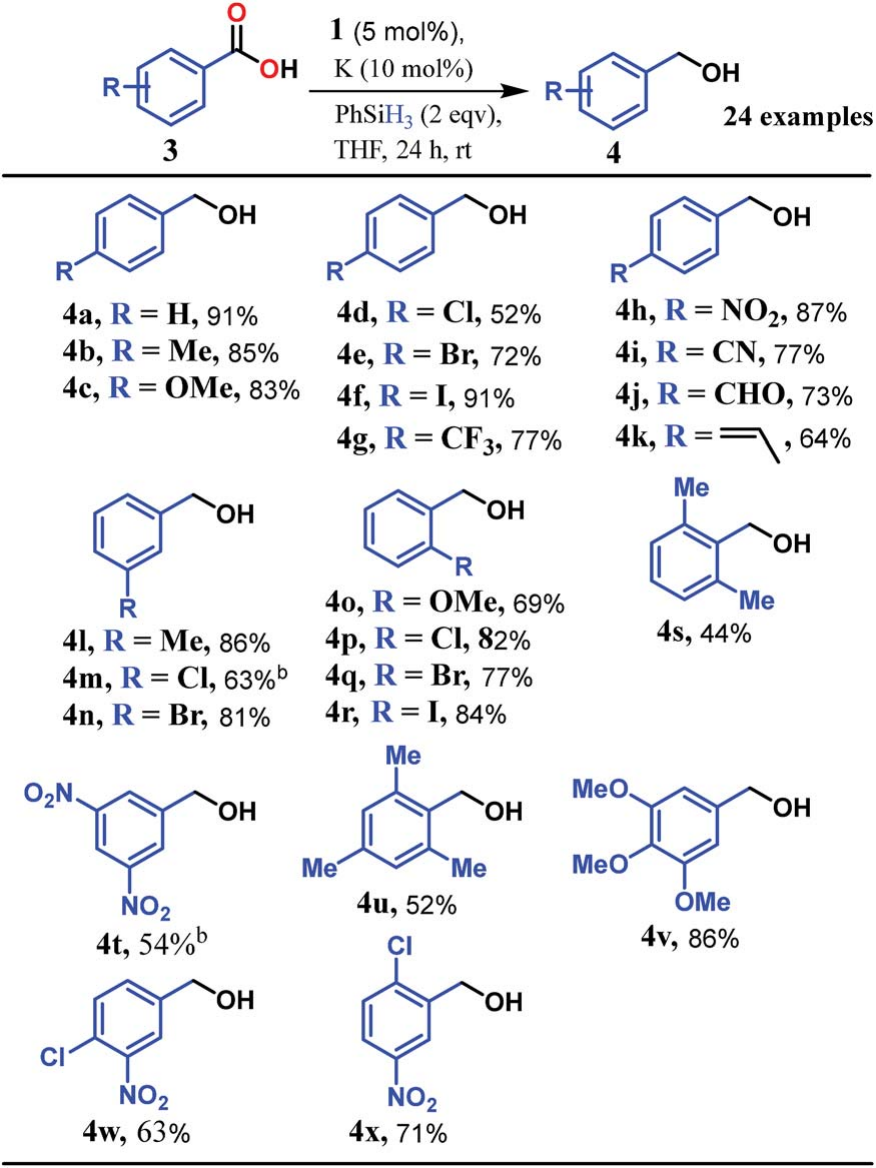

^*a*^Reaction conditions: catalyst **1** (5.0 mol%), K (10.0 mol%), **3** (0.5 mmol), PhSiH_3_ (1.0 mmol, 2.0 equiv.), THF (1.0 mL), room temperature, 24 h. Hydrolysis was performed with 2.0 (M) NaOH solution.

^*b*^The yields were determined from ^1^H NMR spectroscopy using *m*-xylene as an internal standard.

**Fig. 4:**
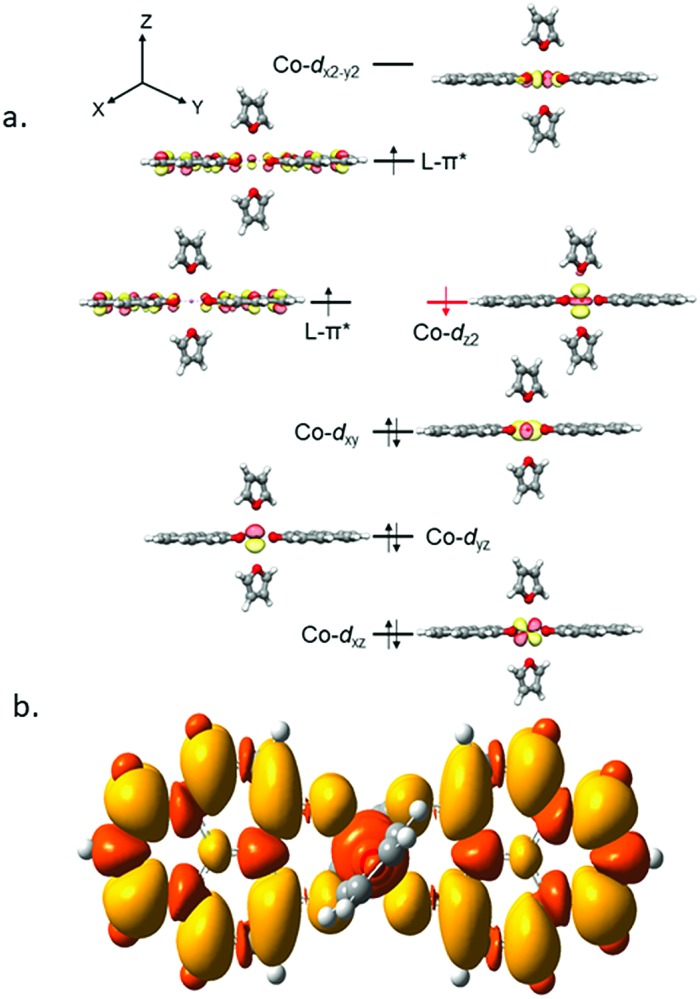
(a) The MO picture of **2** at the ground state, where low spin Co(ii) is coordinated to two PLY ligand backbones retaining α spins. (b) The spin density plot for the ground state of **2**. The yellow and orange colors designate excess α and excess β spins, respectively. The contour values are set to ±0.0004 (e bohr^–3^)^1/2^.

It is imperative from the above study that the electronic structure of **1** comprising a redox active backbone is the key to governing the success of this catalytic process. To further investigate the electronic structure of the molecule, we undertook density functional theory (DFT, B3LYP/Lanl2DZ(6-31+G*)) analysis. The structure of **1** reveals good covalency in the Co–O bonds as significant spin density (0.18 electron each) is observed in all four oxygens in the plane of PLY. The frontier molecular orbital analysis of the molecule discloses that the LUMO of **1** is based on a PLY-ligand (Fig. S5, ESI†). Hence, it is anticipated that the first reduction will likely be ligand based without changing the oxidation state of the cobalt center. In strong agreement, the SQUID magnetometry data for the mono-reduced product **1A** reveals a total of four uncoupled electrons at room temperature and significant AF-coupling between the metal and ligand-based electrons at low temperature (*vide supra*). Computationally, the ground state of **1A** is a broken symmetry triplet, which is electronically stable by 5.46 kcal mol^–1^ over the uncoupled quintet state (uncorrelated *S* = 3/2 from Co(ii) and *S* = 1/2 from one ligand). The frontier orbitals of **1A** reveal that the LUMO is again primarily ligand based (Fig. S5, ESI†), justifying that the second reduction happens further in the ligand backbone to generate two-electron reduced species **2**, in confirmation with experimental findings. The ground electronic structure of **2** shows that the broken symmetry solution for the *S* = 1/2 state (where low-spin Co(ii) is coupled with two ligand electrons with opposite spin) is significantly lower (electronically ∼15 kcal mol^–1^) than that of the state comprising five electrons altogether. This is in conformity with our solid state magnetic moment measurement where we do observe weak AF-coupling between the metal and ligand electrons with a probable high to low spin conversion of the Co(ii) center (*vide supra*). This electronic state is also significantly lower in energy than the electronic structure considered as model A in the magneto-chemistry section (Fig. 3c). We note that a thorough multiconfiguration treatment will offer a more reliable energy gap between different spin states. As inferred from the natural population analysis on broken symmetry solution of **2**, the 3d-electron occupation for cobalt is 7.5, which justifies the retention of the cobalt oxidation state to +2 and precludes any strictly metal-based reduction. As shown in Fig. 4a, the molecular orbital picture of **2** depicts that the lone cobalt electron is housed in a d_z_^2^ orbital, and the other two spin bearing centers are ligand-based orbitals. The spin density plot for the electronic structure vividly depicts that two ligands are housing two alpha spins where the cobalt contains one beta spin (Fig. 4b). The <*S*^2^> value for the optimized structure is 0.91, which suggests this is a bona fide broken symmetry solution of the electronic structure of **2** (expected uncontaminated <*S*^2^> is 0.75). Overall, the computationally evaluated electronic structures of mono-reduced species **1** and catalytically active **2** corroborate nicely with detailed magneto-chemical analyses performed (*vide supra*).

**Fig. 5:**
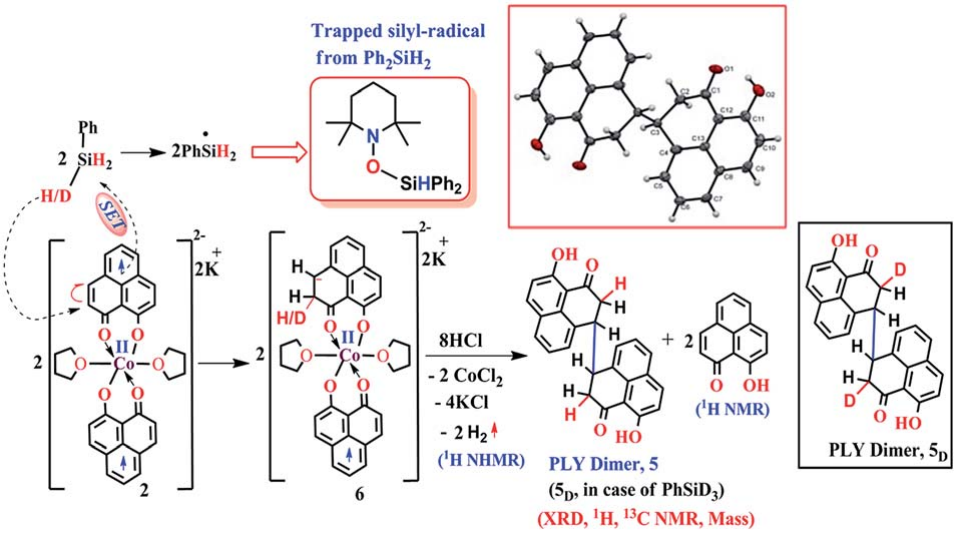
Plausible stepwise mechanism for the formation of the σ-dimer, **5** and its ORTEP diagram (50% ellipsoid level).

The successful catalytic reduction of carboxylic acid, which is greatly supported by the redox active delocalized ligand backbone, inspired us to study the mechanistic course of the reaction thoroughly. Our previous finding that the PLY backbone behaves as a redox reservoir and triggers a radical catalyzed reaction, prompted us to examine whether this reduction also adopts a radical pathway.^43^ In agreement with this hypothesis, we observed that the addition of a radical quencher TEMPO in two equivalents completely shuts down the reaction. We propose that **2** initiates a SET process activating the Si–H bond of silane *via* formation of a hydride and silyl radical. To examine the formation of a silyl radical, we added TEMPO to **2** in the presence of a silane (Ph_2_SiH_2_) to isolate the TEMPO trapped silyl radical intermediate and it was characterized by mass spectrometry (see ESI†). We further reveal, rather surprisingly, that **2** stores the redox equivalents on the PLY framework as a newly formed C–H bond. To substantiate our claim, the reaction mixture of **2** with PhSiH_3_ (in the absence of carboxylic acid substrate) was treated with aq. HCl to leach the metal ion from the organic ligand part. The ^1^H NMR spectroscopy of the separated organic product reveals a peak at *δ* 12.71 ppm (O–H of a new phenalenone) in corroboration with the formation of a PLY-dimer (**5**) which has been characterized further crystallographically (Fig. 5, inset). The ^1^H NMR analysis of the isolated organic product reveals aliphatic peaks at *δ* ∼ 3.65 and 3.15 ppm with a relative intensity of 1 : 2, suggestive of the dearomatization process taking place in the PLY ring. The formation of such a σ-dimer may be attributed to the dimerization between two PLY based radicals whose plausible formation has been delineated in Fig. 5. The addition of hydride to one of the PLY backbone leads to dearomatization and formation of **6**. Addition of HCl promotes transfer of a single electron to H^+^ from intermediate **6** generating hydrogen atoms, which readily recombine to produce H_2_ gas, whose formation was unambiguously authenticated by a ^1^H NMR resonance at *δ* 4.56 ppm.

**Scheme 2:**
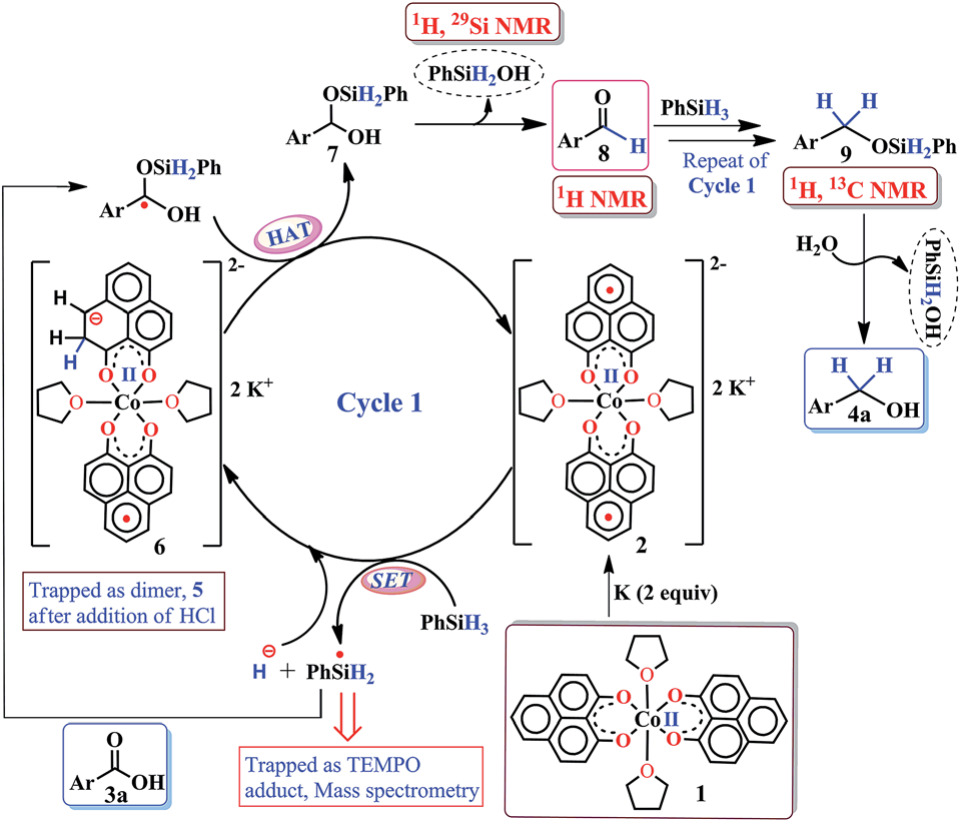
A plausible mechanistic course for the catalytic carboxylic acid reduction by **2**.

Upon further scrutiny of the X-ray structure for **5**, it was revealed that the newly formed C–C bond is elongated (C3–C3′ = 1.571(8) Å), which corroborates well with the varying length of long C–C bonds (1.60–1.65 Å) earlier reported by Haddon and Kubo in similar σ-dimers.^50^,^53^ The non-aromatic puckered ring attains envelope type architecture and the CH_2_ carbon is placed 0.48 Å above the mean plane formed by the other two rings. All NMR spectroscopic experiments along with X-ray crystallographic analysis strongly suggest that a hydride is transferred to the ligand backbone resulting in the dearomatization of the PLY backbone. To gather unambiguous proof that the ligand backbone stores hydride in the form of a C–H bond, we additionally performed a deuterium labelling experiment. Treatment of **2** with a stoichiometric amount of PhSiD_3_ and further quenching the reaction mixture clearly furnishes the isotopomer of **5**, **5**_D_ (Fig. 5, inset). The formation of **5_D_** is authenticated by mass spectrometry and NMR spectroscopy, exhibiting a resonance at *δ* 3.33 ppm in the ^2^H NMR spectrum. In the ^13^C NMR spectrum of **5_D_**, the carbon attached to deuterium shows up at *δ* 27.5 ppm as a prototypical 1 : 1 : 1 triplet (^1^*J*_^13^C–D_ = 20 Hz) owing to deuterium coupling. Moreover, the deuterium isotopologue of the final product, PhCH(D)OH, **4_D_** was formed in 83%, when the substrate was reduced with PhSiD_3_ in dry THF-d_8_ solvent (see ESI, Fig. S74†). The nature of **4_D_** was also authenticated by a battery of ^2^H, ^1^H, ^13^C NMR spectroscopies and mass spectrometry. The incorporation of deuterium in the ligand backbone as well as its presence in the product (alcohol) unambiguously establishes the proposed mechanistic steps (Scheme 2). To further ensure that the source of the hydride trapped on the ligand backbone does not originate from an adventitious proton source, **2** was treated with aq. HCl in the absence of any silane resulting only in 9-hydroxyphenalenone, the original PLY ligand (see details in ESI†). This observation along with the deuterium labelling studies using PhSiD_3_ fully confirm that the additional hydrogen atom in the PLY backbone of dimer **5** originates exclusively from the silane molecule.

However, under the current scenario we do not fully refute a transient Co–H formation from where the hydride migrates to the ligand backbone. Notably, multiple attempts defied the isolation or obtaining of some spectroscopic signature of any cobalt hydride formed during the reaction. Most likely, the formation of a C–H bond in the PLY backbone is direct and does not invoke the formation of the Co–H bond at first. It is important to note that the isolated σ-dimer **5** only forms when the reaction is quenched and such dimerization does not happen during the normal course of the catalytic reaction.

Taking all trapped intermediates into account, we delineate the plausible mechanism described in Scheme 2. As a first step, the biradical **2** cleaves a Si–H bond *via* a SET process generating a silyl-radical (trapped and characterized by mass spectrometry) and H^–^. Next, the reducing equivalent (H^–^, generated *in situ*) is stored in the PLY ligand of **6***via* a dearomatization process. Examples are only scarce where the ligand exercises such a paramount role. As was proposed by Hammes-Schiffer, a phlorin intermediate formed instead of a prototypical nickel hydride in the case of Ni-porphyrin based hydrogen evolution catalyst.^54^ In another notable example, Gray reported a [η^4^-Cp*H] species formed *in lieu* of the rhodium hydride, during their study of a hydrogen evolution catalysis.^55^ Storing the redox equivalent in the form of a C–H bond which can break reversibly also resembles storing redox potential in a C–C bond *via* pyridine dimerization that was documented by Holland *et al.* for a (NacNac)Fe molecule.^56^ Our present work not only demonstrates the rather surprising role of the PLY-ligand to store the reducing equivalent, but also steers its delivery to perform multi-electron reduction which is conceptually similar to the way NAD(P)H functions *via* dearomatization of a pyridine ring. The intermediate **6** further undergoes a hydrogen atom transfer process to give **7***via* combination of a silyl-radical to the acid substrate and regenerates the active catalyst **2**. Next, **7** can undergo silanol elimination (evidenced by ^1^H and ^29^Si NMR spectroscopies) to yield aldehyde, **8** (detected by ^1^H NMR spectrum). The aldehyde, **8** can undergo further reduction *via* another catalytic cycle (likewise cycle 1) to generate the hydrosilylated product **9** which upon subsequent hydrolysis produces corresponding alcohol. Very notably, traditional precious metal catalyzed hydrosilylation reactions are believed to mediate *via* Chalk–Harrod,^57^ modified Chalk–Harrod or Ojima^58^ pathways where a combination of steps such as oxidative addition of Si–H to the metal, insertion of carbonyl to M–SiR_3_ and reductive elimination of the silyl ether is common. Our proposed mechanism is strikingly different from these much-conceived pathways and showcases the pivotal role of the ligand-based redox process in hydrosilylation.

## Conclusions

To summarize, in the thriving history of PLY based chemistry, for the first time we uncover that the reducing equivalent can be stored in the PLY backbone in the form of a C–H bond and can be transferred catalytically to the organic substrates in executing the reduction process. The redox active PLY backbone attached with Co(ii) ion has been used to store and transfer electrons to achieve reduction of carboxylic acids under mild conditions. Several years back, Chirik and Wieghardt envisaged the paradigm shift in redox-active ligand-based coordination chemistry in tuning metal mediated redox chemistry to inspire new catalytic transformations.^59^ In our current study, the role of redox-active PLY based ligand expands beyond just tuning the metal mediated redox chemistry but showcases a paramount role of the redox-active backbone *via* direct participation in the catalytic process.

## Experimental section

### General considerations

The 9-hydroxyphenalenone (PLY) ligand was prepared following the reported literature procedure.^60^ All manipulations were carried out using standard Schlenk techniques, high-vacuum and glovebox maintained below 0.1 ppm of O_2_ and H_2_O. All glassware were oven-dried at 130 °C and evacuated while hot prior to use. All solvents were distilled from Na/benzophenone prior to use. All other chemicals were purchased from Sigma Aldrich and used as received. Elemental analyses were carried out using a PerkinElmer 2400 CHN analyzer and samples were prepared by being kept under reduced pressure (10^–2^ mbar) overnight. Analytical TLC was performed on a Merck 60F254 silica gel plate (0.25 mm thickness). FT-IR spectra were recorded by a PerkinElmer FT-IR spectrometer. NMR spectra were recorded on a JEOL ECS 400 MHz spectrometer and on a Bruker Avance III 500 MHz spectrometer. All chemical shifts were reported in ppm using tetramethylsilane as a reference. Crystallographic data for structural analysis of **1** and **5** (the σ–dimer) were deposited at the Cambridge Crystallographic Data Center, CCDC number ; 1828004 and ; 1846773.†

### Procedure for the synthesis of Co(PLY-O,O)_2_(THF)_2_ (**1**)

To a hot solution of 9-hydroxyphenalenone (0.98 g; 5.0 mmol) in acetonitrile (50.0 mL), a methanolic (20.0 mL) solution of Co(OAc)_2_·4H_2_O (0.622 g; 2.5 mmol) was added dropwise over 5 min and immediately a maroon colored precipitate was observed. The reaction mixture was refluxed for 3 h and then it was cooled to room temperature. The maroon colored precipitate was washed with methanol followed by acetonitrile (5 times) to remove any unreacted ligand and metal salt, and then the residue was collected, and vacuum-dried. The maroon color residue was stirred in dry THF at room temperature for 12 h; deep red color block shaped X-ray quality crystals were grown from saturated solution of THF at 4 °C within 10–12 days. Yield: 1.008 g (68%). ESI-MS: *m*/*z* calc. for C_34_H_30_CoO_6_K [M + K]^+^ 632.1011, found 632.1015. Anal. calcd for C_34_H_30_CoO_6_: C: 68.80, H: 5.09; found: C: 68.84, H: 5.14. UV-vis (THF) *λ*_max_/nm (*ε* in M^–1^ cm^–1^ lit): 258 (33 350), 284 (18 310), 356 (26 620), 414 (8867), 438 (9270). FT-IR (thin film) *ν* (cm^–1^): 3742, 2985, 1776, 1602, 1513, 1258, 1096, 749.

### General method for carboxylic acid reduction

Inside a N_2_ filled glovebox, an oven dried reaction tube was charged with catalyst **1** (14.8 mg, 0.025 mmol, 5 mol%), K (1.95 mg, 0.05 mmol, 10 mol%) and dry THF (1.5 mL). The reaction mixture was stirred at room temperature for 4 h to generate the green colored doubly reduced product **2**. Subsequently PhSiH_3_ (123.3 μL, 1.0 mmol) was added to the reaction mixture followed by the addition of the appropriate carboxylic acid (0.5 mmol). The reaction mixture was stirred at room temperature for another 20 h and after completion of the reaction, hydrolysis of the silylated product was performed by the dropwise addition of 1.5 mL 2 (M) NaOH solution. Next, the aqueous phase was extracted with Et_2_O/EtOAc (15 mL). The organic layer was washed with brine and dried over MgSO_4_. The solvent was removed under reduced pressure and the desired product was purified by flash chromatography on silica gel.

### Computational details

All calculations were carried out using Density Functional Theory as implemented in the Gaussian 09 (ref. 61) quantum chemistry programs. The geometries of the stationary points were optimized with the generalized gradient approximation (GGA) by means of the Becke exchange functional along with Lee, Yang, Parr correlation functional (LYP). We used the double-ζ basis set with the relativistic effective core potential of Hay and Wadt (LANL2DZ) for the cobalt atom and the 6-31+G(d) basis set for other elements (H, C, O). The geometries were optimized without any symmetry constraints. For the optimization, the full model was chosen with furan as the weakly coordinating ligand. The symmetry broken DFT solution was detected using the Gaussian keyword stable = opt. Harmonic force constants were computed at the optimized geometries to characterize the stationary points as minima. The molecular orbitals were visualized and the spin density was plotted using Gaussview.

### X-ray crystallographic details

Single crystals of compounds **1** and **5** were mounted on a glass tip. Intensity data were collected on a SuperNova, Dual, Mo at zero, Eos diffractometer. The crystals were kept at 100 K during data collection. Atomic coordinates, isotropic and anisotropic displacement parameters of all the non-hydrogen atoms of two compounds were refined using Olex2,^62^ and the structure was solved with the Superflip^63^ structure solution program using Charge Flipping and refined with the ShelXL^64^ refinement package using Least Squares minimization. Structure graphics shown in the figures were created using the Olex2 and X-Seed software package version 2.0.^65^

### SQUID details

The SQUID magnetometer (Quantum Design MPMS) was used to investigate the magnetic properties (magnetic susceptibility) of the compounds. We used a lightweight homogeneous quartz tube as a sample holder for the magnetic measurements in SQUID MPMS-XL5 to minimize the background noise and stray field effects. The magnetic data were corrected for the diamagnetic contribution from the sample holder by measuring the magnetic moment of the sample holder with an air gap corresponding to the sample length. The intrinsic diamagnetism of the samples was corrected by the standard literature using Pascal's constants.

## Conflicts of interest

There are no conflicts to declare.

## Supplementary Material

Supplementary information

Crystal structure data
