# Supplementary material for: Storing redox equivalent in the phenalenyl backbone towards catalytic multi-electron reduction
Source: Chem Sci. 2019 Jun 10;10(31):7433–41. doi: 10.1039/c9sc02057h (PMC6713874; doi:10.1039/c9sc02057h)
Supplement: Supplementary file 1 [file SC-010-C9SC02057H-s001.pdf]

Electronic Supplementary Material (ESI) for Chemical Science.
This journal is © The Royal Society of Chemistry 2019

Storing redox equivalent in the phenalenyl backbone towards catalytic multi-electron reduction of carboxylic acids

Mrinal Bhunia[1], Sumeet Ranjan Sahoo[1], Bikash Kumar Shaw[1], Shefali Vaidya[2], Anand Pariyar[1], Gonela Vijaykumar[1], Debashis Adhikari[2*] and Swadhin K. Mandal[1*]

[1]Department of Chemical Sciences, Indian Institute of Science Education and Research-Kolkata, Mohanpur-741246, India

[2]Department of Chemical Sciences, Indian Institute of Science Education and Research Mohali, SAS Nagar 140306, India

Contents

1. **Materials and methods……………………………………………………………….S2**
2. **Synthesis of cobalt complex, 1………………...S2**
3. **Synthesis of mono reduced (1A) and doubly reduced cobalt complex (2)..………S3**
4. **Procedure for reaction condition optimization of benzoic acid reduction……….S4**
5. **General Method for Reduction of Carboxylic Acids……………………………….S5-S16**
6. **Solid state magnetic data of 2………………………………………………………...S17**
7. **Detailed characterization of 1 and 2…………………………………………………S18**
8. **C-H….π interaction in complex 1……………………………………………………S19**
9. **Control experiments for mechanistic investigation………………………………..S19-S25**
10. **X-ray crystallographic details………………………………………………………S25-S27**
11. **NMR data of benzyl alcohol derivatives after reduction of various carboxylic acids......S28-S50**
12. **Spectroscopic Characterization of stoichiometric reaction……………………......S51-S66**
13. **Computational details and coordinates of the calculated structures………………...S67-S73**
14. **References……………………………………………………………………………..S74-S76**

1. **Materials and Methods.**

The 9-hydroxyphenalenone (PLY) ligand was prepared following the reported literature procedure (SI, Figure S57). All manipulations were carried out using standard Schlenk techniques, high-vacuum and also glovebox techniques maintained below 0.1 ppm of O_2 and H_2O. All glasswares were oven-dried at 130 °C and evacuated while hot prior to use. All solvents were distilled from Na/benzophenone prior to use. All other chemicals were purchased from Sigma Aldrich and used as received. Elemental analyses were carried out using a Perkin-Elmer 2400 CHN analyzer and samples were prepared by keeping under reduced pressure (10^{-2} mbar) for overnight. Analytical TLC was performed on a Merck 60F254 silica gel plate (0.25 mm thickness). FT-IR spectra were recorded by transmission measurements of thin film with a PerkinElmer FT-IR spectrometer Spectrum RXI. NMR spectra were recorded on a JEOL ECS 400 MHz spectrometer and on a Bruker Avance III 500 MHz spectrometer. All chemical shifts were reported in ppm using tetramethylsilane as a reference. Crystallographic data for structural analysis of **1** and **5** (σ–dimer) were deposited at the Cambridge Crystallographic Data Center, CCDC number 1828004 and 1846773. These data can be obtained free of charge from the Cambridge Crystallographic Data Center. Magnetic susceptibility measurements were performed using a Quantum Design MPMS-XL SQUID magnetometer. A light weight homogeneous quartz tube as a sample holder for the magnetic measurements in SQUID MPMS-XL5 to minimize the background noise and stray field effects. The magnetic data were corrected for the diamagnetic contribution from the sample holder by measuring the magnetic moment of the sample holder with an air gap corresponding to the sample length. The intrinsic diamagnetism of the samples was corrected by the standard literature using Pascal's constants.

2. **Synthesis of $Co(PLY\text{-}O,O)_2(THF)_2$ (1).**

To a hot solution of 9-hydroxyphenalenone (0.98 g; 5.0 mmol) in acetonitrile (50.0 mL), methanolic (20.0 mL) solution of $Co(OAc)_2.4H_2O$ (0.622 g; 2.5 mmol) was added dropwise over 5 min and immediately maroon color precipitate was observed. The reaction mixture was refluxed for 3 h and then it was cooled to room temperature. The maroon colored precipitate was washed with methanol followed by acetonitrile (5 times) to remove any unreacted ligand and metal salt, and then the residue was collected, and vacuum-dried. The maroon color residue was stirred in dry THF at room temperature for 12 h; deep red color block shaped X-ray quality crystals were grown from saturated solution of THF at 4 °C within 10-12 days. Yield: 1.008 g (68%). ESI-MS: m/z calc. for $C_{34}H_{30}CoO_6K$ $[M+K]^+$ 632.1011, found 632.1015. Analytically calcd for $C_{34}H_{30}CoO_6$: C: 68.80, H: 5.09; found: C: 68.84, H: 5.14. UV−vis (THF) λ_{max}/nm (ε in M^{-1} cm^{-1} lit): 258 (33350), 284 (18310), 356 (26620), 414 (8867), 438 (9270). FT-IR (thin film) ν (cm^{-1}): 3742, 2985, 1776, 1602, 1513, 1258, 1096, 749.

3. **Synthesis of mono and doubly reduced cobalt complex**.

Synthesis of mono-reduced cobalt complex, [Co^{II}(PLY)$_2^-$(THF)]K$^+$ (1A). In an oven dried 25 mL Schlenk flask, complex **1** (100 mg, 0.1685 mmol) and K (6.59 mg, 0.1685 mmol, 1 equivalent) were charged inside the glovebox and 3.0 mL dry THF was added to it. Then the reaction mixture was stirred at room temperature for 6 h and during the course of reaction, color changed from maroon to yellowish-green. The solvent was evaporated in high vacuum and the yellowish-green colored precipitate was obtained in 94% yield.

UV−vis (THF) λ_{max}/nm (ε in M^{-1} cm^{-1} lit): 256 (9900), 348.3 (7158), 416 (2399), 440 (2599).

FT-IR (thin film) ν (cm^{-1}): 1607, 1564, 1503, 1399, 1321, 1223, 1121, 960.

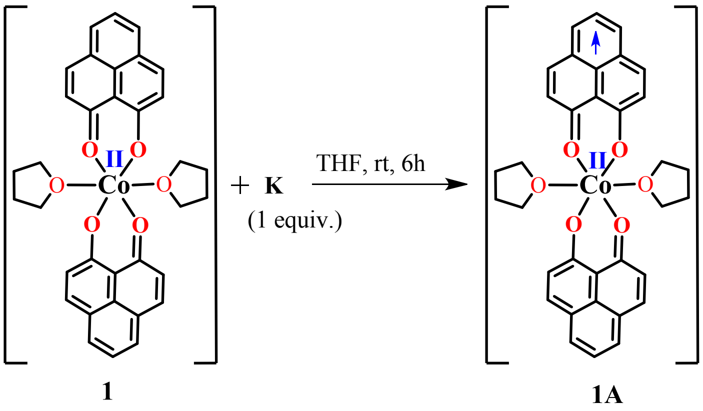

Synthesis of doubly reduced cobalt complex, [Co^{II}(PLY)$_2^{2-}$(THF)]K^{2+} (2). In an oven dried 25 mL Schlenk flask, complex **1** (150 mg, 0.2527 mmol) and K (19.76 mg, 0.5054 mmol, 2 equivalent) were charged inside the glovebox and 4.0 mL dry THF was added to it. Then the reaction mixture was stirred at room temperature for 10 h and maroon color of the reaction mixture was changed to green color during the course of reaction. The solvent was evaporated in high vacuum and the green colored precipitate was obtained in 92% yield. Despite several attempts crystallization of **2** failed due to sensitivity of the molecule.

UV−vis (THF) λ_{max}/nm (ε in M^{-1} cm^{-1} lit): 258 (10000), 346 (5606), 434 (2723), 450 (2724).

FT-IR (thin film) ν (cm^{-1}): 1611, 1566, 1519, 1391, 1323, 1213, 1119, 958.

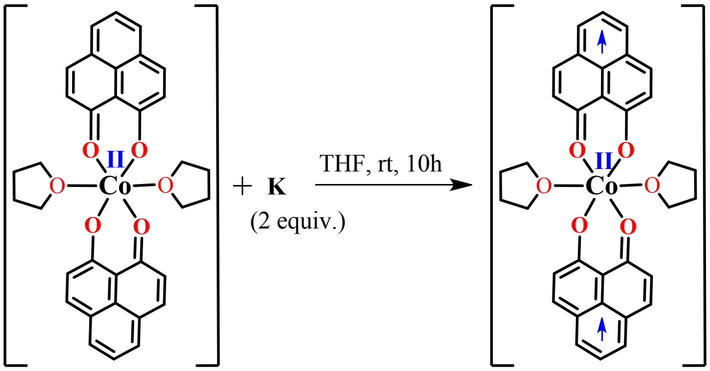

1 **2**

4. **Procedure for reaction condition optimization of benzoic acid reduction**.

In a nitrogen-filled glovebox, an oven dried reaction tube was charged with **1** (14.83 mg, 0.025 mmol) and K (1.95 mg, 0.05 mmol) followed by the addition of THF (1.0 mL) and the reaction mixture was stirred at room temperature for 4 h to generate **2**. Then $PhSiH_3$ (123.3 μL, 1.0 mmol) was added to the

Table S1. Optimization of the reaction conditions for the reduction of benzoic acid **3a**.[a]

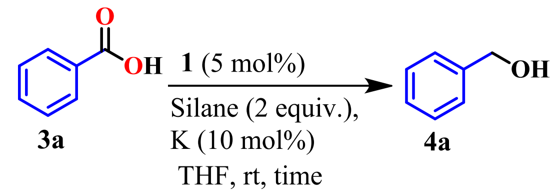

Entry	Catalyst	Silane	Time (h)	Yield (%)[b]
1	**1**	$PhSiH_3$	30	99
2	**1**	PMHS	30	—
3	**1**	Ph_2SiH_2	30	26
4	**1**	Ph_3SiH	30	—
5	**1**	**$PhSiH_3$**	**24**	**99 (92)**
6[c]	**1**	$PhSiH_3$	7	94
7	**1**	$PhSiH_3$ (1)	7	33
8[d]	—	$PhSiH_3$	24	—
9[e]	**1**	$PhSiH_3$	24	—
10	$CoCl_2$	$PhSiH_3$	24	—
11	$CoCl_2$ with K	$PhSiH_3$	24	—
12	$Co(acac)_2$	$PhSiH_3$	24	—
13	$Co(acac)_2$ with K	$PhSiH_3$	24	—
14[f]	**1**	$PhSiH_3$	24	—
15[g]	**1**	$PhSiH_3$	24	11

[a]Reaction conditions: Catalyst **1** (5.0 mol %), K (10.0 mol%), **3a** (0.5 mmol), silane (1.0 mmol, 2.0 equiv.), THF (1.0 mL), rt. Hydrolysis was performed with 2.0 (M) NaOH solution. [b] The yields were determined from ^{1}H NMR spectroscopy using *m*-xylene as an internal standard. [c] 6% PhCHO was also obtained. [d] Only K without catalyst **1**. [e] No K. [f] Reaction with TDAE as a reductant. [g] Reaction with Na.

reaction mixture followed by the addition of benzoic acid (61 mg, 0.5 mmol). Then the reaction mixture was stirred at room temperature for different time interval and the reaction mixture was hydrolyzed by drop-wise addition of 1.5 mL 2 M NaOH solutions and stirred at room temperature for another 3 h. Then the reaction mixture was diluted with water and the aqueous phase was extracted with Et_2O (15 mL). The organic layer was washed with brine and dried over $MgSO_4$. The solvent was removed under reduced pressure and the yields were determined from ^{1}H NMR spectroscopy using *m*-xylene as an internal standard. The desired product (benzyl alcohol) was purified by flash chromatography on silica gel.

5. **General Method for Reduction of Carboxylic Acids**.

Inside a N_2 filled glovebox, an oven dried reaction tube was charged with catalyst **1** (14.8 mg, 0.025 mmol, 5 mol%), K (1.95 mg, 0.05 mmol, 10 mol%) and dry THF (1.5 mL) and the reaction mixture was stirred at room temperature for 4 h to generate **2**. Then $PhSiH_3$ (123.3 μL, 1.0 mmol) was added to the reaction mixture followed by addition of the corresponding carboxylic acids (0.5 mmol). The reaction mixture was stirred at room temperature for another 20 h and after completion of the reaction, the hydrosilylated product was hydrolyzed by drop-wise addition of 1.5 mL 2 M NaOH solutions. Then, the aqueous phase was extracted with Et_2O/EtOAc (15 mL). The organic layer was washed with brine and dried over $MgSO_4$. The solvent was removed under reduced pressure and the desired product was purified by flash column chromatography on silica gel.

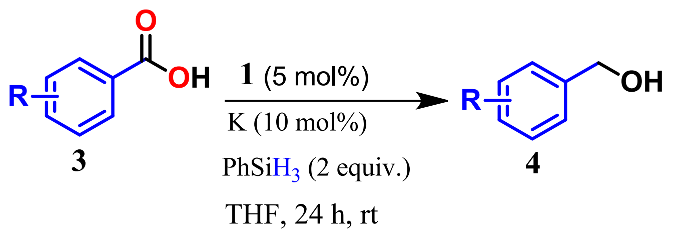

Scheme S1. Reduction of benzoic acid catalyzed by **1**

Benzyl alcohol (4a) .[S1, S2]

The general procedure was followed for the synthesis of benzyl alcohol, **4a**. The reaction was performed with benzoic acid, **3a** (61.1 mg, 0.5 mmol), $PhSiH_3$ (123.3 μL, 1.0 mmol), **1** (14.8 mg, 0.025 mmol, 5 mol%), K (1.95 mg, 0.05 mmol, 10 mol%) and dry THF (1.5 mL). The desired product benzyl alcohol, **4a** (49.2 mg, 91% yield) was isolated as colorless liquid after purification from flash column chromatography using hexane and Et_2O (3:1) as eluent.

^1H NMR (400 MHz, $CDCl_3$): δ 7.38 – 7.28 (m, 5H), 4.63 (s, 2H) ppm.
$^{13}C\{^1H\}$ NMR (100 MHz, $CDCl_3$): δ 140.7, 128.4, 127.4, 126.9, 64.9 ppm.

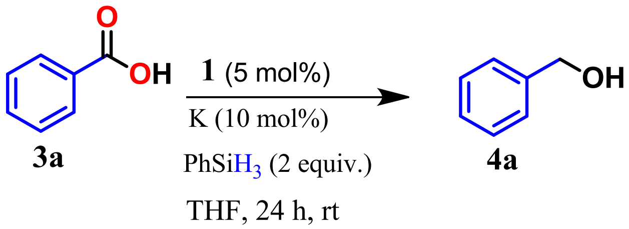

4-Methylbenzyl alcohol (4b).[S3]

The general procedure of carboxylic acid reduction was followed. The reaction was performed with 4-methylbenzoic acid, **3b** (68.1 mg, 0.5 mmol), $PhSiH_3$ (123.3 μL, 1.0 mmol), **1** (14.8 mg, 0.025 mmol, 5 mol%), K (1.95 mg, 0.05 mmol, 10 mol%) and dry THF (1.5 mL). The desired product 4-methylbenzyl alcohol, **4b** (51.9 mg, 85% yield) was isolated as light-yellow oil after purification from flash column chromatography using hexane and ethyl acetate (10:2) mixture as eluent.

^1H NMR (400 MHz, $CDCl_3$): δ 7.25 (d, J = 8.0, 2H), 7.17 (d, J = 8.0, 2H), 4.63 (s, 2H), 2.35 (s, 3H) ppm.
$^{13}C\{^1H\}$ NMR (100 MHz, $CDCl_3$): δ 137.8, 137.4, 129.2, 127.1, 65.2, 21.1 ppm.

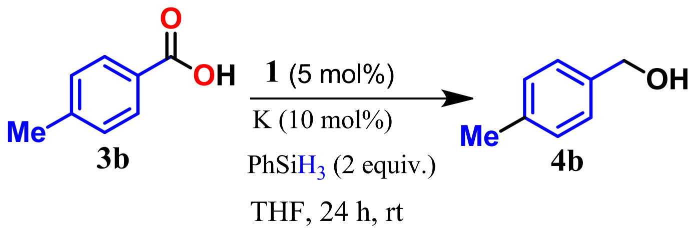

4-Methoxybenzyl alcohol (4c).[S1, S4]

The general procedure of carboxylic acid reduction was followed. The reaction was performed with 4-methoxybenzoic acid, **3c** (76.1 mg, 0.5 mmol), $PhSiH_3$ (123.3 μL, 1.0 mmol), **1** (14.8 mg, 0.025 mmol, 5 mol%), K (1.95 mg, 0.05 mmol, 10 mol%) and dry THF (1.5 mL). The desired product 4-methoxybenzyl

alcohol, **4c** (57.3 mg, 83% yield) was isolated as light-yellow oil after purification from flash column chromatography using hexane and Et_2O (3:1) mixture as eluent.

^1H NMR (400 MHz, $CDCl_3$): δ 7.25 (d, J = 8.8 Hz, 2H), 6.86 (d, J = 8.4 Hz, 2H), 4.56 (s, 2H), 3.78 (s, 3H), ppm. $^{13}C\{^1H\}$ (100 MHz, $CDCl_3$): δ 159.1, 133.1, 128.6, 113.8, 64.8, 55.2 ppm.

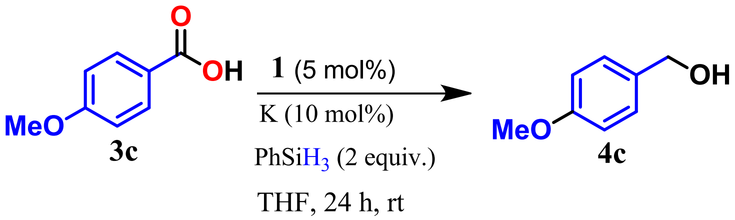

4-Chlorobenzyl alcohol (4d).[S4]

The general procedure of carboxylic acid reduction was followed. The reaction was performed with 4-chlorobenzoic acid, **3d** (78.3 mg, 0.5 mmol), $PhSiH_3$ (123.3 µL, 1.0 mmol), **1** (14.8 mg, 0.025 mmol, 5 mol%), K (1.95 mg, 0.05 mmol, 10 mol%) and dry THF (1.5 mL). The desired product 4-chlorobenzyl alcohol, **4d** (37.1 mg, 54% yield) was isolated as white solid after purification from flash column chromatography using hexane and Et_2O (3:2) mixture as eluent.

^1H NMR (400 MHz, $CDCl_3$): δ 7.27 (d, J = 8.4 Hz, 2H), 7.22 (d, J = 8.4 Hz, 2H), 4.58 (s, 2H), 2.02 (bs, 1H) ppm.

$^{13}C\{^1H\}$ (100 MHz, $CDCl_3$): δ 139.2, 133.3, 128.6, 128.2, 64.5 ppm.

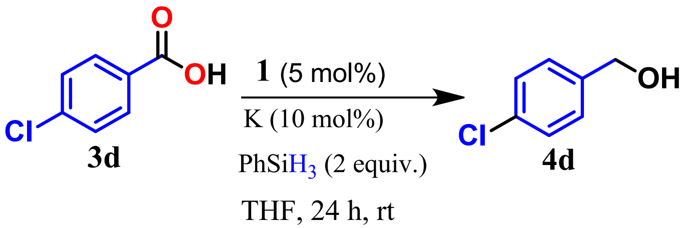

4-Bromobenzyl alcohol (4e).[S5]

The general procedure was followed. The reaction was performed with 4-bromobenzoic acid, **3e** (100.5 mg, 0.5 mmol), $PhSiH_3$ (123.3 µL, 1.0 mmol), **1** (14.8 mg, 0.025 mmol, 5 mol%), K (1.95 mg, 0.05 mmol, 10 mol%) and dry THF (1.5 mL). The desired product 4-bromobenzyl alcohol, **4e** (67.3 mg, 72% yield) was isolated as pale yellow solid after purification from flash column chromatography using hexane and Et_2O (3:2).

^{1}H NMR (400 MHz, $CDCl_3$): δ 7.45 (d, J = 8.4, 2H), 7.18 (d, J = 8.4, 2H), 4.57 (s, 2H), 2.58 (br. s, 1H) ppm.

$^{13}C\{^{1}H\}$ NMR (100 MHz, $CDCl_3$): δ 139.6, 131.5, 128.5, 121.3, 64.3 ppm.

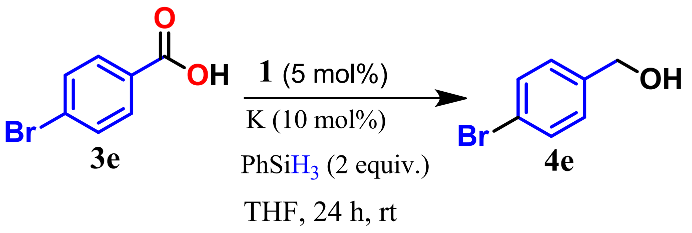

4-Iodobenzyl alcohol (4f). [S6, S7]

The general procedure was followed. The reaction was performed with 4-iodobenzoic acid, **3f** (124.0 mg, 0.5 mmol), $PhSiH_3$ (123.3 µL, 1.0 mmol), **1** (14.8 mg, 0.025 mmol, 5 mol%), K (1.95 mg, 0.05 mmol, 10 mol%) and dry THF (1.5 mL). The desired product 4-iodobenzyl alcohol, **4f** (106.5 mg, 91% yield) was isolated as white solid after purification from flash column chromatography using hexane and Et_2O (3:2) mixture as eluent.

^{1}H NMR (400 MHz, $CDCl_3$): δ 7.68 (d, J = 8.4 Hz, 2H), 7.10 (d, J = 8.4 Hz, 2H), 4.63 (s, 2H) ppm.

$^{13}C\{^{1}H\}$ NMR (100 MHz, $CDCl_3$): δ 140.4, 137.6, 128.8, 93.0, 64.6 ppm.

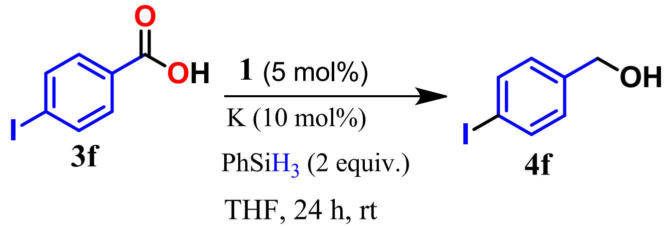

4-Trifluoromethylbenzyl alcohol (4g).[S1]

The general procedure was followed. The reaction was performed with 4-trifluoromethylbenzoic acid, **3g** (95.0 mg, 0.5 mmol), $PhSiH_3$ (123.3 µL, 1.0 mmol), **1** (14.8 mg, 0.025 mmol, 5 mol%), K (1.95 mg, 0.05 mmol, 10 mol%) and dry THF (1.5 mL). The desired product 4-trifluoroethylbenzyl alcohol, **4g** (67.8 mg, 77% yield) was isolated as white solid after purification from flash column chromatography using hexane and Et_2O (3:2) mixture as eluent.

^{1}H NMR (400 MHz, $CDCl_3$): δ 7.60 (d, J = 8.0 Hz, 2H), 7.45 (d, J = 7.6 Hz, 2H), 4.74 (s, 2H) ppm.
^{13}C NMR (100 MHz, $CDCl_3$): δ 144.7, 129.7 (q, J = 32.2 Hz), 126.8, 125.4 (q, J = 3.8 Hz), 122.8, 64.4 ppm.

^{19}F NMR (376 MHz, $CDCl_3$): δ -62.4 ppm.

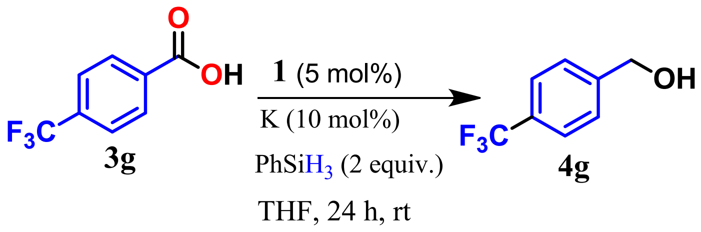

4-Nitrobenzyl alcohol (4h).[S2, S8]

The general procedure of carboxylic acid reduction was followed. The reaction was performed with 4-nitrobenzoic acid, **3h** (83.6 mg, 0.5 mmol), $PhSiH_3$ (123.3 μL, 1.0 mmol), **1** (14.8 mg, 0.025 mmol, 5 mol%), K (1.95 mg, 0.05 mmol, 10 mol%) and dry THF (1.5 mL). The desired product 4-nitrobenzyl alcohol, **4h** (66.6 mg, 87% yield) was isolated as amorphous yellow solid after purification from flash column chromatography using hexane and ethyl acetate (10:3) mixture as eluent.

^{1}H NMR ($CDCl_3$, 400 MHz): δ 8.20 (d, J = 8.8 Hz, 2H), 7.52 (d, J = 8.8 Hz, 2H), 4.83 (s, 2H) ppm.

$^{13}C\{^{1}H\}$ NMR ($CDCl_3$, 100 MHz): δ 148.2, 147.3, 127.0, 123.7, 63.9 ppm.

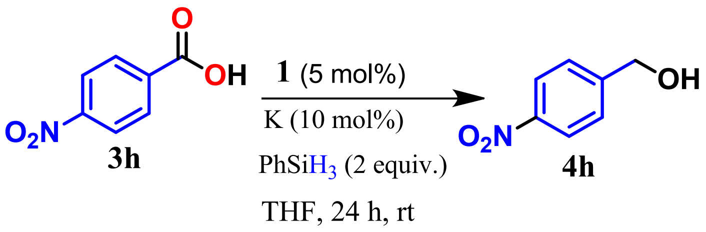

4-Cyanobenzyl alcohol (4i).[S5]

The general procedure of carboxylic acid reduction was followed. The reaction was performed with 4-cyanobenzoic acid, **3i** (73.6 mg, 0.5 mmol), $PhSiH_3$ (123.3 μL, 1.0 mmol), **1** (14.8 mg, 0.025 mmol, 5 mol%), K (1.95 mg, 0.05 mmol, 10 mol%) and dry THF (1.5 mL). The desired product 4-cyanobenzyl alcohol, **4i** (51.2 mg, 81% yield) was isolated as amorphous white solid after purification from flash column chromatography using hexane and ethyl acetate (10:3).

^{1}H NMR (400 MHz, $CDCl_3$): δ 7.62 (d, J = 8.0 Hz, 2H), 7.47 (d, J = 8.0 Hz, 2H), 4.77 (s, 2H), 2.36 (bs, 1H) ppm.

$^{13}C\{^{1}H\}$ NMR (100 MHz, $CDCl_3$): δ 146.1, 132.3, 127.0, 118.8, 111.1, 64.3 ppm.

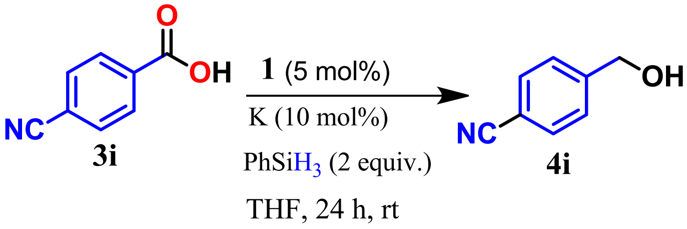

Mixture of 4-(hydroxymethyl)benzaldehyde/ 1,4-phenylenedimethanol = (73:27) (4j).[S9]

The general procedure was followed. The reaction was performed with 4-formylbenzoic acid, **3j** (75.1 mg, 0.5 mmol), $PhSiH_3$ (123.3 µL, 1.0 mmol), **1** (14.8 mg, 0.025 mmol, 5 mol%), K (1.95 mg, 0.05 mmol, 10 mol%) and dry THF (1.5 mL). The desired product 4-formylbenzyl alcohol, **4j** (49.7 mg, 73% yield) was isolated as amorphous yellow solid after purification from flash column chromatography using hexane and ethyl acetate (5:1).

^{1}H NMR (400 MHz, $CDCl_3$): δ 10.01 (s, 1H; CHO), 7.88 (d, J = 8.0 Hz, 2H; aldol), 7.54 (d, J = 8.0 Hz, 2H; aldol), 7.37 (s, 4H; aromatic protons; diol), 4.81 (s, 2H; CH_2; aldol), 4.70 (s, 4H; CH_2; diol) ppm.

$^{13}C\{^{1}H\}$ NMR (100 MHz, $CDCl_3$): δ 192.0 (CHO), 147.7 (aromatic carbons; aldol), 140.3 (2 aromatic carbons; diol), 134.9 (aromatic carbons; aldol), 130.0 (aromatic carbons; aldol), 126.9 (4 aromatic carbons; diol), 126.6 (aromatic carbons; aldol), 65.1 (2 CH_2; diol), 64.6 (CH_2; aldol) ppm.

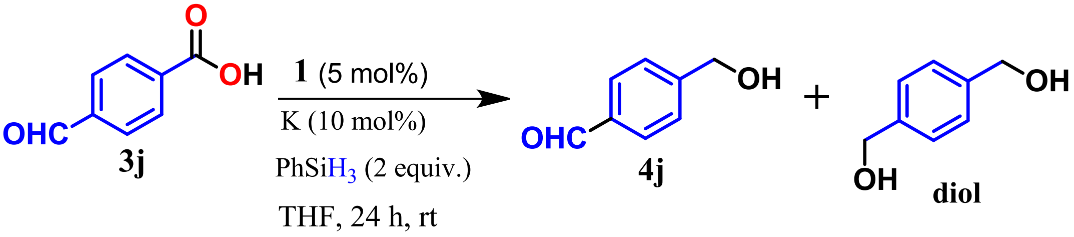

4-Vinylbenzyl alcohol (4k).[S10]

The general procedure of carboxylic acid reduction was followed. The reaction was performed with 4-vinylbenzoic acid, **3k** (74.1 mg, 0.5 mmol), $PhSiH_3$ (123.3 µL, 1.0 mmol), **1** (14.8 mg, 0.025 mmol, 5 mol%), K (1.95 mg, 0.05 mmol, 10 mol%) and dry THF (1.5 mL). The desired product 4-vinylbenzyl alcohol, **4k** (42.9 mg, 67% yield) was isolated as white solid after purification from flash column chromatography using hexane and Et_2O (2:1) mixture as eluent.

^{1}H NMR (400 MHz, $CDCl_3$): δ 7.41 (d, J = 8.0 Hz, 2H), 7.32 (d, J = 8.4 Hz, 2H), 6.72 (dd, J = 10.8, 6.8 Hz, 1H), 5.75 (d, J = 17.6 Hz, 1H), 5.25 (d, J = 10.8 Hz, 1H), 4.67 (s, 2H) ppm.

^{13}C NMR (100 MHz, $CDCl_3$): 140.4, 137.0, 136.4, 127.2, 126.4, 113.9, 65.1 ppm.

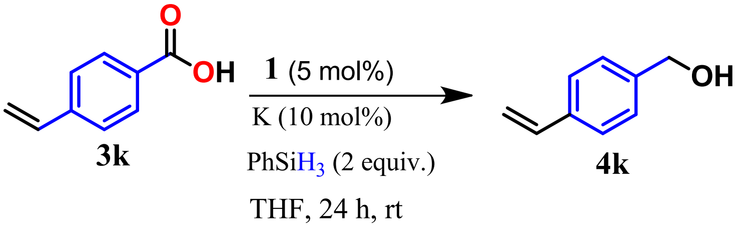

3-Methylbenzyl alcohol (4l).[S4]

The general procedure was followed. The reaction was performed with *m*-toluic acid, **3l** (68.1 mg, 0.5 mmol), $PhSiH_3$ (123.3 μL, 1.0 mmol), **1** (14.8 mg, 0.025 mmol, 5 mol%), K (1.95 mg, 0.05 mmol, 10 mol%) and dry THF (1.5 mL). The desired product 3-methylbenzyl alcohol, **4l** (52.5 mg, 86% yield) was isolated as light yellow oil after purification from flash column chromatography using hexane and ethyl acetate (5:1).

^{1}H NMR (400 MHz, $CDCl_3$): δ 7.23 (d, J = 7.6 Hz, 1H), 7.18 (s, 1H), 7.14 (d, J = 7.6 Hz, 1H), 7.10 (d, J = 7.6 Hz, 1H), 4.64 (s, 2H), 2.35 (s, 3H) ppm.

^{13}C{^{1}H} (100 MHz, $CDCl_3$): δ 140.8, 138.2, 128.5, 128.4, 127.7, 124.0, 65.4, 21.3 ppm.

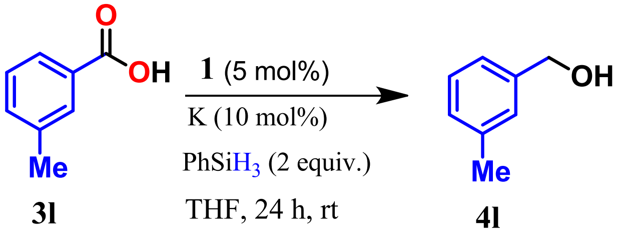

3-Bromobenzyl alcohol (4n).[S4]

The general procedure of carboxylic acid reduction was followed. The reaction was performed with 3-bromobenzoic acid, **3n** (100.5 mg, 0.5 mmol), $PhSiH_3$ (123.3 μL, 1.0 mmol), **1** (14.8 mg, 0.025 mmol, 5 mol%), K (1.95 mg, 0.05 mmol, 10 mol%) and dry THF (1.5 mL). The desired product 3-bromobenzyl alcohol, **4n** (75.8 mg, 85% yield) was isolated as yellow solid after purification from flash column chromatography using hexane and ethyl acetate (5:1) mixture as eluent.

^{1}H NMR (400 MHz, $CDCl_3$): δ 7.58 (s, 1H), 7.47 (d, J = 7.6 Hz, 1H), 7.34−7.28 (m, 2H), 4.71 (s, 2H), 2.10 (s, 1H) ppm.

$^{13}C\{^1H\}$ (100 MHz, $CDCl_3$): δ 143.0, 130.6, 130.1, 129.8, 125.3, 122.6, 64.4 ppm.

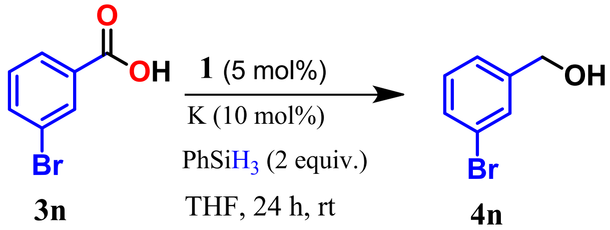

2-Methoxybenzyl alcohol (4o).[S11]

The general procedure was followed. The reaction was performed with 2-methoxybenzoic acid, **3o** (76.1 mg, 0.5 mmol), $PhSiH_3$ (123.3 µL, 1.0 mmol), **1** (14.8 mg, 0.025 mmol, 5 mol%), K (1.95 mg, 0.05 mmol, 10 mol%) and dry THF (1.5 mL). The desired product 2-methoxybenzyl alcohol, **4o** (47.6 mg, 72% yield) was isolated as colorless oil after purification from flash column chromatography using hexane and Et_2O (3:2).

^1H (400 MHz; $CDCl_3$): 7.30-7.26 (m, 2H), 6.95 (t, J = 7.6 Hz, 1H), 6.89 (d, J = 8.4 Hz, 1H), 4.69 (s, 2H), 3.87 (3H, s), 2.41 (bs, 1H) ppm.

$^{13}C\{^1H\}$ NMR (100 MHz; $CDCl_3$): 157.5, 129.0, 128.9, 128.8, 120.6, 110.2, 62.2, 55.2 ppm.

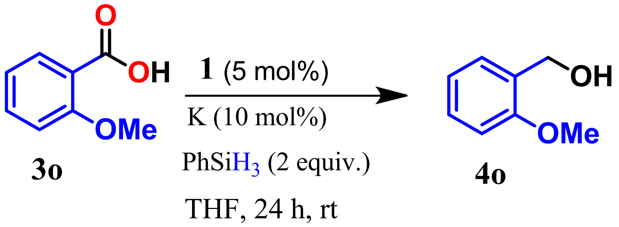

2-Chlorobenzyl alcohol (4p).[S4]

The general procedure was followed. The reaction was performed with 2-chlorobenzoic acid, **3p** (78.3 mg, 0.5 mmol), $PhSiH_3$ (123.3 µL, 1.0 mmol), **1** (14.8 mg, 0.025 mmol, 5 mol%), K (1.95 mg, 0.05 mmol, 10 mol%) and dry THF (1.5 mL). The desired product 2-chlorobenzyl alcohol**, 4p** (58.5 mg, 82% yield) was isolated as light yellow solid after purification from flash column chromatography using hexane and Et_2O (3:1).

^1H NMR (400 MHz, $CDCl_3$): δ 7.44 (d, J = 7.6 Hz, 1H), 7.32 (d, J = 7.6 Hz, 1H), 7.24−7.16 (m, 2H), 4.74 (s, 2H) ppm.

$^{13}C\{^1H\}$ (100 MHz, $CDCl_3$): δ 138.1, 132.7, 129.3, 128.8, 128.7, 127.0, 62.8 ppm.

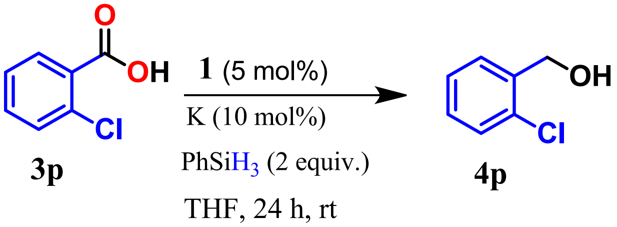

2-Bromobenzyl alcohol (4q).[S12]

The general procedure was followed. The reaction was performed with 2-bromobenzoic acid, **3q** (100.5 mg, 0.5 mmol), $PhSiH_3$ (123.3 μL, 1.0 mmol), **1** (14.8 mg, 0.025 mmol, 5 mol%), K (1.95 mg, 0.05 mmol, 10 mol%) and dry THF (1.5 mL). The desired product 2-bromobenzyl alcohol, **4q** (72.0 mg, 77% yield) was isolated as white solid after purification from flash column chromatography using hexane and Et_2O (3:1).

^{1}H-NMR (400 MHz, $CDCl_3$): δ 7.54 (1H, d, J = 8.0 Hz), 7.48 (1H, d, J = 7.6 Hz, Ar-H), 7.33 (1H, t, J = 7.2 Hz), 7.16 (1H, t, J = 7.2 Hz), 4.75 (2H, s) ppm.

^{13}C{^{1}H}-NMR (100 MHz, $CDCl_3$): δ 139.7, 132.6, 129.1, 128.9, 127.6, 122.5, 65.0 ppm.

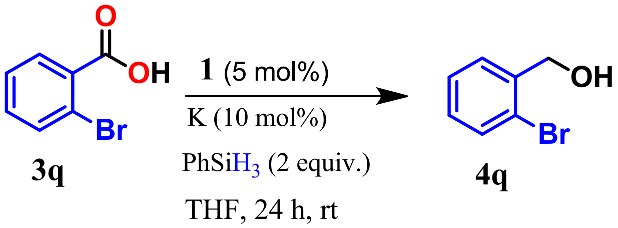

2-Iodobenzyl alcohol (4r).[S3]

The general procedure was followed. The reaction was performed with 2-iodobenzoic acid, **3r** (124.0 mg, 0.5 mmol), $PhSiH_3$ (123.3 μL, 1.0 mmol), **1** (14.8 mg, 0.025 mmol, 5 mol%), K (1.95 mg, 0.05 mmol, 10 mol%) and dry THF (1.5 mL). The desired product 2-iodobenzyl alcohol, **4r** (98.3 mg, 84% yield) was isolated as white solid after purification from flash column chromatography using hexane and Et_2O (3:1).

^{1}H NMR (400 MHz, $CDCl_3$): δ 7.82 (d, J = 8.4 Hz, 1H), 7.47–7.44 (m, 1H), 7.36 (t, J = 7.6 Hz, 1H), 7.00 (t, J=7.6 Hz, 1H), 4.67 (s, 2H) ppm.

^{13}C NMR {^{1}H} ($CDCl_3$): δ 142.6, 139.2, 129.3, 128.5, 128.4, 97.4, 69.3 ppm.

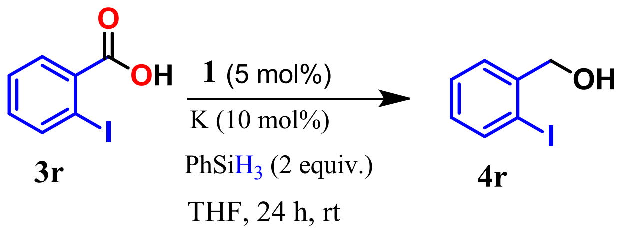

2,6-Dimethylbenzyl alcohol (4s).[S5, S13]

The title compound was prepared following the genera7l procedure with 2,6-dimethylbenzoic acid, **3s** (75.1 mg, 0.5 mmol), $PhSiH_3$ (123.3 μL, 1.0 mmol), **1** (14.8 mg, 0.025 mmol, 5 mol%), K (1.95 mg, 0.05 mmol, 10 mol%) and dry THF (1.5 mL). The desired product 2,6-dimethylbenzyl alcohol, **4s** (29.9 mg, 44% yield) was isolated as yellow solid after purification from flash column chromatography using hexane and Et_2O (3:1) mixture as eluent.

^{1}H NMR (400 MHz, $CDCl_3$): δ 7.13 - 7.09 (m, 1 H), 7.05 - 7.03 (m, 2 H), 4.74 (s, 2 H), 2.43 (s, 6 H) ppm.

^{13}C{^{1}H} NMR (100 MHz, $CDCl_3$): δ 137.3, 136.5, 128.4, 128.0, 59.3, 19.3 ppm.

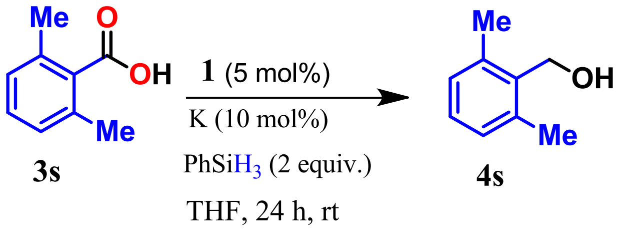

2,4,6-Trimethylbenzyl alcohol (4u).[S14]

The general procedure was followed for the title compound. The reaction was performed with 2,4,6-trimethylbenzoic acid, **3u** (82.1 mg, 0.5 mmol), $PhSiH_3$ (123.3 μL, 1.0 mmol), **1** (14.8 mg, 0.025 mmol, 5 mol%), K (1.95 mg, 0.05 mmol, 10 mol%) and dry THF (1.5 mL). The desired product 2,4,6-trimethylbenzyl alcohol, **4u** (39.1 mg, 52% yield) was isolated as colorless oil after purification from flash column chromatography using hexane and Et_2O (3:1).

^{1}H NMR (400 MHz, $CDCl_3$): δ 6.87 (s, 2H), 4.71 (s, 2H), 2.39 (s, 6H), 2.27 (s, 3H) ppm.

^{13}C{^{1}H} NMR (100 MHz, $CDCl_3$): δ 137.7, 137.3, 133.7, 129.1, 59.2, 20.9, 19.3 ppm.

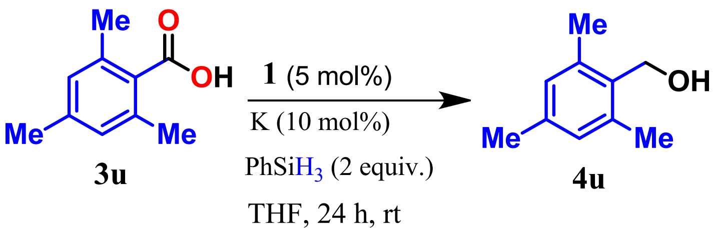

3u → **4u**

3,4,5-Trimethoxybenzyl alcohol (4v).[S1]

The general procedure was followed. The reaction was performed with 3,4,5-trimethoxybenzoic acid, **3v** (106.1 mg, 0.5 mmol), $PhSiH_3$ (123.3 μL, 1.0 mmol), **1** (14.8 mg, 0.025 mmol, 5 mol%), K (1.95 mg, 0.05 mmol, 10 mol%) and dry THF (1.5 mL). The desired product 3,4,5-trimethoxybenzyl alcohol, **4v** (85.2 mg, 86% yield) was isolated as light yellow oil after purification from flash column chromatography using hexane and Et_2O (3:1).

^1H NMR (400 MHz, $CDCl_3$): δ 6.61 (s, 2H), 4.64 (s, 2H), 3.87 (s, 6H), 3.84 (s, 3H) ppm.

$^{13}C\{^1H\}$ NMR (100 MHz, $CDCl_3$): δ 153.4, 137.4, 136.6, 125.5, 103.8, 65.6, 60.9, 56.1 ppm.

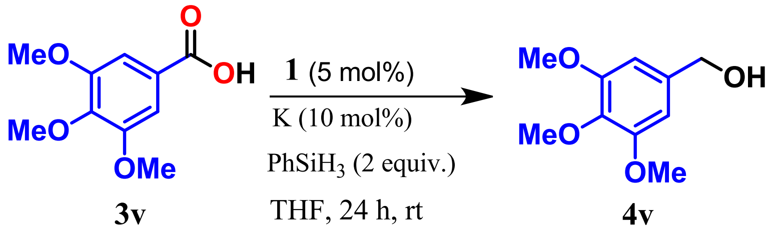

3v → **4v**

4-Chloro-3-nitrobenzyl alcohol (4w).[S15]

The general procedure was followed. The reaction was performed with 4-chloro-3-nitrobenzoic acid, **3w** (100.8 mg, 0.5 mmol), $PhSiH_3$ (123.3 μL, 1.0 mmol), **1** (14.8 mg, 0.025 mmol, 5 mol%), K (1.95 mg, 0.05 mmol, 10 mol%) and dry THF (1.5 mL). The desired product 4-chloro-3-nitrobenzyl alcohol, **4w** (59.1 mg, 63% yield) was isolated as amorphous pale yellow solid after purification from flash column chromatography using hexane and ethyl acetate (10:3).

^1H NMR (400 MHz, $CDCl_3$): δ 7.87 (s, 1H), 7.52 – 7.47 (m, 2H), 4.75 (s, 2H), 2.34 (brs, 1H) ppm.

$^{13}C\{^1H\}$ NMR (100 MHz, $CDCl_3$): δ 147.9, 141.4, 131.8, 131.0, 125.6, 123.4, 63.2 ppm.

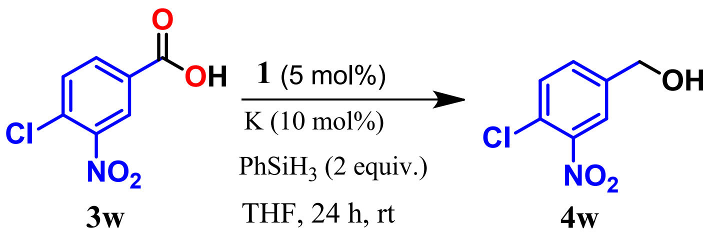

2-Chloro-5-nitrobenzyl alcohol (4x).[S16]

The general procedure was followed. The reaction was performed with 2-chloro-5-nitrobenzoic acid, **3x** (100.8 mg, 0.5 mmol), $PhSiH_3$ (123.3 μL, 1.0 mmol), 1 (14.8 mg, 0.025 mmol, 5 mol%), K (1.95 mg, 0.05 mmol, 10 mol%) and dry THF (1.5 mL). The desired product 2-chloro-5-nitrobenzyl alcohol, **4x** (66.6 mg, 71% yield) was isolated as off-white solid after purification from flash column chromatography using hexane and ethyl acetate (10:3).

^{1}H NMR (400 MHz, $CDCl_3$): δ 8.44 (d, J = 2.4 z, 1H), 8.08 (dd, J = 8.4, 2.8 Hz, 1H), 7.50 (d, J = 9.2 Hz, 1H), 4.85 (s, 2H) ppm.

^{13}C NMR {^{1}H} (100 MHz, $CDCl_3$): δ 146.9, 140.4, 138.7, 130.0, 123.3, 122.9, 61.7 ppm.

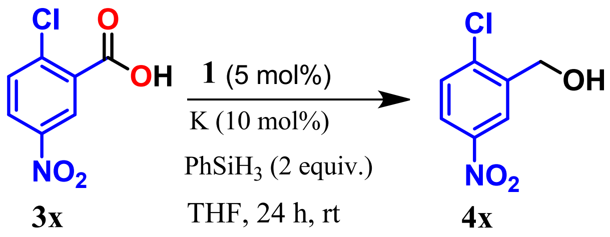

6. Solid state magnetic data of 2.

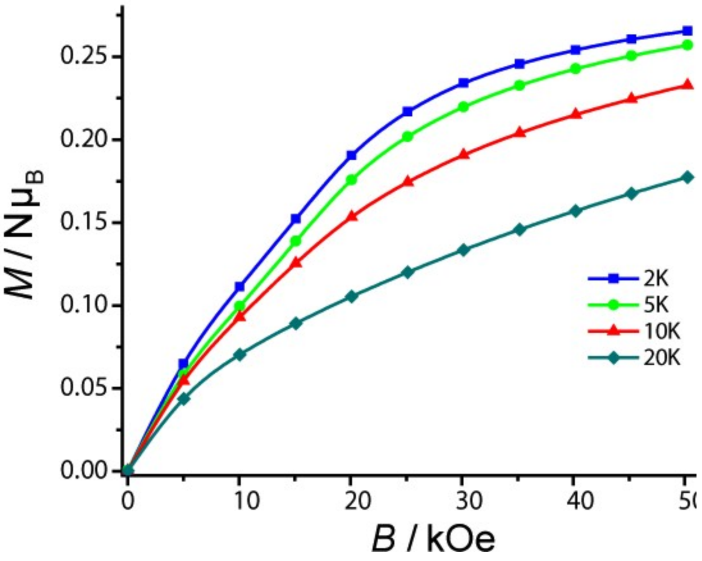

Figure S1. M/B plot for **2** at four different temperatures, exhibiting the magnetization is far below saturation at 5 Tesla field strength. The field dependent magnetization experiment shows the magnetization saturation at 2K as 1.2 $N\mu_B$, which is far lower than the Brillouin function value of 3 (for pure high spin system) and strongly supports the presence of large anisotropy in the system

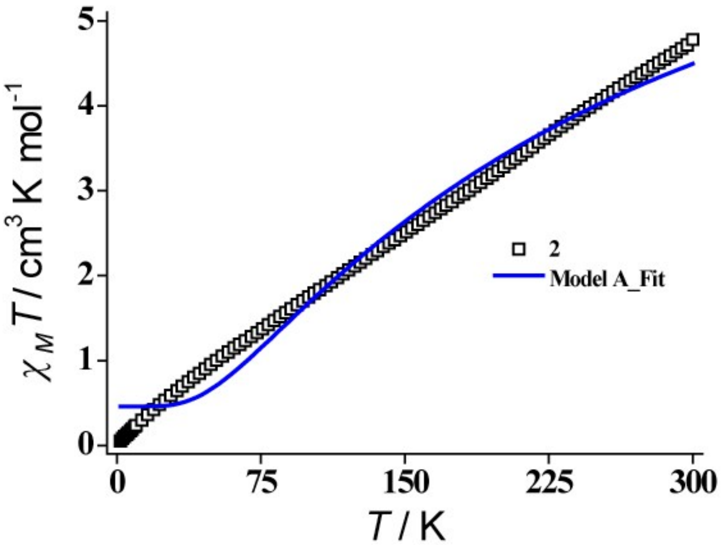

Figure S2. Experimental χ_mT data for **2** and its fit taking account of the model A. As can be seen, the fit is poor, thus indicating that model A (Figure 2d in main manuscript) is not the correct representation of the electronic structure in **2**.

Since we have proposed that a spin cross-over (SCO) is essential in model B (towards explaining the ground state of **2**) to reach LS-Co(II) (S=1/2), we sought for experimental evidence for the SCO phenomenon. Following is the plot of temperature-dependent hysteresis.

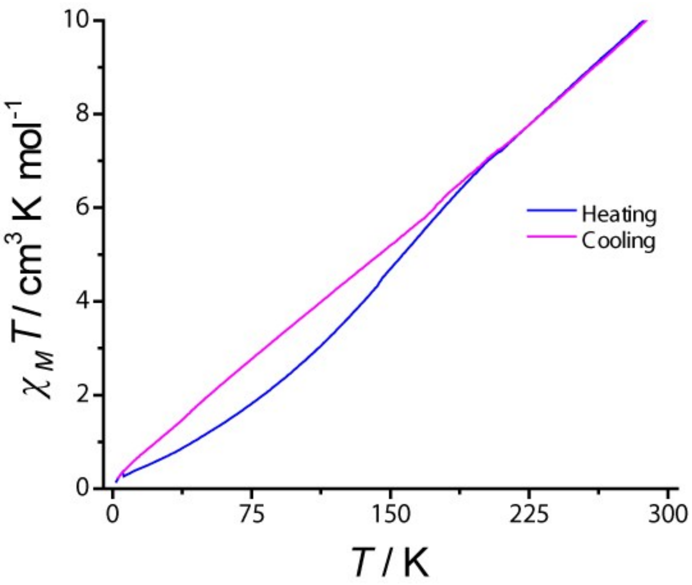

Figure S3. Temperature dependent hysteresis in **2** to prove that the spin crossover is happening on Co(II) center.

7. Detailed characterization of 1 and 1A

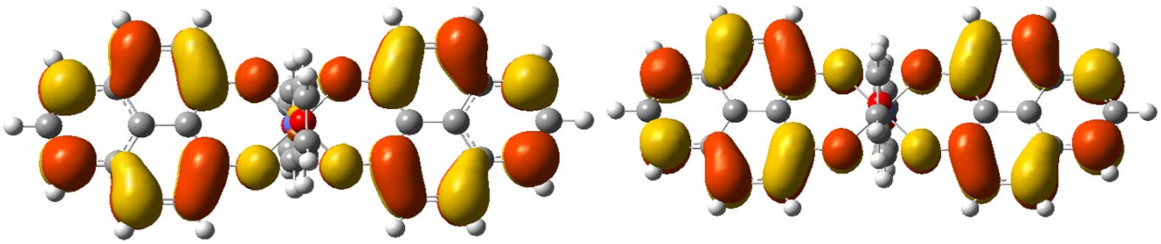

Figure S4. LUMO of **1** (left) and **1A** (right). The isosurface value is set to 0.02 (**e.**bohr^{-3})$^{1/2}$ for depiction.

8. **C-H….π interaction in complex 1**.

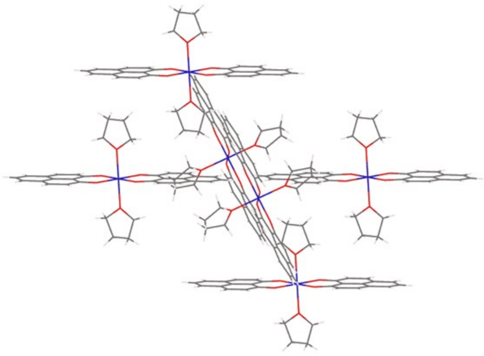

Figure S5. Packing diagram for **1**, where the C–H of an axial THF ligand is interacting with the π-ring of the PLY. This C–H(axial THF)…π(PLY ring) interaction is responsible partly for the gradual decrease of susceptibility via intermolecular AF-coupling at low temperature regime. This is also further evidenced by the modeling, giving a zJ' value of – 0.22 cm^{-1}.

9. **Control experiments for mechanistic investigation.**

To proof the mechanistic course for the hydrosilylation reaction of benzoic acid, we performed several stoichiometric reactions.

9a. Investigation into the radical nature of 1 catalyzed acid reduction.

To evaluate whether the hydrosilylation reaction of benzoic acid proceeded through a radical pathway, we performed the reaction in presence of a radical scavenger (TEMPO). An oven dried reaction tube was charged with **1** (14.83 mg, 0.025 mmol) and K (1.95 mg, 0.05 mmol) in 1.0 mL THF (1.0 mL) and the reaction mixture was stirred at room temperature for 4 h. Then $PhSiH_3$ (123.3 μL mmol), benzoic acid (61 mg, 0.5 mmol) and TEMPO (0.5/1 mmol) were added to the reaction mixture and stirred at room temperature for 24 h. Next, the reaction mixture was hydrolyzed by the drop-wise addition of 1.5 mL 2 (M) NaOH solutions and stirred at room temperature for another 3 h and the reaction mixture was diluted with water and the aqueous phase was extracted with Et_2O (15 mL). The organic layer was dried over $MgSO_4$. The solvent was removed under reduced pressure and characterized through ^1H NMR in $CDCl_3$.

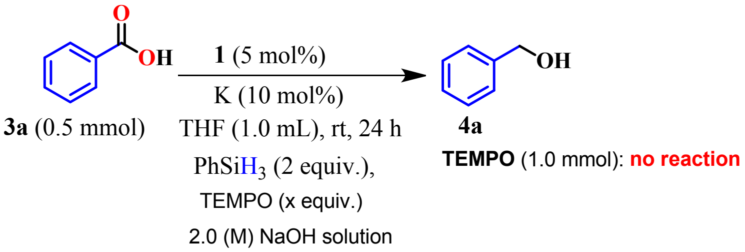

Scheme S2. Inhibition of **1** catalyzed acid reduction in presence of TEMPO.

9b. Radical trapping experiment through TEMPO adducts formation.

After confirming the radical nature of the hydrosilylation reaction, we probed Ph_2SiH_2 to trap the TEMPO adduct of Ph_2SiH_2. In an oven dried reaction tube **1** (59.3 mg, 0.1 mmol) and K (7.8 mg, 0.2 mmol) were taken followed by addition of 1.0 mL THF (1.0 mL) and the reaction mixture was stirred at room temperature for 4 h. Then Ph_2SiH_2 (18.6 µL, 0.1 mmol) was added to the reaction mixture at room temperature and stirred for 30 minutes. After that TEMPO (15.63 mg, 0.1 mmol) was added to it and stirred for another 6 h and the reaction mixture was subjected to HRMS characterization in acetonitrile followed by earlier report.[S17, S18]

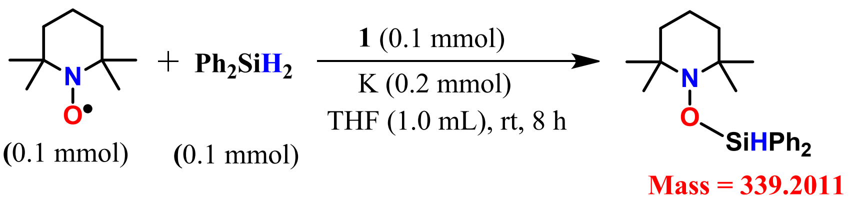

Scheme S3. TEMPO adduct of Ph_2Si radical.

9c. Synthesis of PLY Dimer (5).

To assess whether the hydrogen atom is stored in the redox active ligand (PLY) backbone, we have performed the stoichiometric reaction of reduced cobalt complex **2** (201.3 mg, 0.3 mmol) and $PhSiH_3$ (74 µL, 0.6 mmol) in THF (1.0 mL) at room temperature for 10 h. 1.0 mL of 12(M) aqueous HCl was added afterwards into the reaction mixture and stirred at room temperature for 1 h and the organic product was extracted in Et_2O. The PLY-dimer (**5**) was isolated through column chromatography using hexane: EtOAc mixture as eluent (4:1). Upon successful isolation, PLY-dimer was crystallized in concentrated $CHCl_3$ and characterized through SCXRD, as well as ^1H, ^{13}C, DEPT-135 and HMQC NMR spectroscopies.

^1H NMR (500 MHz, $CDCl_3$): δ 12.71 (s, 1H), 7.64 (d, J = 9.0 Hz, 1H), 7.45 (d, J = 8.0 Hz, 1H), 7.08 (t, J = 8.0 Hz, 1H), 6.94 (d, J = 8.0 Hz, 1H), 6.87 (d, J = 9.0 Hz, 1H), 3.65 – 3.64 (m, 1H), 3.20 – 3.10 (m, 2H) ppm.

$^{13}C\{^1H\}$ NMR (125 MHz, $CDCl_3$): δ 202.1, 161.9, 137.2, 130.9, 129.9, 129.1, 127.7, 126.4, 123.3, 118.7, 109.3, 45.2, 41.6 ppm. DEPT-135 NMR (125 MHz, $CDCl_3$): δ 137.2, 129.1, 127.7, 123.3, 118.7, 45.2, 41.6 ppm. ESI-MS: m/z calc. for $C_{26}H_{18}O_4H$ $[M+H]^+$ 395.1283, found 395.1287.

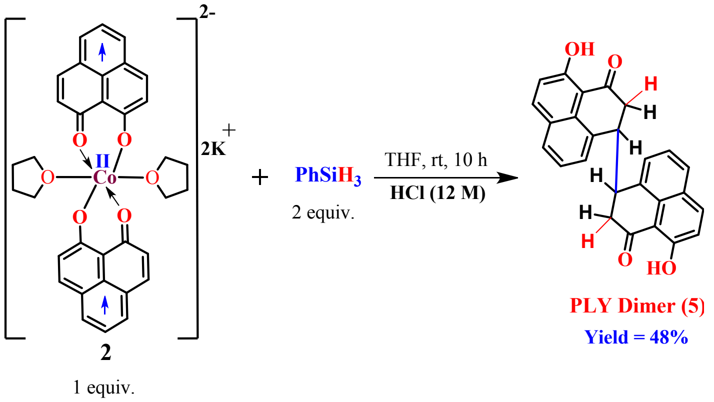

Scheme S4. Synthesis of PLY dimer, **5**.

9d. Mechanism of formation of PLY dimer.

To understand whether the redox equivalent is stored into the PLY (redox active) ligand in the form of C–H bond, we have performed the stoichiometric reaction of **2** (67.1 mg, 0.1 mmol) and $PhSiH_3$ (24.7 µL, 0.2 mmol) in 0.6 mL THF-d_8 and stirred for 10 h inside screw cap vial at room temperature inside the N_2 filled glove box and transferred the mixture to screw cap NMR tube. Then the NMR tube was taken out and 0.1 mL aqueous HCl [12(M)] was added to the reaction mixture and when an effervescence was observed. Immediately, ^1H NMR spectrum of the reaction mixture was recorded and a peak at δ 4.56 ppm was assigned to the H_2 gas in THF-d_8. Once the 1H NMR spectrum was recorded after opening the NMR tube cap, the peak at δ 4.56 ppm was vanished and it was confirmed further by checking ^1H NMR. Based on this observation as well as taking into consideration all analytical data including single crystal X-ray data, the plausible mechanism for the formation of PLY-dimer was drawn below.

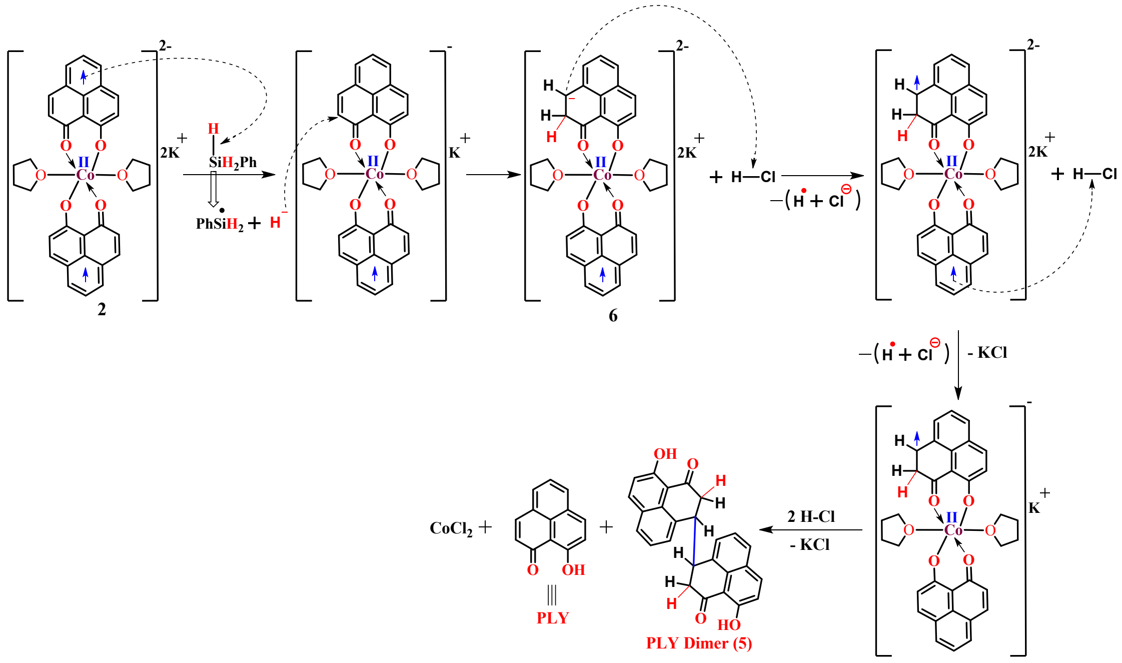

Scheme S5. Plausible mechanism for the formation of PLY-dimer, **5**.

9e. Synthesis of PLY Dimer-D_2 (5_D).

When complex **2** was reacted with $PhSiH_3$ in THF, hydrogen atom from $PhSiH_3$ was stored in redox active ligand backbone which was crystallographically chacterized. To prove this further by deuterium labelling experiment, stoichiometric reaction of **2** (201.3 mg, 0.3 mmol) and $PhSiD_3$ (76 µL, 0.6 mmol) in THF (1.0 mL) at room temperature for 10 h was conducted. After this 1.0 mL of 12(M) aqueous HCl was added into the reaction mixture and stirred at room temperature for 1 h. The organic product was extracted in Et_2O. The PLY-dimer-D_2 (**5**$_D$) was isolated through column chromatography using hexane: EtOAc mixture as eluent (20:1). Upon successful isolation, PLY Dimer-D_2 was characterized through ^1H, ^{13}C, DEPT-135, ^2H (Deuterium NMR) NMR spectroscopies and HRMS spectrometry.

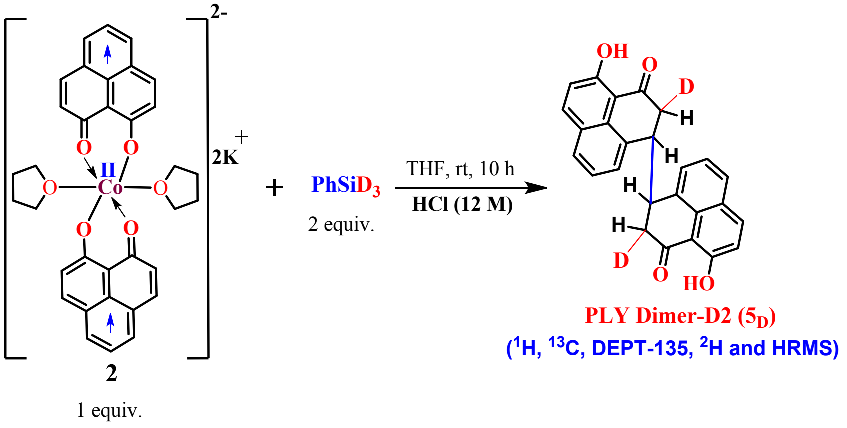

Scheme S6. Synthesis of PLY Dimer-D2 (**5**$_D$) from **2** using $PhSiD_3$.

^1H NMR (500 MHz, $CDCl_3$): δ 13.09 (s, 1H), 7.97 (d, J = 9.0 Hz, 1H), 7.66 (d, J = 8.5 Hz, 1H), 7.41 (d, J = 7.0 Hz, 1H), 7.33 (t, J = 8.0 Hz, 1H), 7.15 (d, J = 9.0 Hz, 1H), 3.32 (m, 1H), 2.95 (m, 1.70 H) ppm.

$^{13}C\{^1H\}$ NMR (125 MHz, $CDCl_3$): δ 203.8, 162.5, 138.1, 131.6, 130.6, 127.1, 126.7, 126.6, 123.9, 119.3, 110.2, 36.9, 27.3 (t, $^1J_{C\text{-}D}$ = 20 Hz) ppm.

DEPT-135 NMR (125 MHz, $CDCl_3$): δ 138.1, 126.7, 126.6, 124.0, 119.3, 37.0, 27.3 (t, $^1J_{C\text{-}D}$ = 20 Hz) ppm.

$^2H\{^1H\}$ NMR (61.4 MHz, CH_2Cl_2): 3.33 ppm.

^2H NMR (61.4 MHz, CH_2Cl_2): 3.32 (d, $^2J_{D\text{-}H}$ = 1.4 Hz) ppm.

HRMS: m/z calc. for $C_{26}H_{16}D_2O_4Na$ $[M+Na]^+$ 419.1228, found 419.1216.

9f. Synthesis and characterization of byproduct Silanol.

In an oven dried reaction tube, **2** (33.6 mg, 0.05 mmol) and $PhSiH_3$ (123.3 µL, 1.0 mmol) were charged followed by the addition of 1.0 mL THF and stirred it for 30 minutes at room temperature. Then benzoic acid (61.1 mg, 0.5 mmol) was added to the reaction mixture and stirred at room temperature for 18 h. Next the reaction mixture was dried in Schlenk line and extracted from dry hexane and removed the solvent in reduced pressure to afford the byproduct phenylsilanol which was further characterized through NMR spectroscopy.[S19, S20]

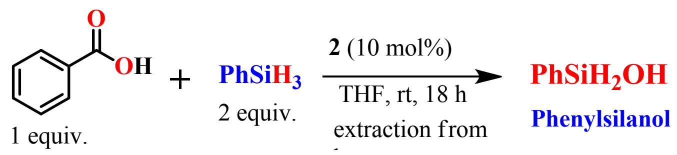

Scheme S7. Synthesis of byproduct phenylsilanol.

9g. Isolation of hydrosilylation product of benzoic acid.

To identify the benzoic acid and benzaldehyde hydrosilylation product, an oven dried reaction tube was charged with doubly reduced product **2** (33.6 mg, 0.05 mmol, 10 mol%) and $PhSiH_3$ (123.3 µL, 1.0 mmol) followed by the addition of 1.0 mL THF and the reaction mixture was stirred for 30 minutes at room temperature. Then benzoic acid (61.1 mg, 0.5 mmol) was added to the reaction mixture and stirred at room temperature for 18 h. Next the reaction mixture was dried in Schlenk line and washed with dry hexane and the residue was extracted with hexane: Et_2O (2:1). Then the solvent was removed in reduced pressure to afford the desired hydrosilylation product which was further characterized through mass spectrometry and NMR spectroscopy following an earlier report.[S21]

The same procedure was followed for the hydrosilylation of benzaldehyde.

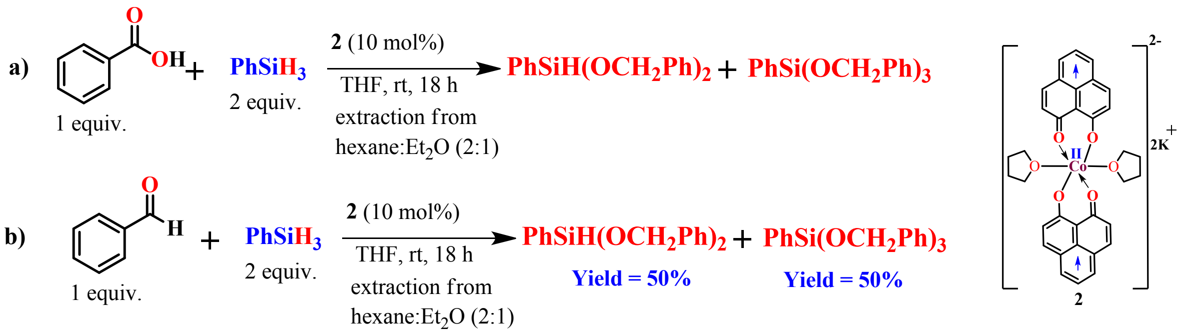

Scheme S8. Synthesis of hydrosilylation product, **9** from both benzoic acid and benzaldehyde.

9h. Method for Reduction of Benzoic Acid in presence of $PhSiD_3$.

Inside a N_2 filled glovebox, an oven dried reaction tube was charged with two electron reduced catalyst **2** (16.8 mg, 0.025 mmol, 5 mol%) and dry THF (1.5 mL) and then $PhSiD_3$ (126.7 µL, 1.0 mmol) was added to the reaction mixture followed by addition of the corresponding carboxylic acids (0.5 mmol). The reaction mixture was stirred at room temperature for another 20 h and after completion of the reaction, the hydrosilylated product was hydrolyzed by drop-wise addition of 1.5 mL 2 M NaOH solutions. Then, the aqueous phase was extracted with Et_2O/EtOAc (15 mL). The organic layer was washed with brine and dried over $MgSO_4$. The solvent was removed under reduced pressure and the desired product was purified by flash column chromatography on silica gel. Copious amount of proteo, duetereo scrambled benzyl alcohol (**4_D**) was obtained along with some proteo-only variety (**4**). **4** forms in the reaction mixture owing to spontaneous exchange of deuterium in $PhSiD_3$ with the solvent over time.

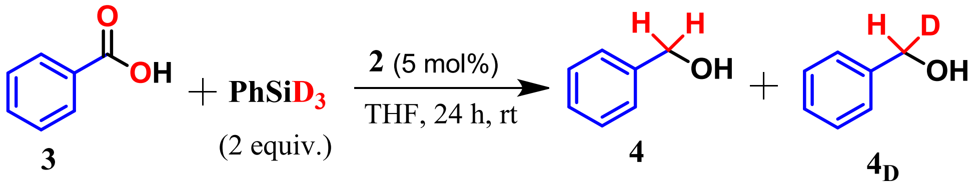

9i. Role of Reducing Agent K in Reduction of Benzoic Acid.

To investigate whether potassium is more intimately involved in the reaction mechanism, we have performed a cation exchange reaction. Accordingly, **2** was treated with large excess (~10 equivalent) of sodium pivalate (50 mol%) and stirred for 24 hours to ensure that the potassium is getting replaced with sodium. The modified catalyst is equally efficient in hydrosilylating carboxylic acids in similar yield, 86%.

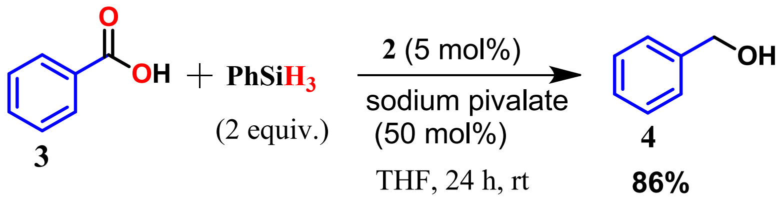

Control experiment to prove that sodium-based reaction failed due to kinetic inhibition

We hypothesize that the failure of sodium-based reduction stems from the kinetic reason although thermodynamically it is capable of reducing **1** given its reduction potential. To prove this, **1** was sonicated with 2 equivalent of metallic sodium for 4 hours and the resulting reduced complex was utilized for the reduction of benzoic acid. Under the same reaction conditions benzyl alcohol was formed in 34% yield.

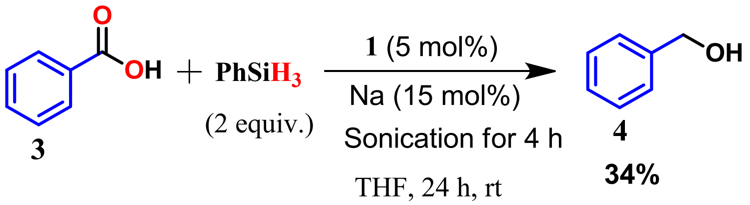

10. **X-ray crystallographic details**.

Single crystals of compounds **1** and **5** were mounted on a glass pip. Intensity data were collected on a SuperNova, Dual, Mo at zero, Eos diffractometer. The crystals were kept at 100K during data collection. Atomic coordinates, isotropic and anisotropic displacement parameters of all the non-hydrogen atoms of two compounds were refined using Olex2,[S22] and the structure was solved with the Superflip[S23] structure solution program using Charge Flipping and refined with the ShelXL[S24]refinement package using Least Squares minimization. Structure graphics shown in the figures were created using the Olex2 and X-Seed software package version 2.0.[S25]

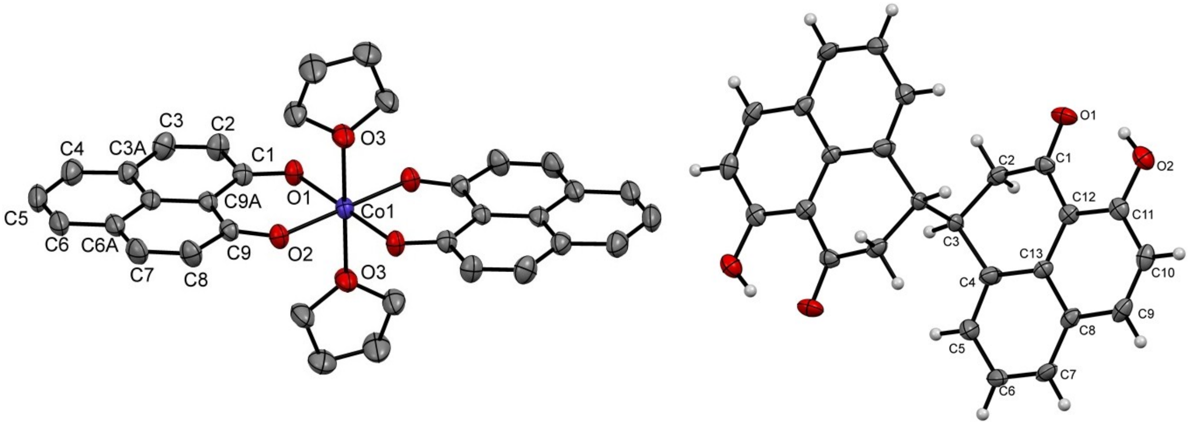

Figure S6. Views of the molecular structures of **1** (left) and **5** (right). Ellipsoids are set at 50% probability level; hydrogen atoms of **1** have been omitted for the sake of clarity.

Table S2. Crystallographic and structure refinement data for complexes **1** and **5**

Complex	**1**	**5**
CCDC No	1828004	1846773
Formula	$C_{34}\,H_{30}\,O_6\,Co$	$C_{26}\,H_{18}\,O_4$
Fw	593.51	393.69
Crystal System	Monoclinic	orthorhombic
Space group	*P 21/n*	*P c c n*
a [Å]	9.6119(5)	7.3790(5)
b [Å]	16.0078(7)	14.8543(10)
c [Å]	12.0749(7)	16.8845(10)

α [^{0}]	90	90.00
β [^{0}]	94.068(6)	90.00
γ [^{0}]	90	90.00
V [Å ^{3}]	1853.23(17)	1850.7(2)
Z	2	4
λ [Å]	0.71073	0.71073
ρ_{calcd} [gcm^{-3}]	1.0636	1.413
F[000]	615.0820	821
μ [mm^{-1}]	3.918	0.095
θ [^{0}]	5.38-66.32	2.41-25.03
index ranges	$-9\leq h \leq 11$<br>$-18\leq k \leq 15$<br>$-14\leq l \leq 14$	$-8\leq h \leq 8$<br>$-17\leq k \leq 15$<br>$-19\leq l \leq 20$
T [K]	100	100
$R1$	0.0489	0.0430
$wR2$	0.1398	0.1040
R_{merge}	0.0608	0.0526
Parameters	188	147
GOF	0.9725	1.054
reflns total	7256	10323
unique reflns	3218	1635
Obsdreflns	2514	1372

Table S3.Selected bond distances (Å) and angles (°) observed in **1**

Bond	Distance	Bond	Angles
Co(1)-O(2)	2.0101(18)	O(2)-Co(1)-O(2)	180.0
Co(1)-O(1)	2.0092(19)	O(1)-Co(1)-O(2)	92.60(7)
Co(1)-O(3)	2.210(2)	O(1)-Co(1)-O(1)	180.0
O(2)-C(9)	1.276(3)	O(3)-Co(1)-O(2)	90.55(8)
O(1)-C(1)	1.271(3)	O(3)-Co(1)-O(1)	88.97(9)
C(9A)-C(9)	1.429(4)	O(3)-Co(1)-O(3)	180.0
C(9A)-C(1)	1.434(4)	C(9A)-C(9)-O(2)	125.2(2)
C(9)-C(8)	1.449(4)	C(8)-C(9)-O(2)	116.5(3)
C(1)-C(2)	1.438(4)	C(1)-C(9A)-C(9)	122.1(2)
		C(8)-C(9)-C(9A)	118.3(3)
		C(2)-C(1)-O(1)	116.9(3)
		C(2)-C(1)-C(9A)	118.2(2)

Table S4. Selected bond distances (Å) and angles (°) observed in **5**

Bond	Distance	Bond	Angles
O(2)-C(11)	1.339(2)	O(1)-C(1)-C(12)	121.64(17)
O(1)-C(1)	1.254(2)	O(2)-C(11)-C(12)	121.24(16)
C(2)-C(1)	1.488(3)	C(11)-C(12)-C(1)	120.28(16)
C(12)-C(1)	1.449(2)	O(1)-C(1)-C(2)	120.75(15)
C(12)-C(11)	1.392(2)	O(2)-C(11)-C(10)	118.35(17)
C(4)-C(5)	1.376(2)	C(1)-C(2)-C(3A)	113.30(15)

C(6)-C(5)	1.397(2)	C(3B)-C(2)-C(1)	117.5(6)
C(2)-C(3A)	1.553(4)	C(2)-C(3A)-C(3A)	111.0(2)
C(2)-C(3B)	1.198(12)	C(4)-C(3A)-C(3A)	112.7(3)
C(4)-C(3A)	1.530(3)	C(5)-C(4)-C(3A)	121.15(18)
C(4)-C(3B)	1.547(12)	C(5)-C(4)-C(3B)	125.1(6)
C(3A)-C(3A)	1.571(8)	C(4)-C(5)-C(6)	121.12(18)
		C(7)-C(6)-C(5)	120.38(17)

11. NMR data of benzyl alcohol derivatives after reduction of various carboxylic acids.

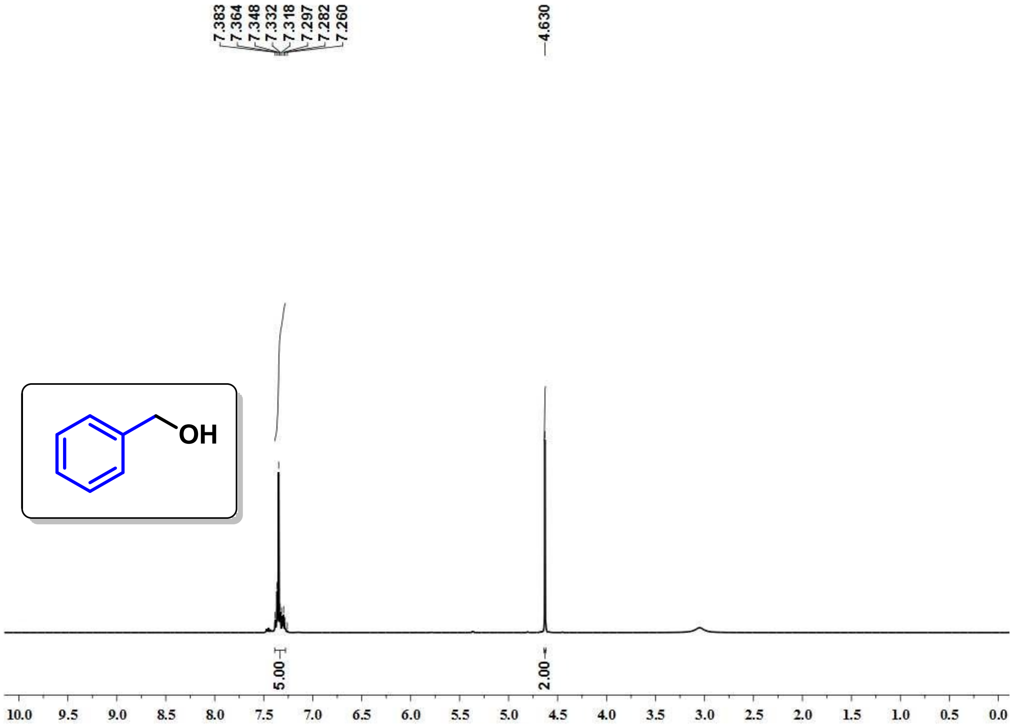

Figure S7. ^{1}H NMR spectrum of benzyl alcohol, **4a** recorded in $CDCl_3$.

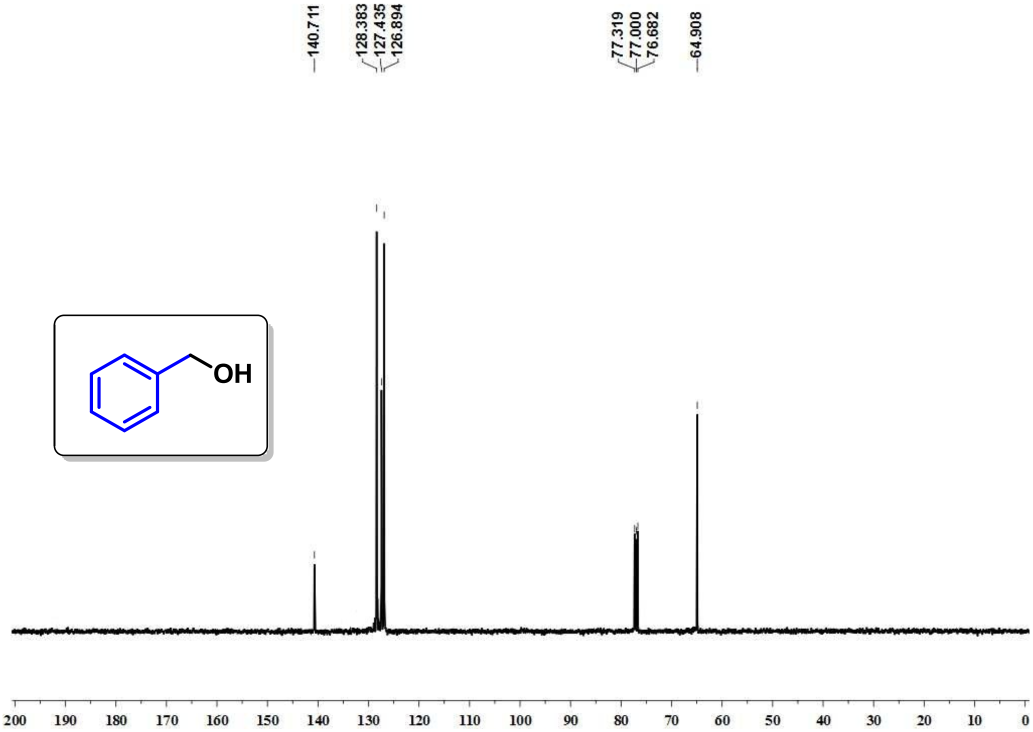

Figure S8. ^{13}C NMR spectrum of benzyl alcohol, **4a** recorded in $CDCl_3$.

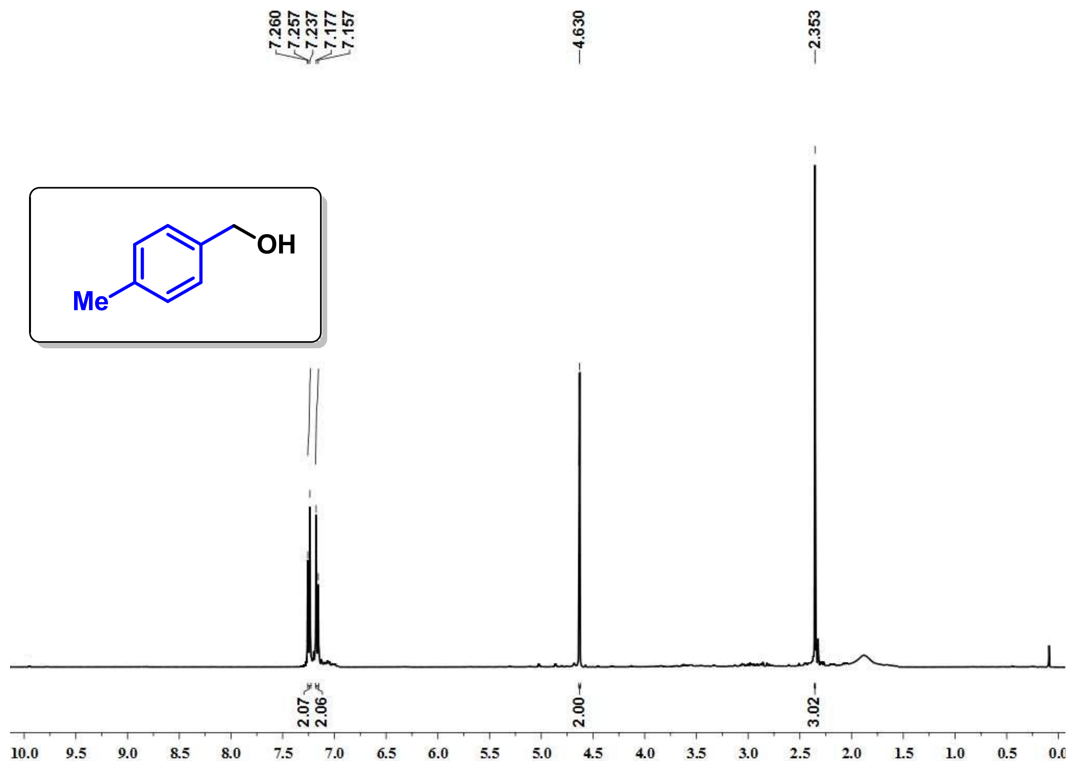

Figure S9. ^{1}H NMR spectrum of 4-methylbenzyl alcohol, **4b** recorded in $CDCl_3$.

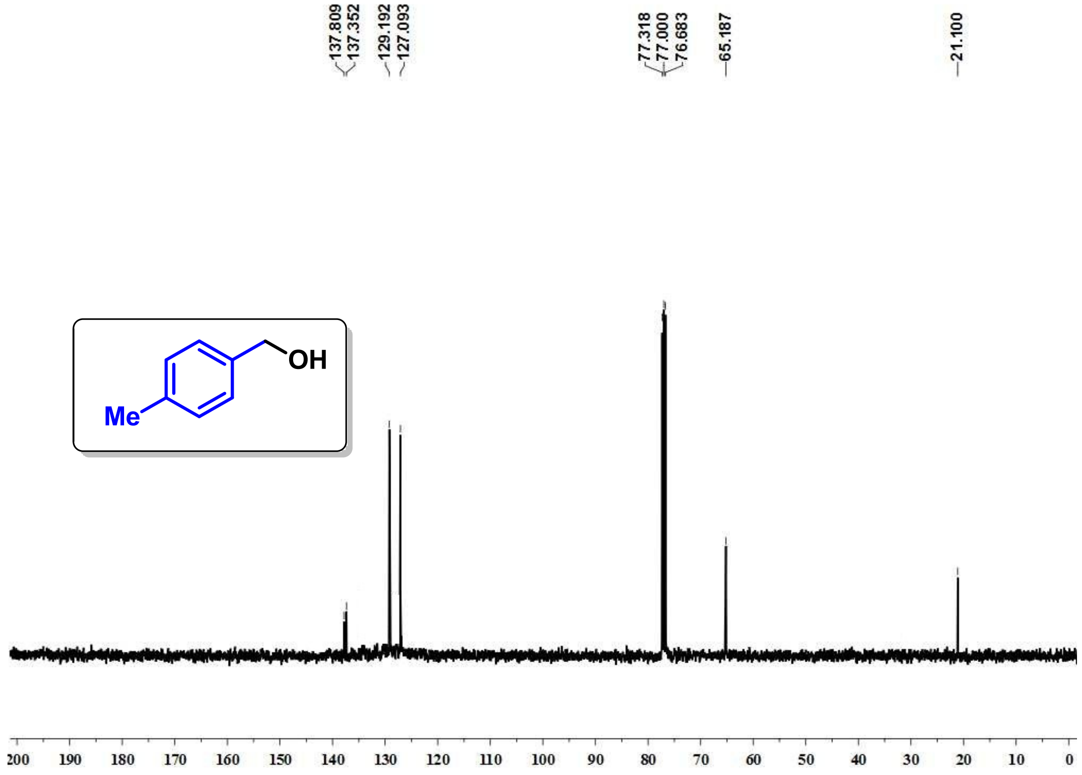

Figure S10. ^{13}C NMR spectrum of 4-methylbenzyl alcohol, **4b** recorded in $CDCl_3$.

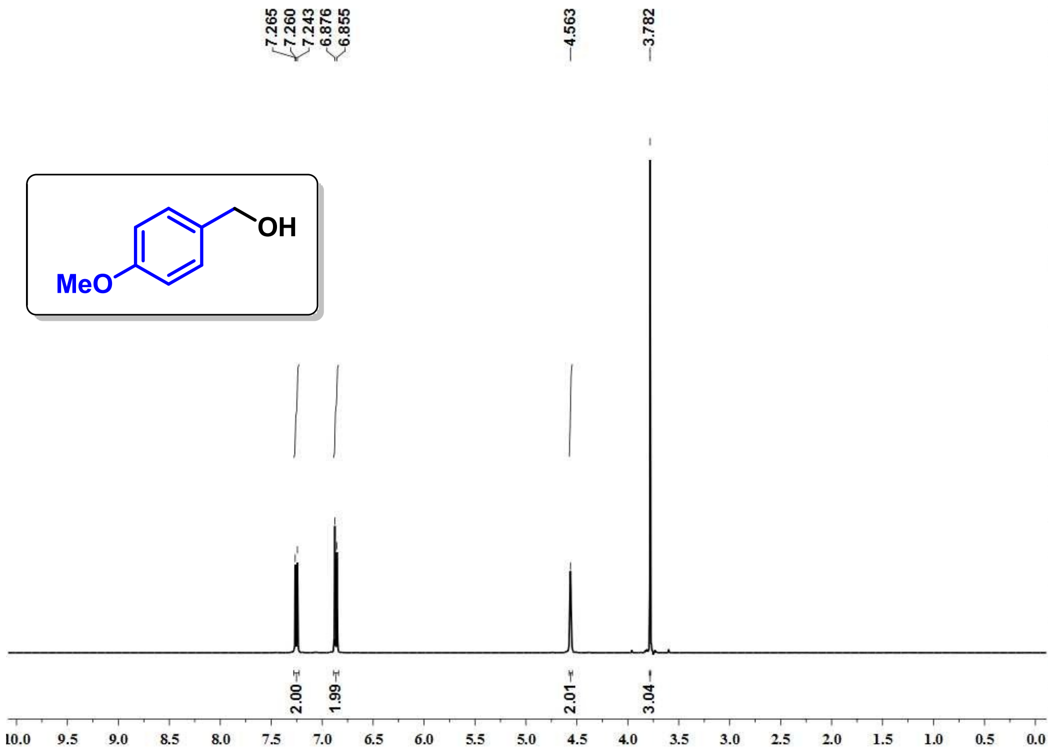

Figure S11. ^{1}H NMR of 4-methoxybenzyl alcohol**, 4c** recorded in $CDCl_3$.

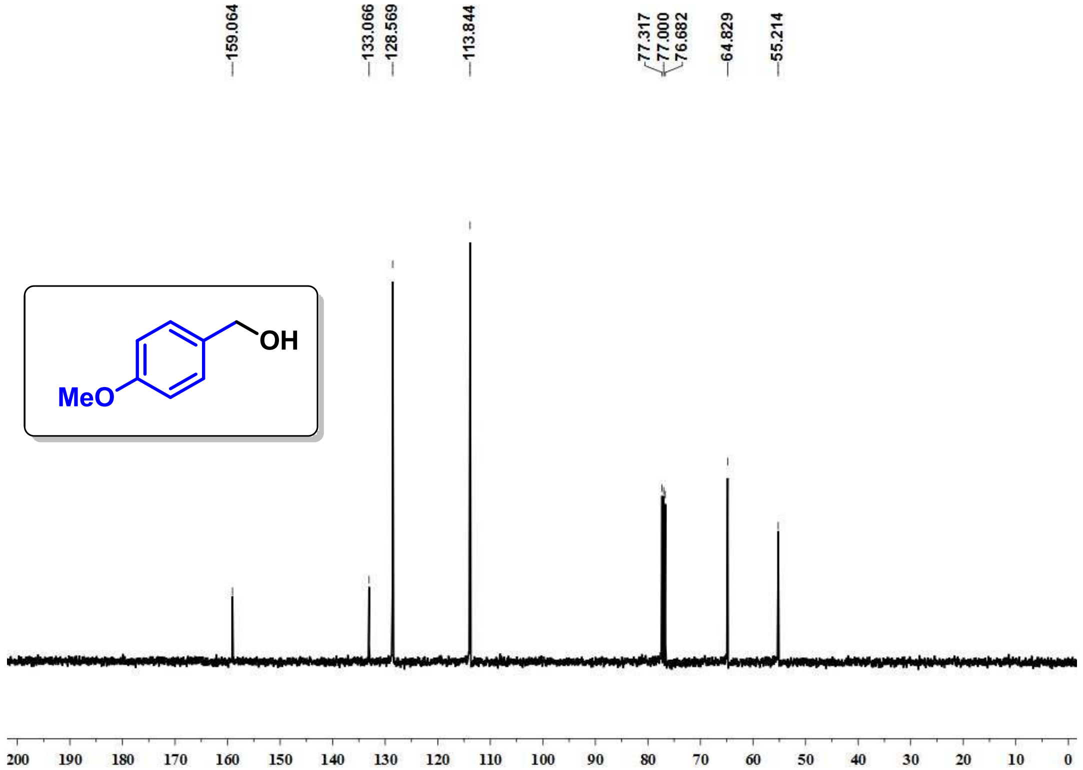

Figure S12. ^{13}C NMR spectrum of 4-methoxybenzyl alcohol**, 4c** recorded in $CDCl_3$.

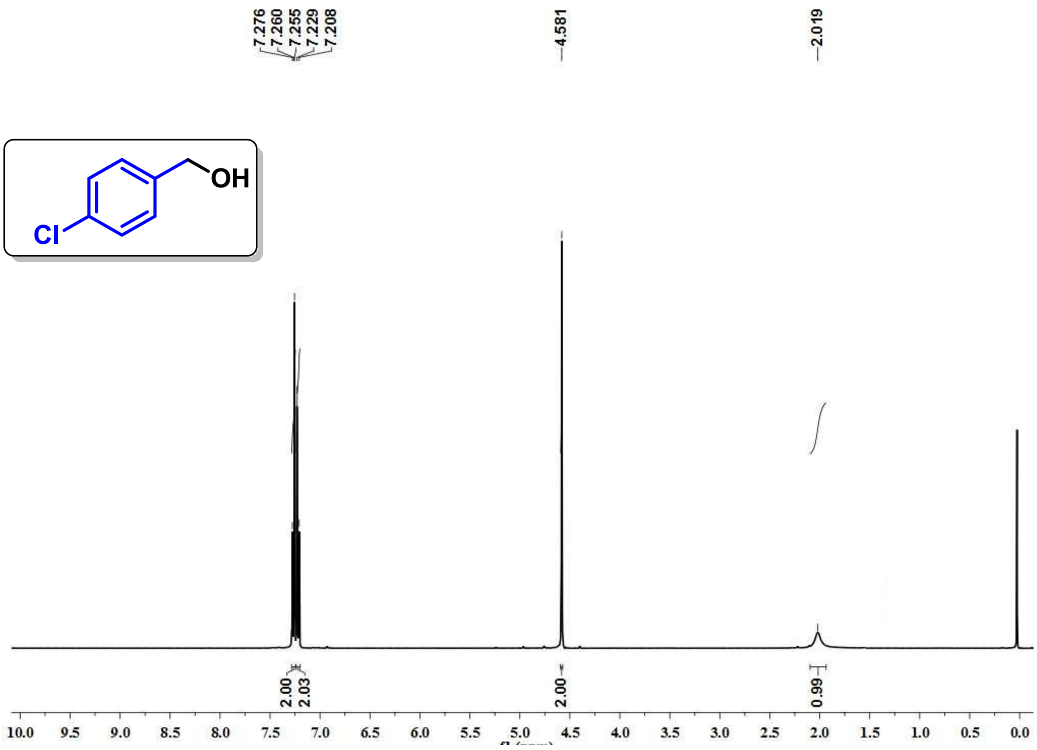

Figure S13. ^{1}H NMR spectrum of 4-chlorobenzyl alcohol, **4d** recorded in $CDCl_3$.

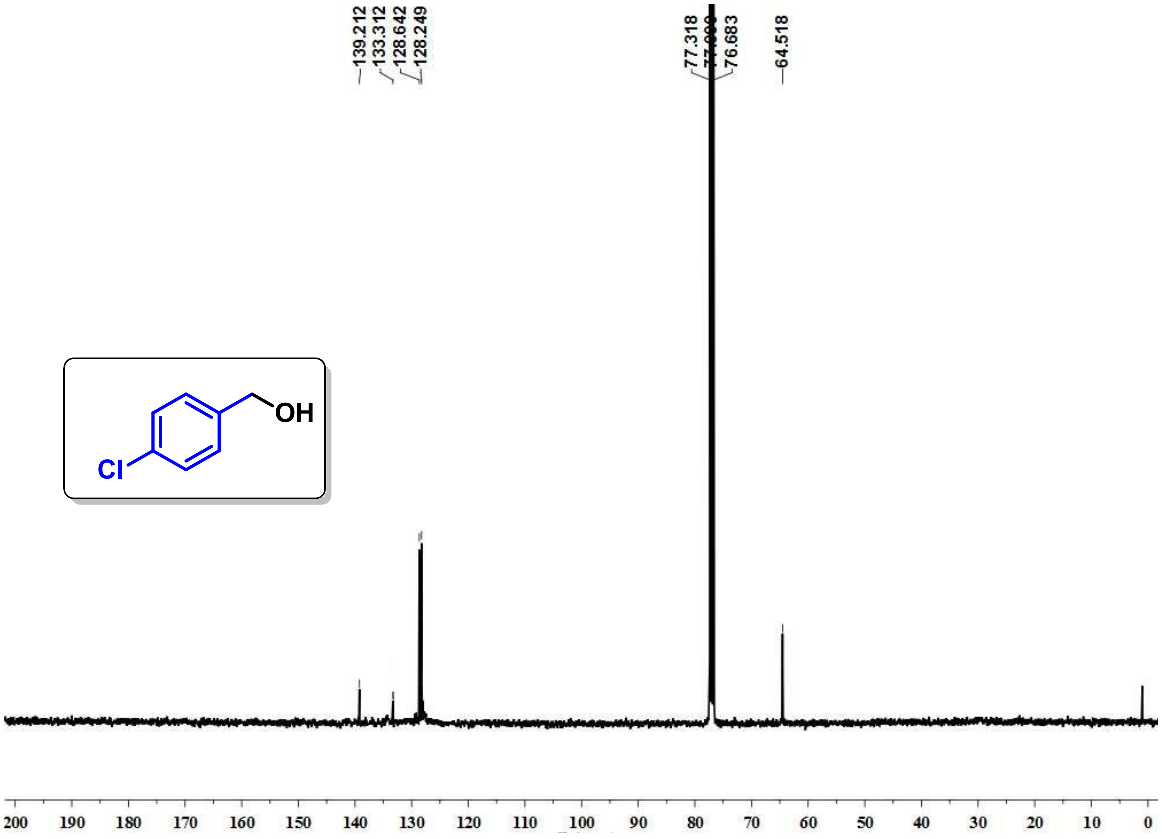

Figure S14. ^{13}C NMR spectrum of 4-chlorobenzyl alcohol, **4d** recorded in $CDCl_3$.

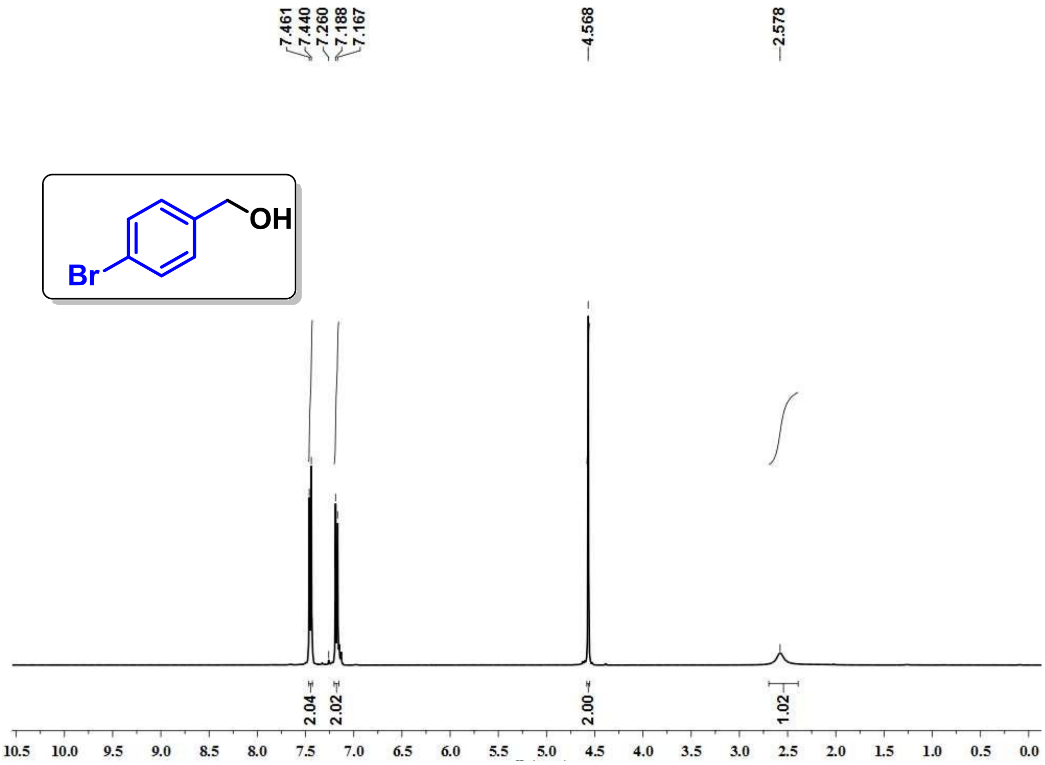

Figure S15. ^{1}H NMR spectrum of 4-bromobenzyl alcohol, **4e** recorded in $CDCl_3$.

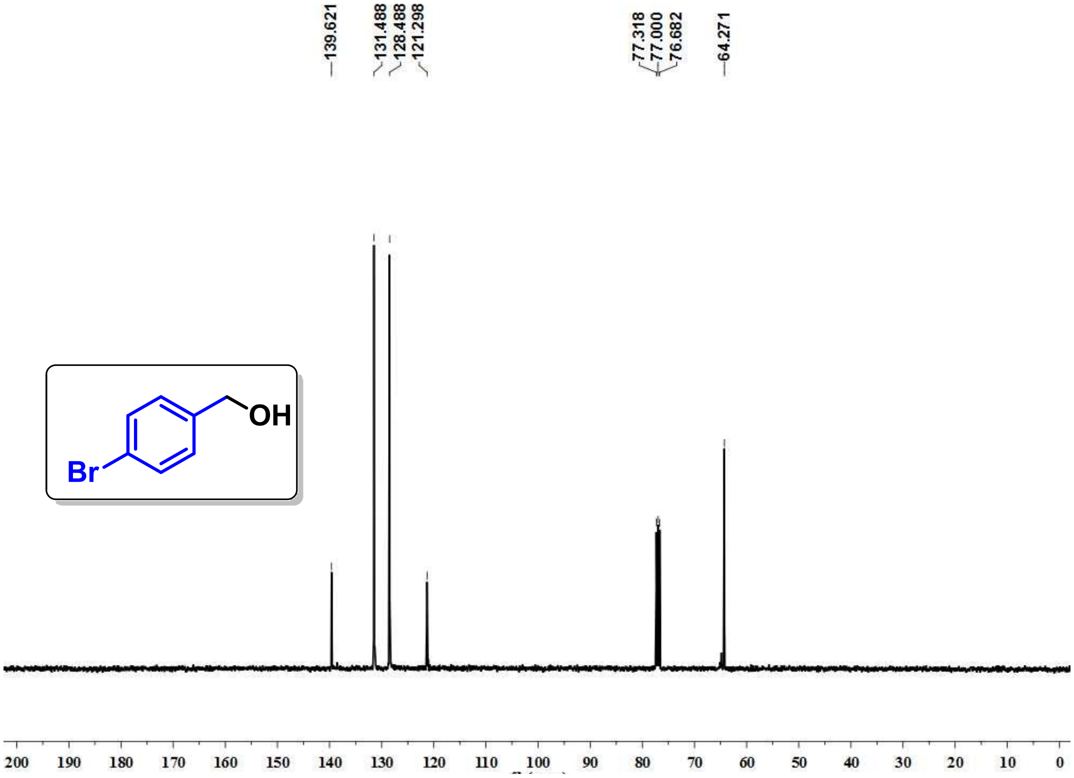

Figure S16. ^{13}C NMR spectrum of 4-bromobenzyl alcohol, **4e** recorded in $CDCl_3$.

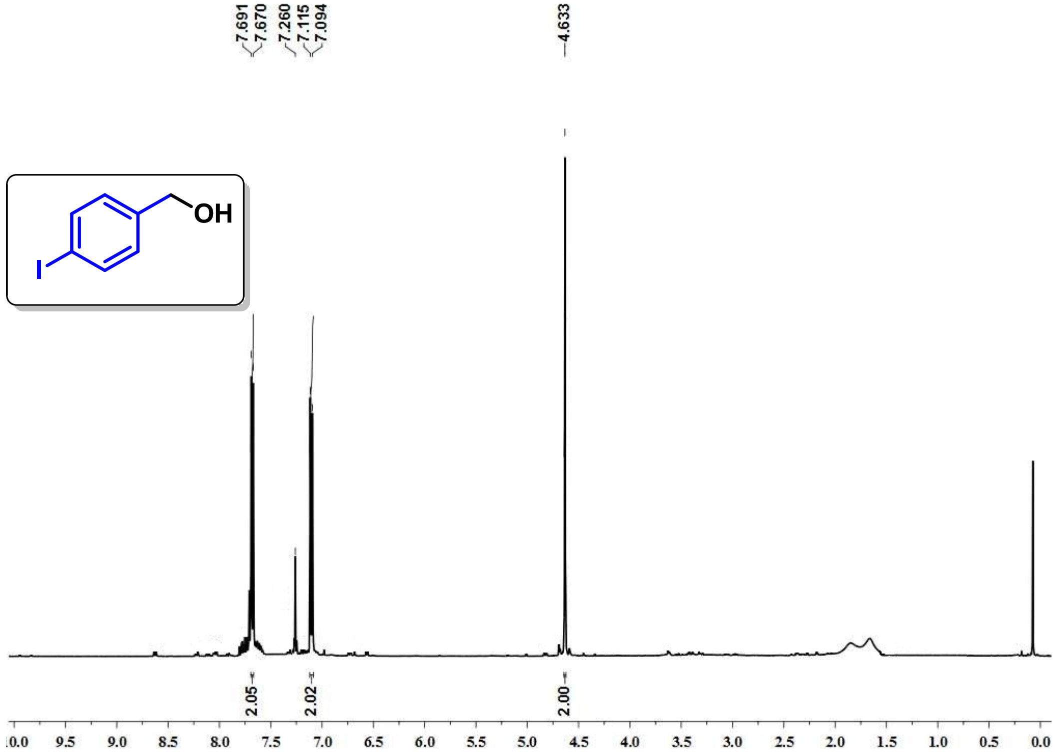

Figure S17. ^{1}H NMR spectrum of 4-iodobenzyl alcohol, **4f** recorded in $CDCl_3$.

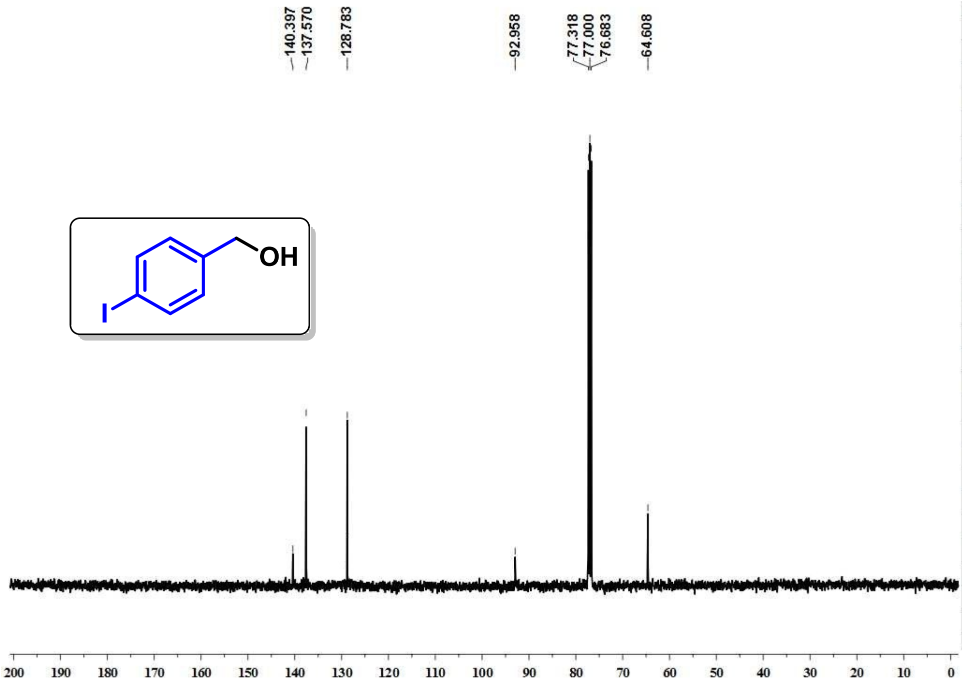

Figure S18. ^{13}C NMR spectrum of 4-iodobenzyl alcohol, **4f** recorded in $CDCl_3$.

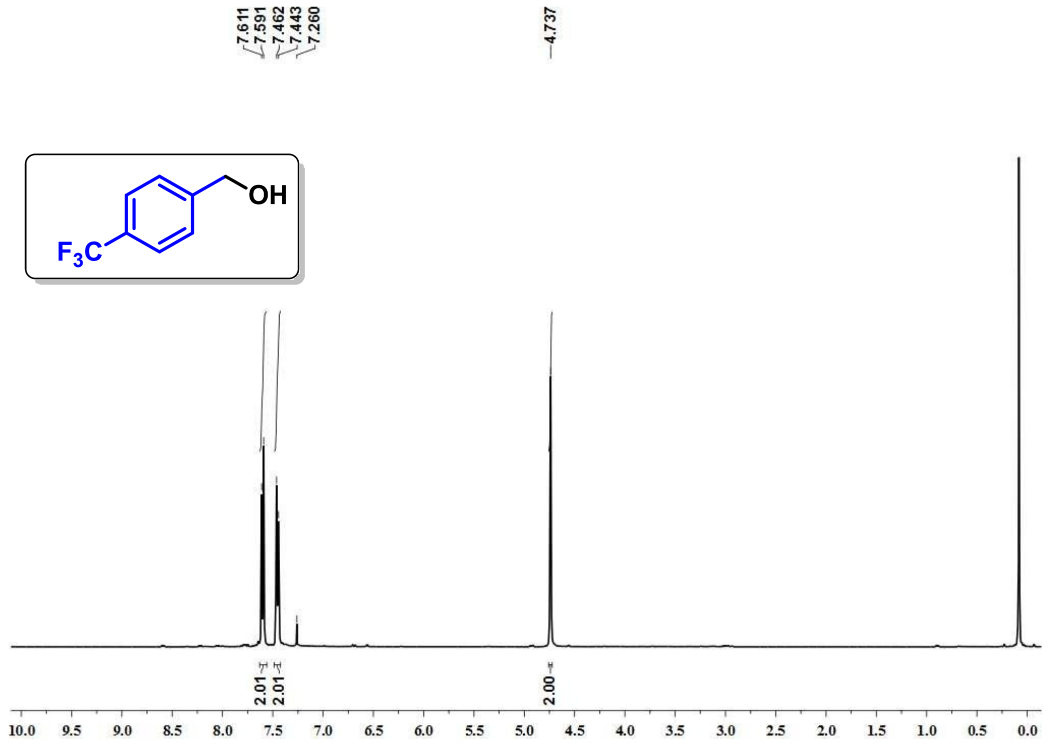

Figure S19. ^{1}H NMR spectrum of 4-(trifluoromethyl)benzyl alcohol, **4g** recorded in $CDCl_3$.

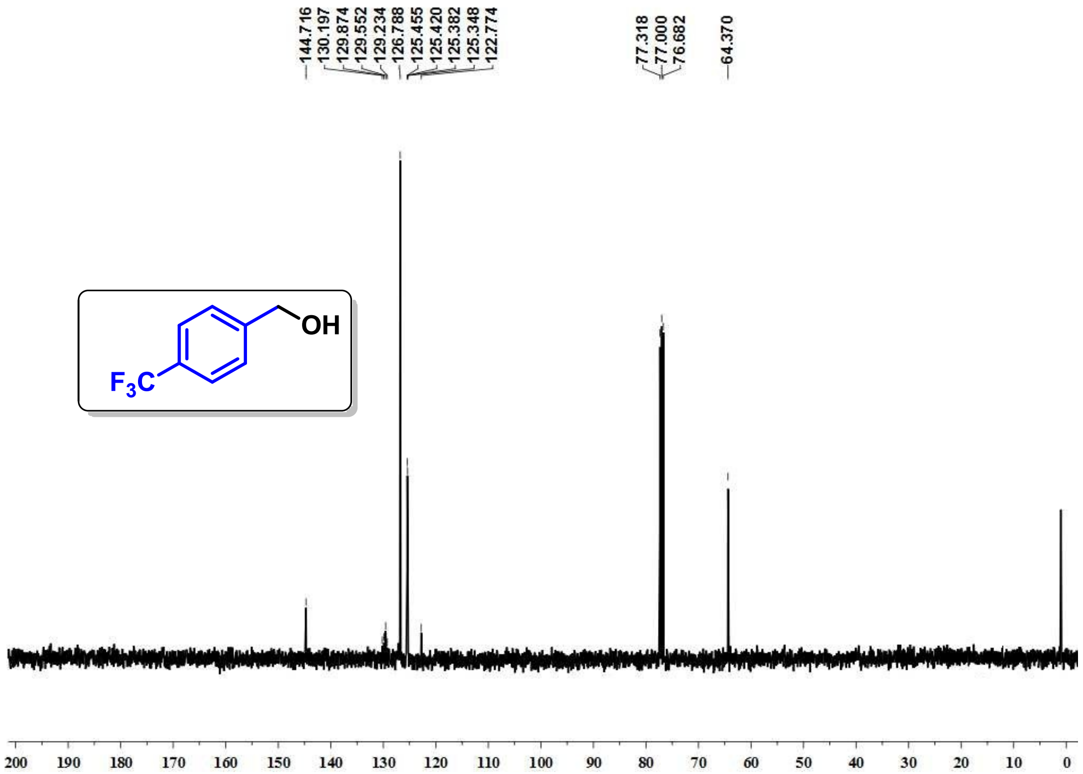

Figure S20. ^{13}C NMR spectrum of 4-(trifluoromethyl)benzyl alcohol, **4g** recorded in $CDCl_3$.

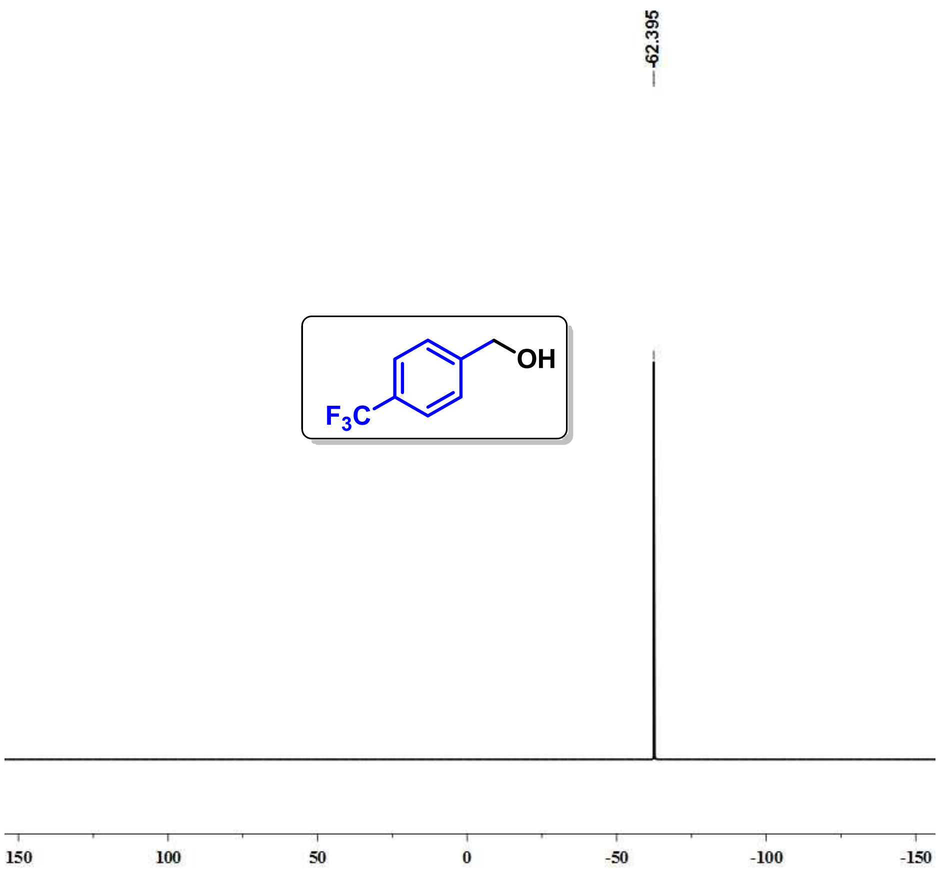

Figure S21. ^{19}F NMR spectrum of 4-(trifluoromethyl)benzyl alcohol, **4g** recorded in $CDCl_3$.

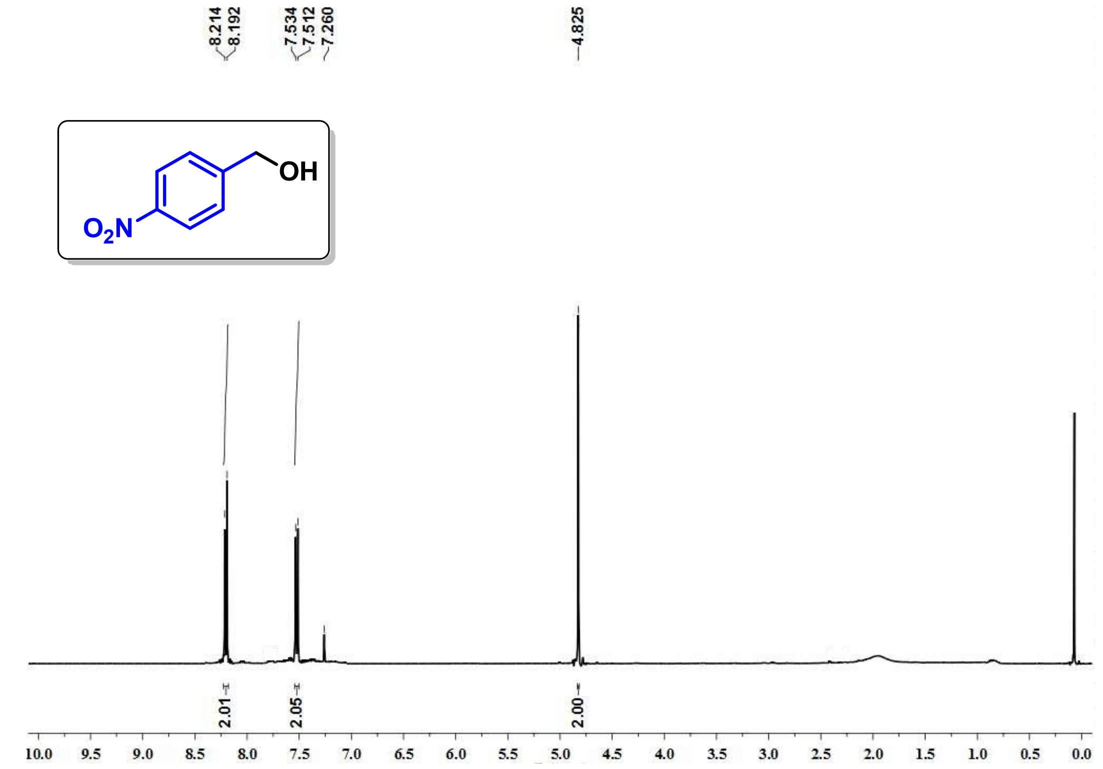

Figure S22. ^{1}H NMR spectrum of 4-nitrobenzyl alcohol, **4h** recorded in $CDCl_3$.

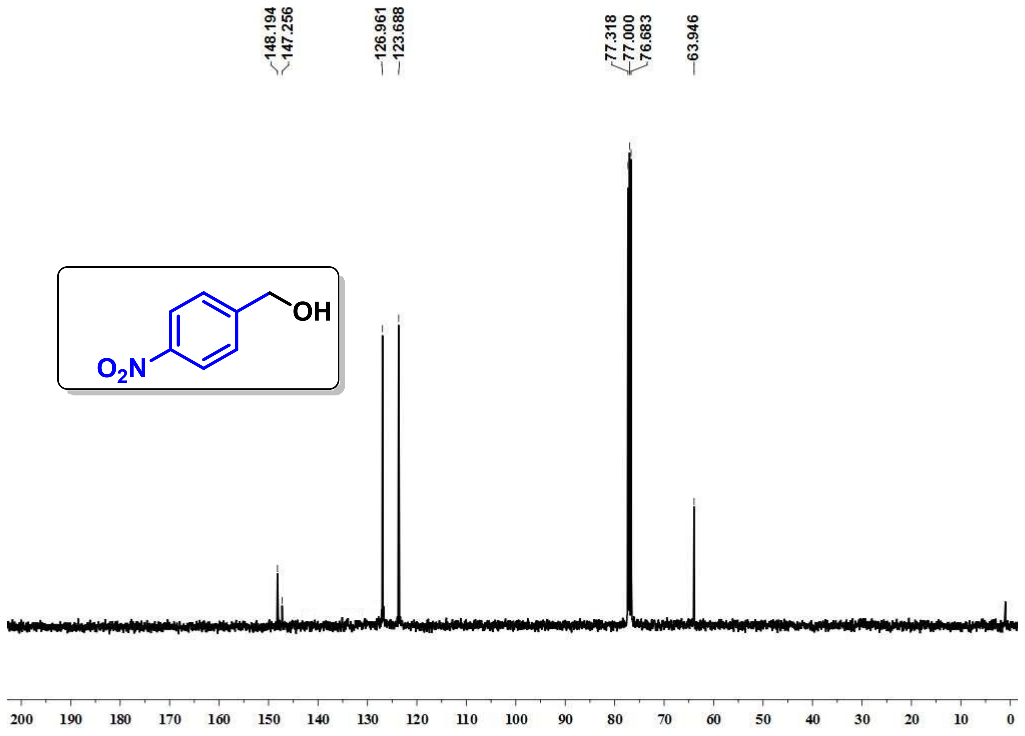

Figure S23. ^{13}C NMR spectrum of 4-nitrobenzyl alcohol, **4h** recorded in $CDCl_3$.

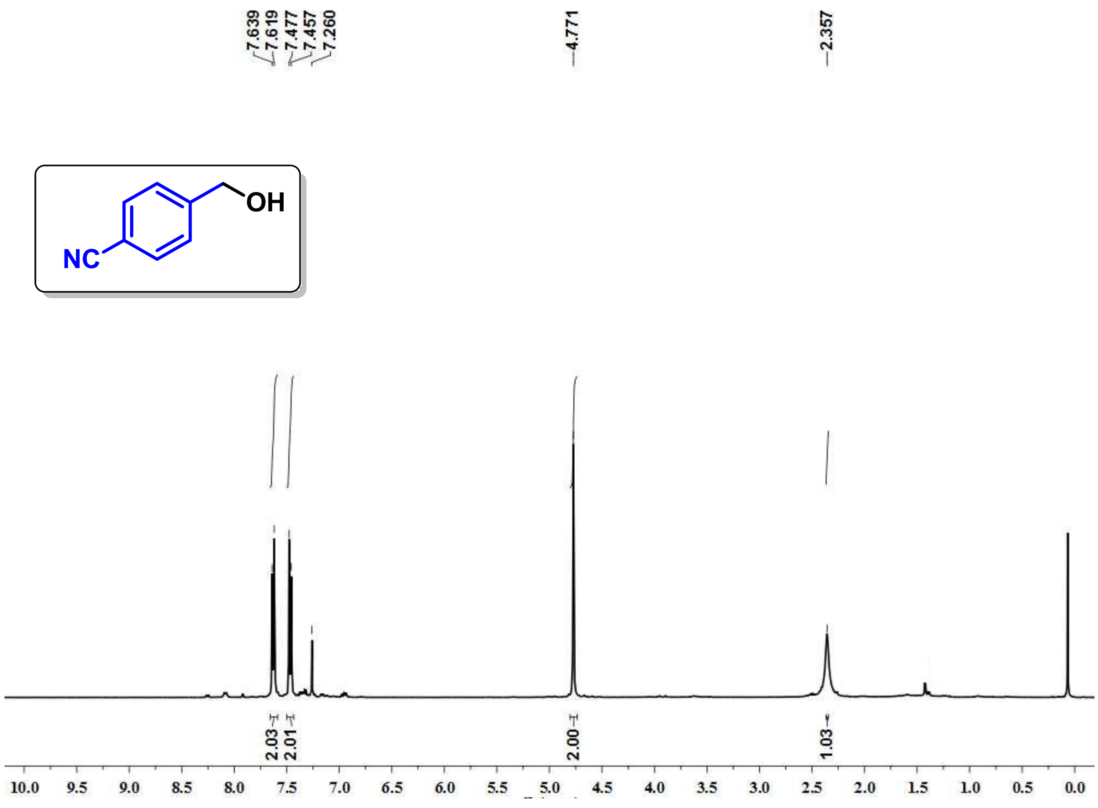

Figure S24. ^{1}H NMR spectrum of 4-cyanobenzyl alcohol, **4i** recorded in $CDCl_3$.

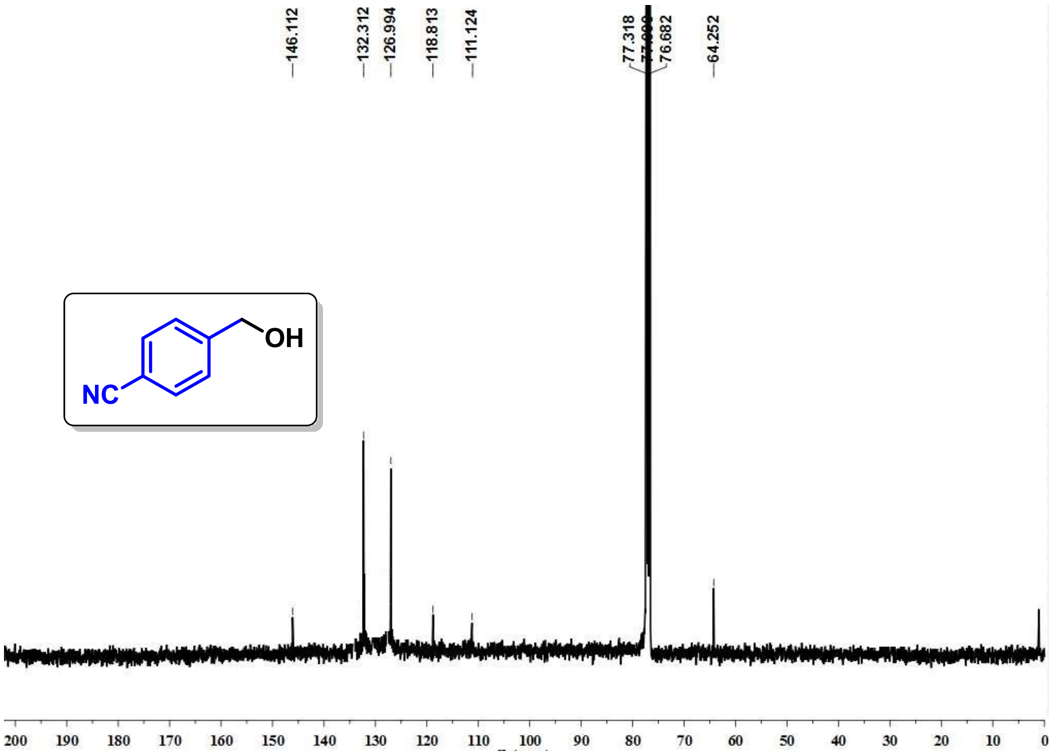

Figure S25. ^{13}C NMR spectrum of 4-cyanobenzyl alcohol, **4i** recorded in $CDCl_3$.

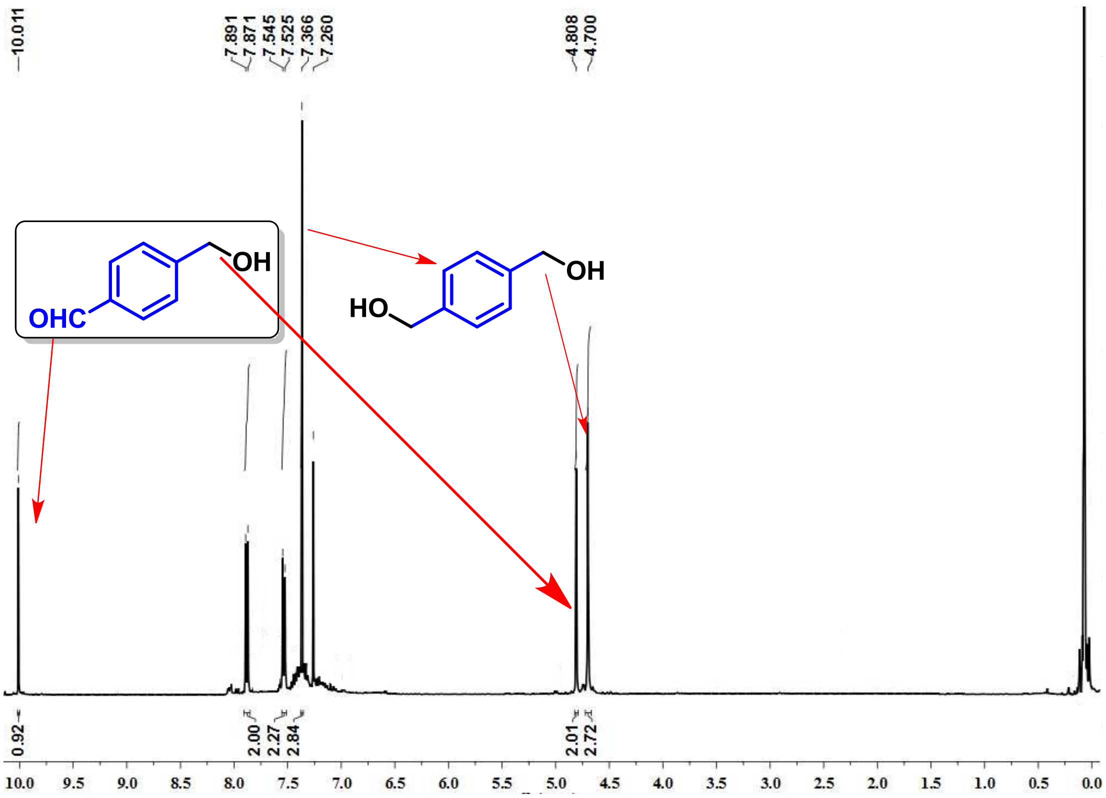

Figure S26. ^{1}H NMR spectrum of mixture of 4-(hydroxymethyl)benzaldehyde / 1,4-phenylenedimethanol (2/1), **4j** recorded in $CDCl_3$.

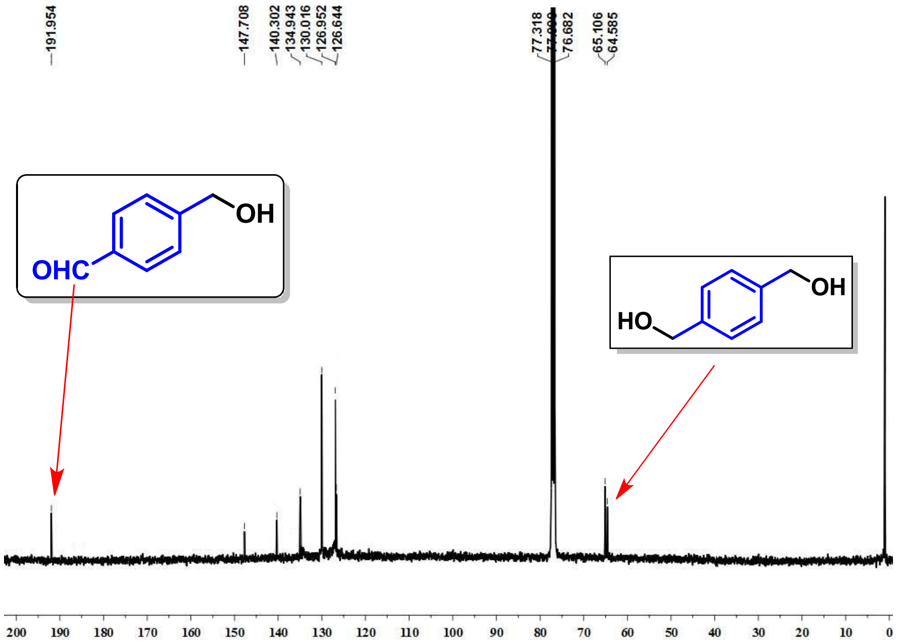

Figure S27. ^{13}C NMR of mixture of 4-(hydroxymethyl)benzaldehyde / 1,4-phenylenedimethanol (2/1)**, 4j** in $CDCl_3$.

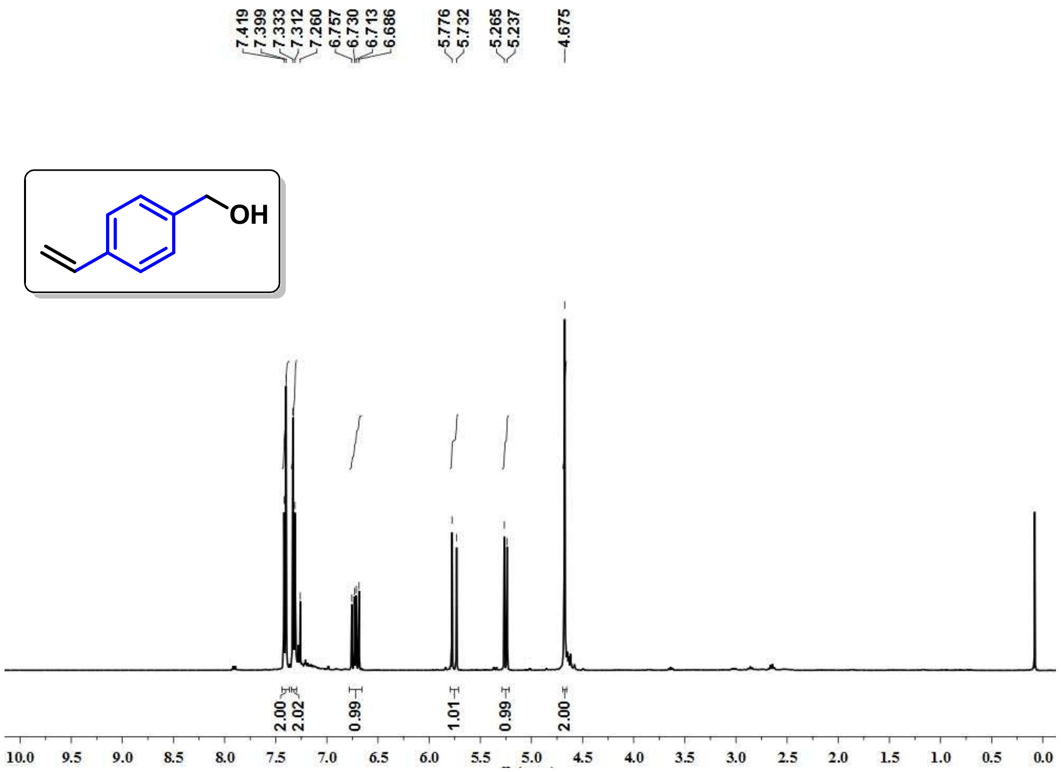

Figure S28. ^{1}H NMR spectrum of 4-vinylbenzyl alcohol, **4k** recorded in $CDCl_3$.

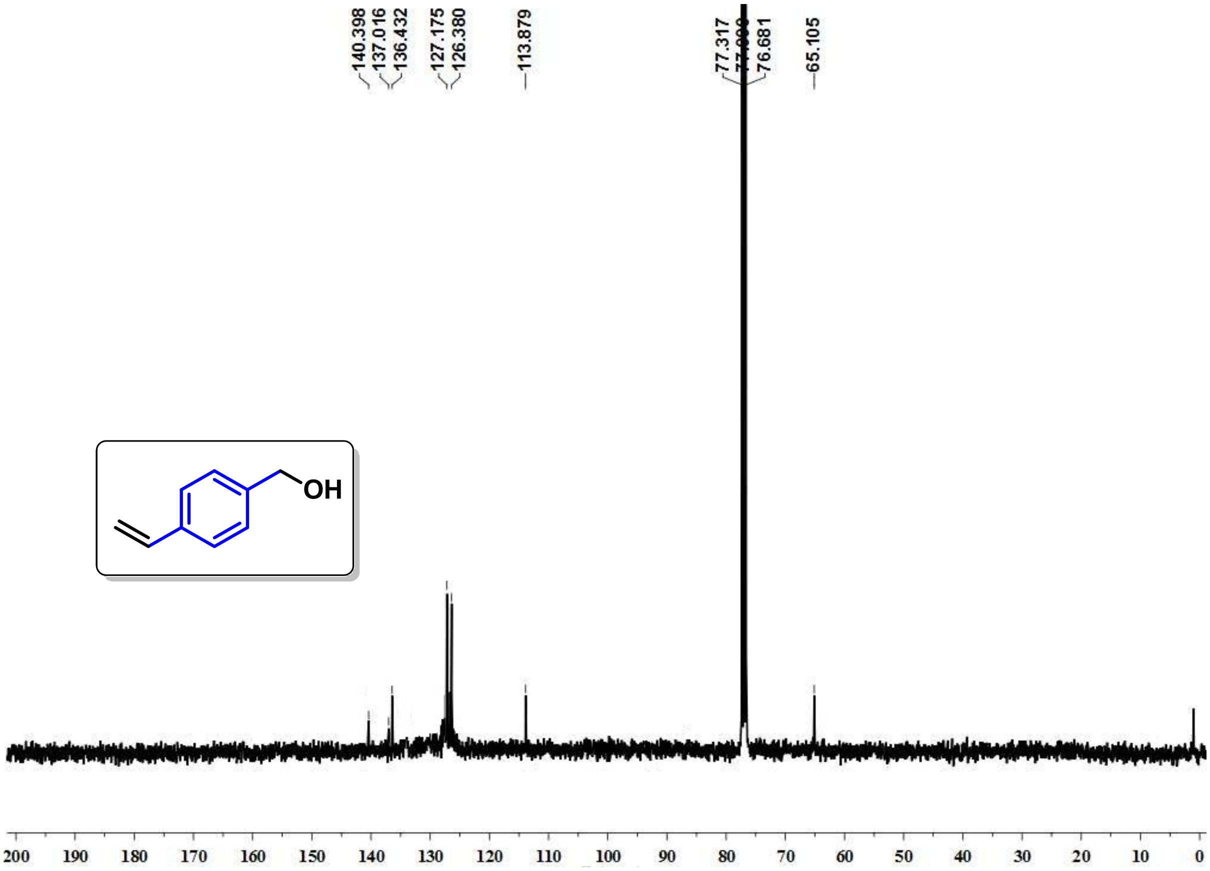

Figure S29. ^{13}C NMR of 4-vinylbenzyl alcohol, **4k** recorded in $CDCl_3$.

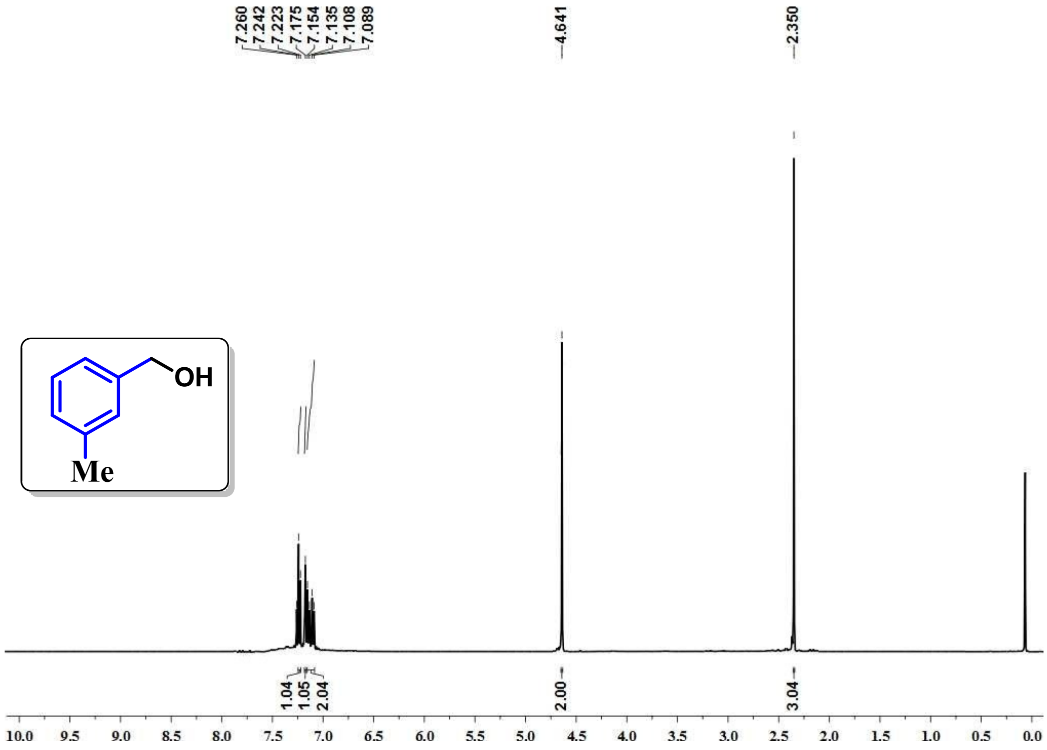

Figure S30. ^{1}H NMR spectrum of 3-methylbenzyl alcohol, **4l** recorded in $CDCl_3$.

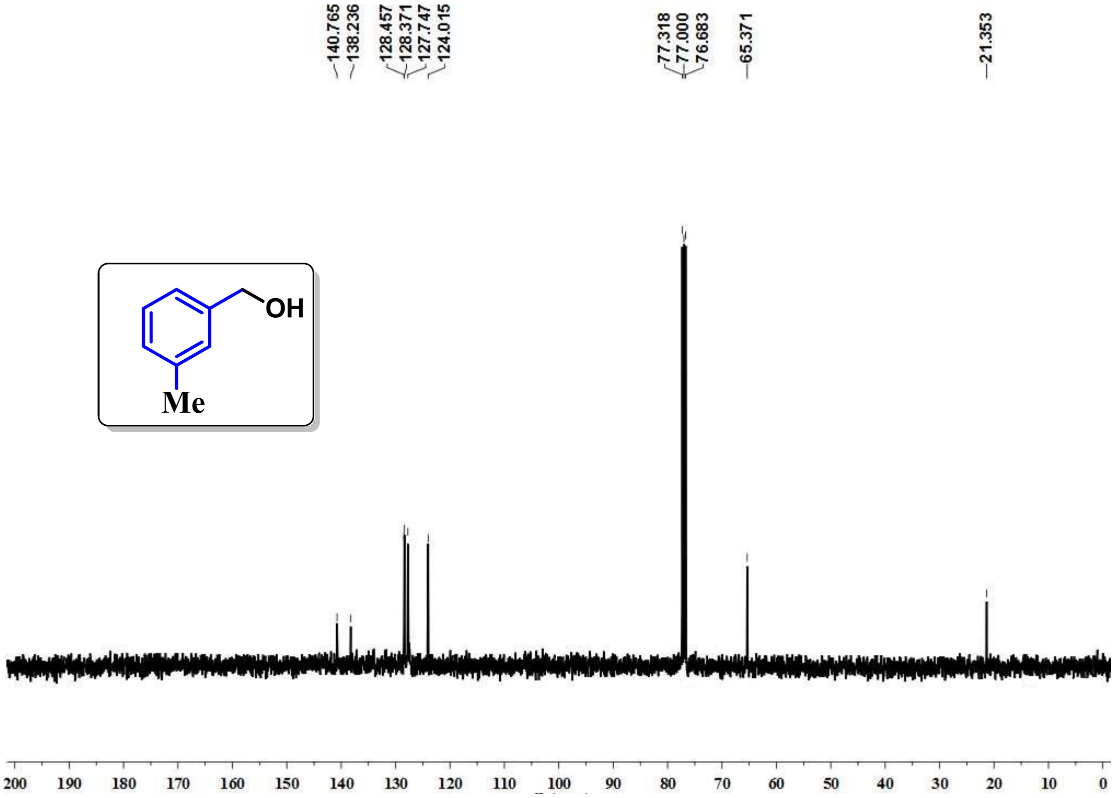

Figure S31. ^{13}C NMR spectrum of 3-methylbenzyl alcohol, **4l** recorded in $CDCl_3$.

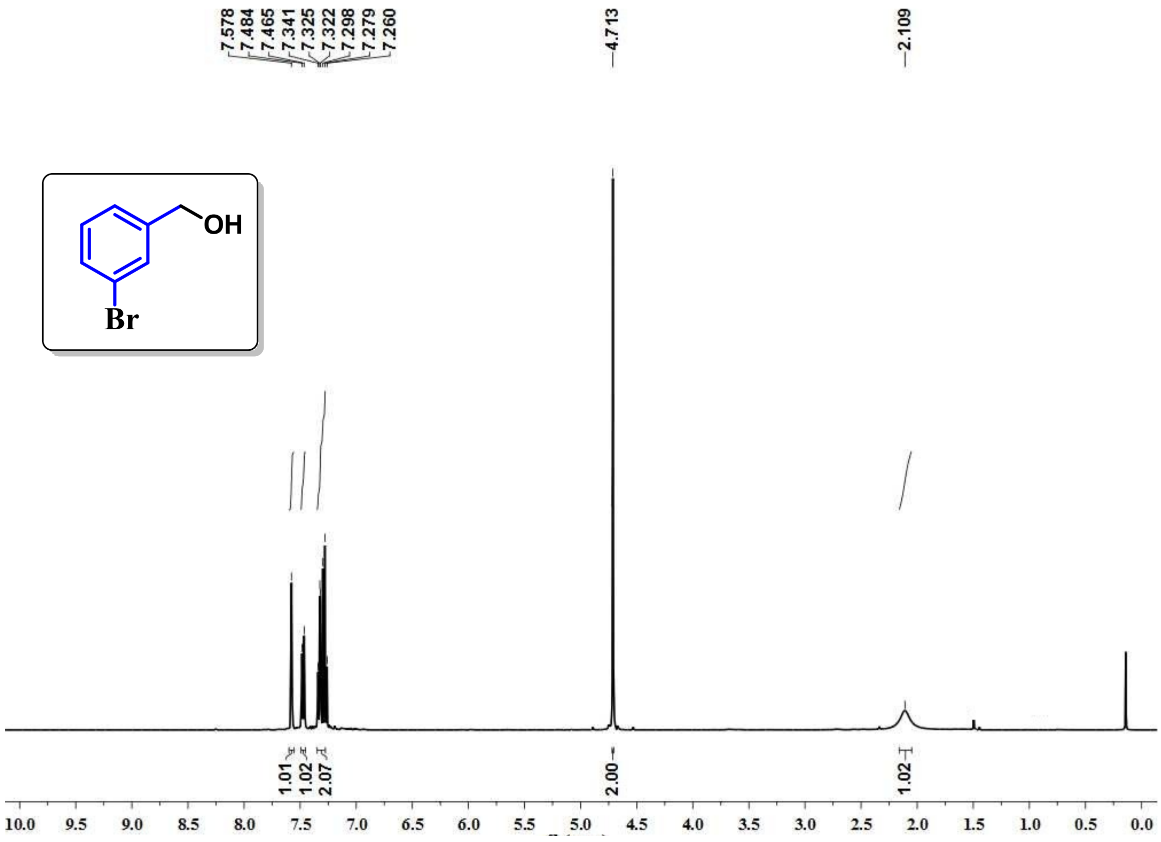

Figure S32. ^{1}H NMR spectrum of 3-bromobenzyl alcohol, **4n** recorded in $CDCl_3$.

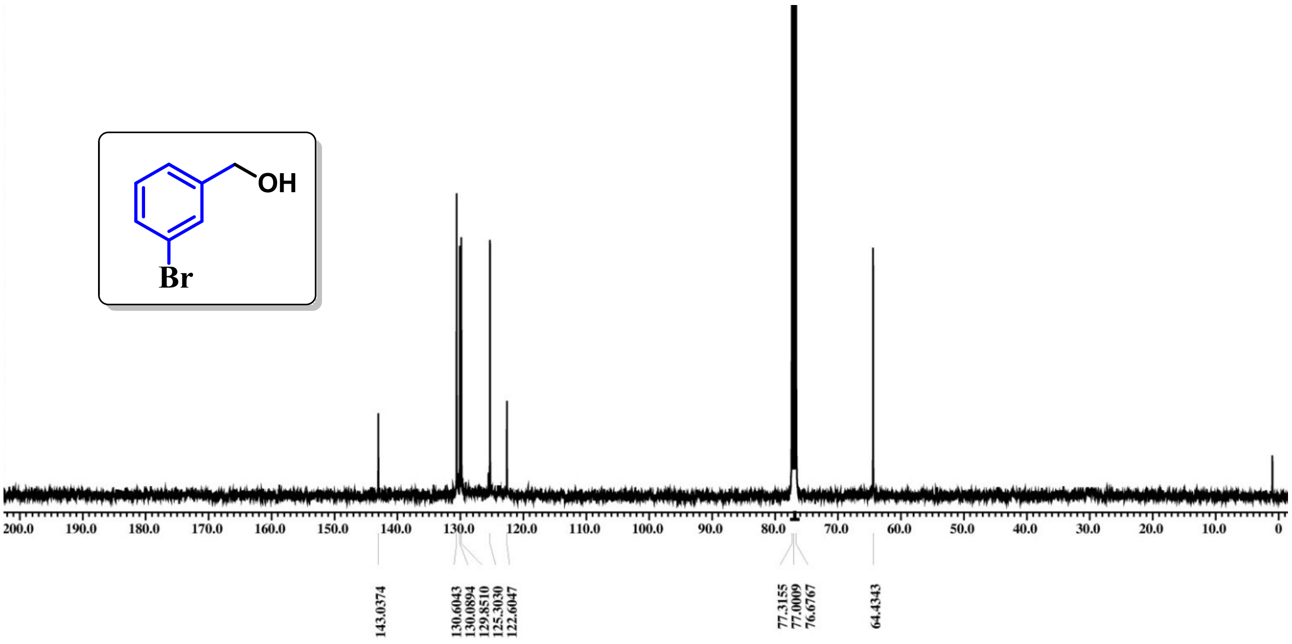

Figure S33. ^{13}C NMR spectrum of 3-bromobenzyl alcohol, **4n** recorded in $CDCl_3$.

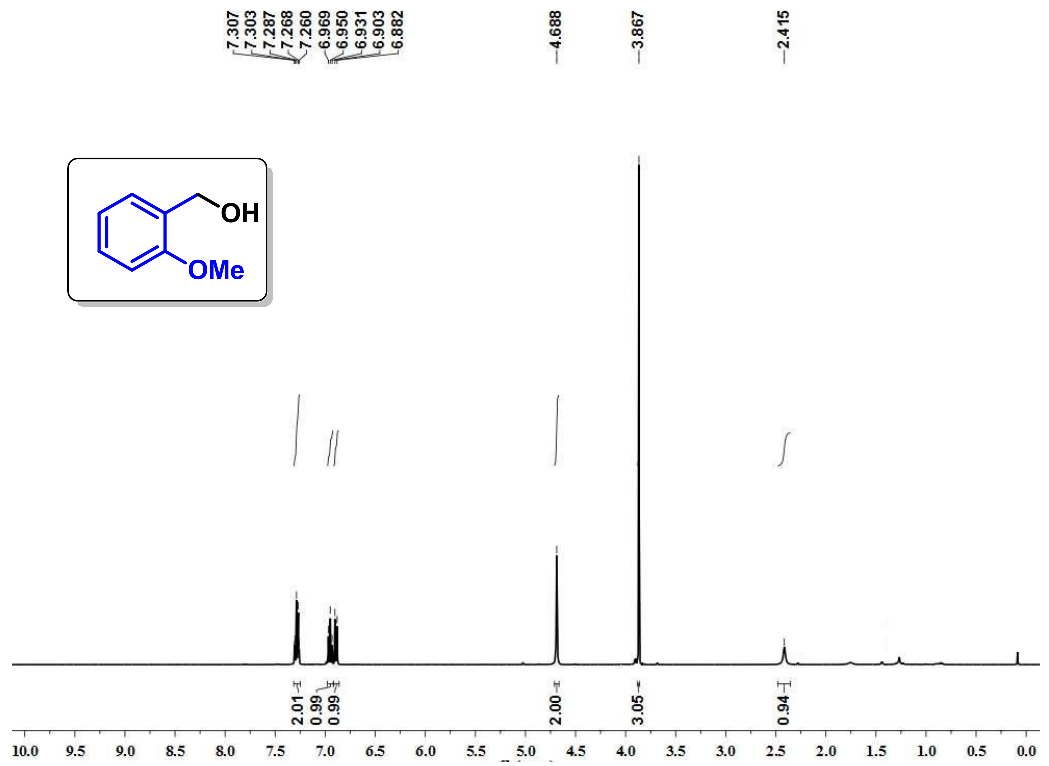

Figure S34. ^{1}H NMR spectrum of 2-methoxybenzyl alcohol, **4o** recorded in $CDCl_3$.

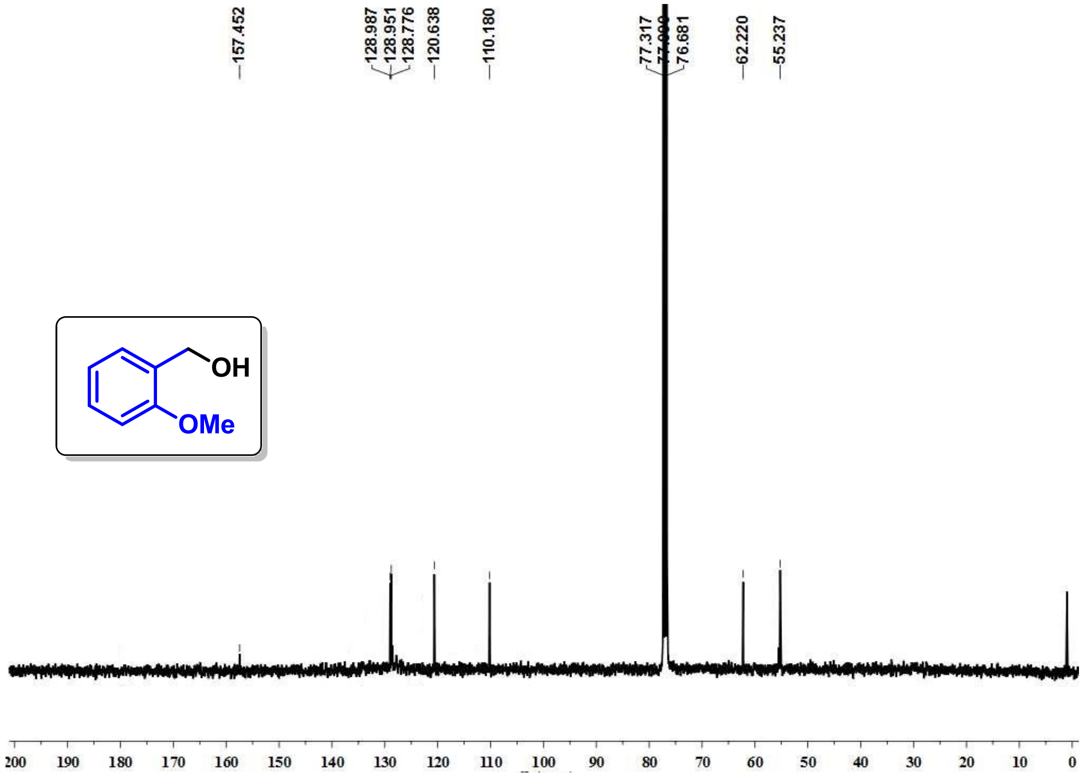

Figure S35. ^{13}C NMR spectrum of 2-methoxybenzyl alcohol, **4o** recorded in $CDCl_3$.

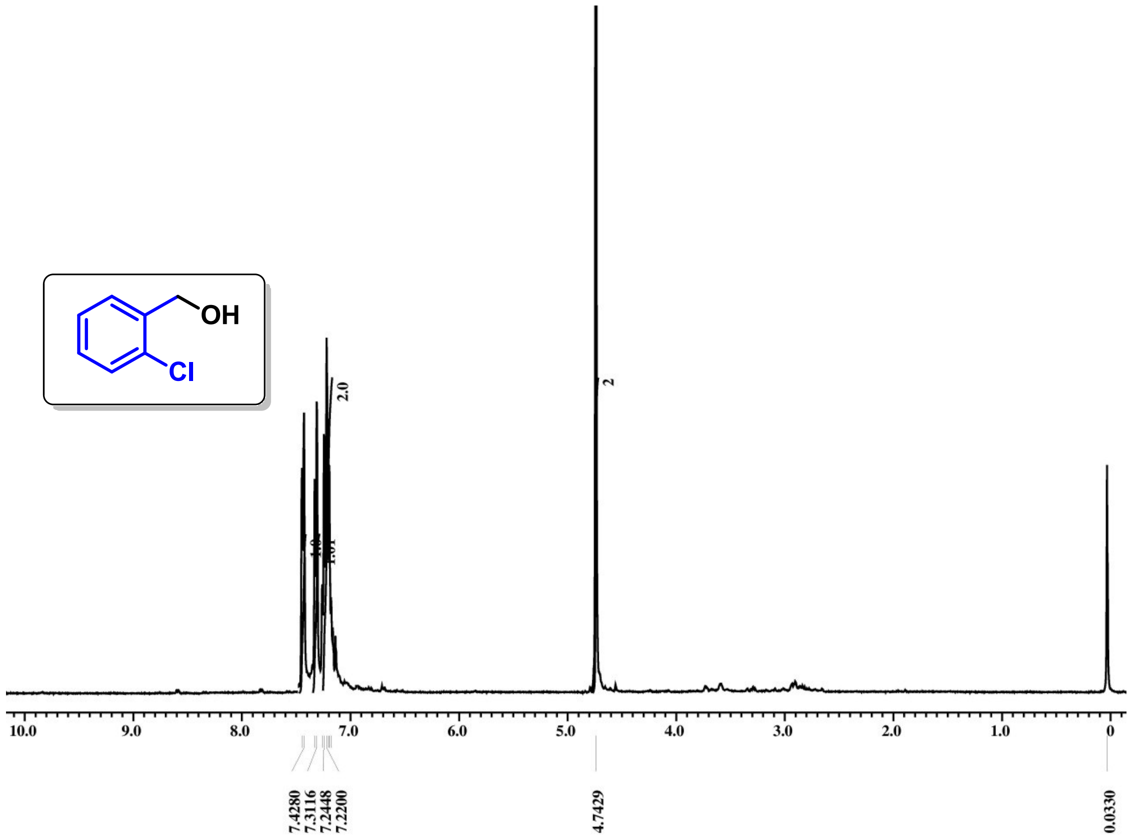

Figure S36. ^{1}H NMR spectrum of 2-chlorobenzyl alcohol, **4p** recorded in $CDCl_3$.

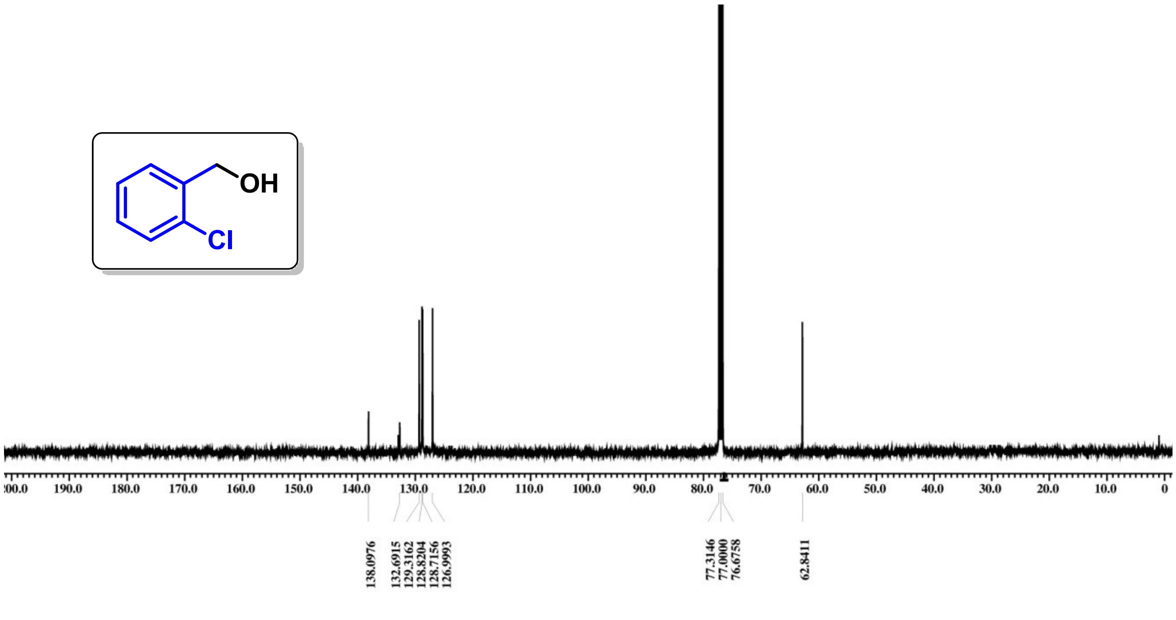

Figure S37. ^{13}C NMR spectrum of 2-chlorobenzyl alcohol, **4p** recorded in $CDCl_3$.

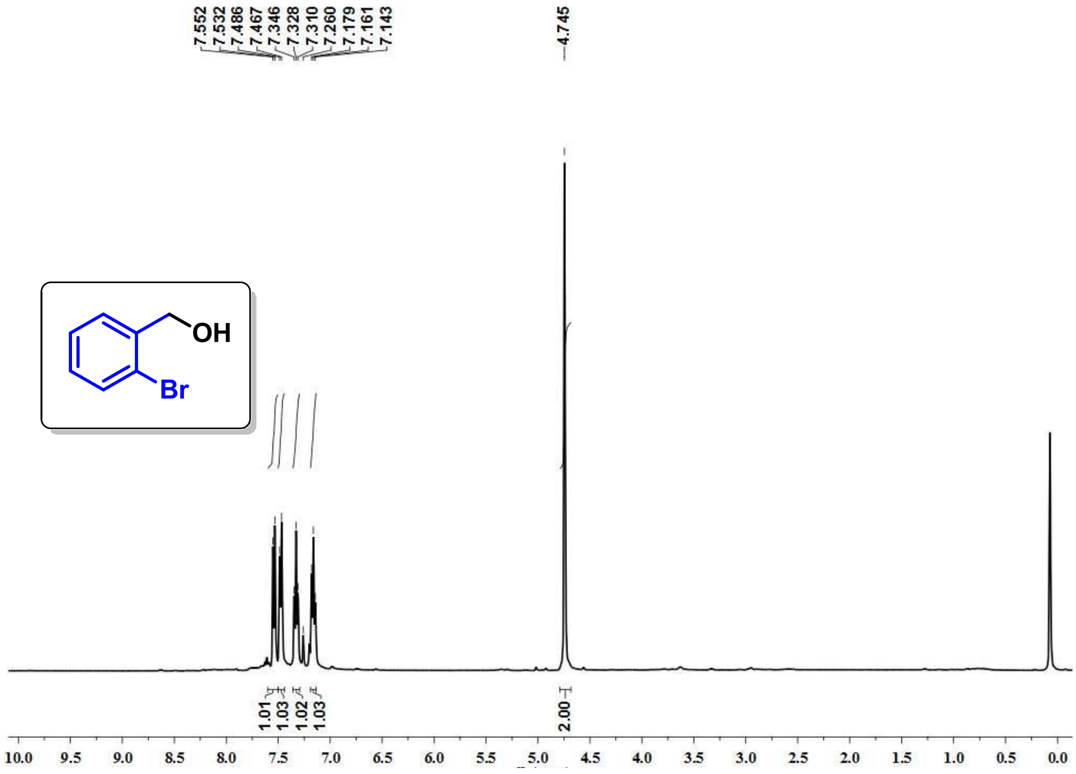

Figure S38. ^{1}H NMR spectrum of 2-bromobenzyl alcohol, **4q** recorded in $CDCl_3$.

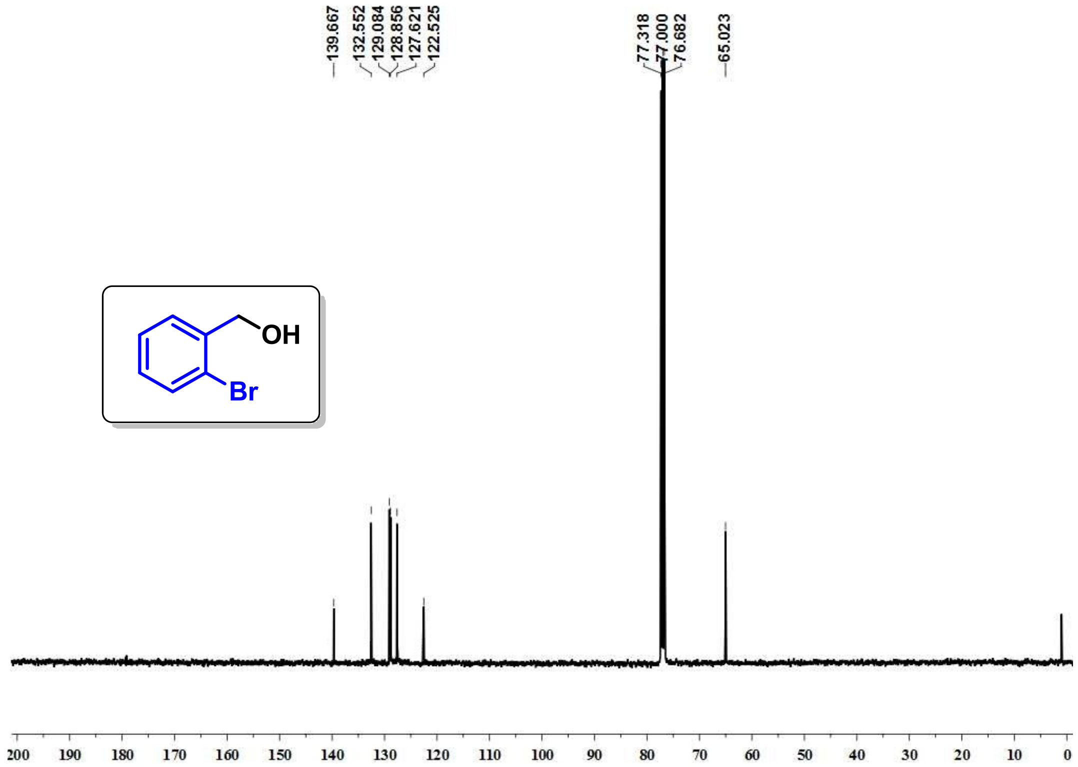

Figure S39. ^{1}H NMR spectrum of 2-bromobenzyl alcohol, **4q** recorded in $CDCl_3$.

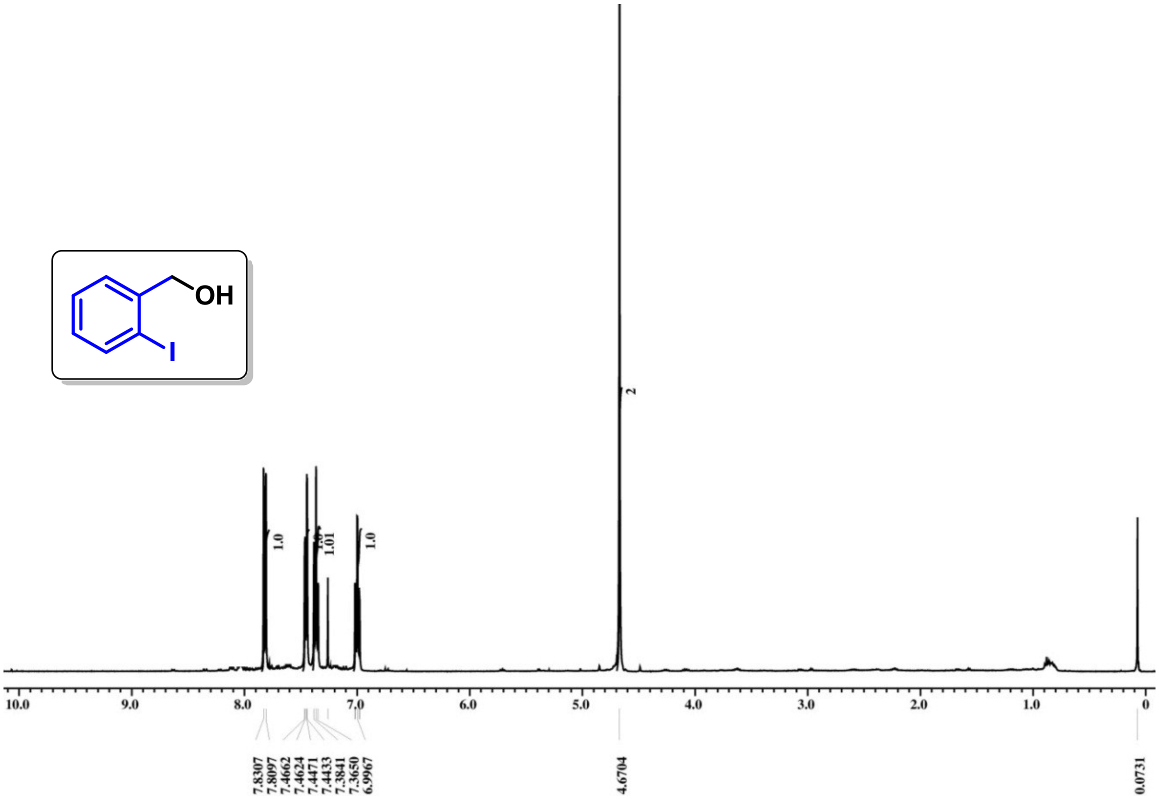

Figure S40. ^{1}H NMR spectrum of 2-iodobenzyl alcohol, **4r** recorded in $CDCl_3$.

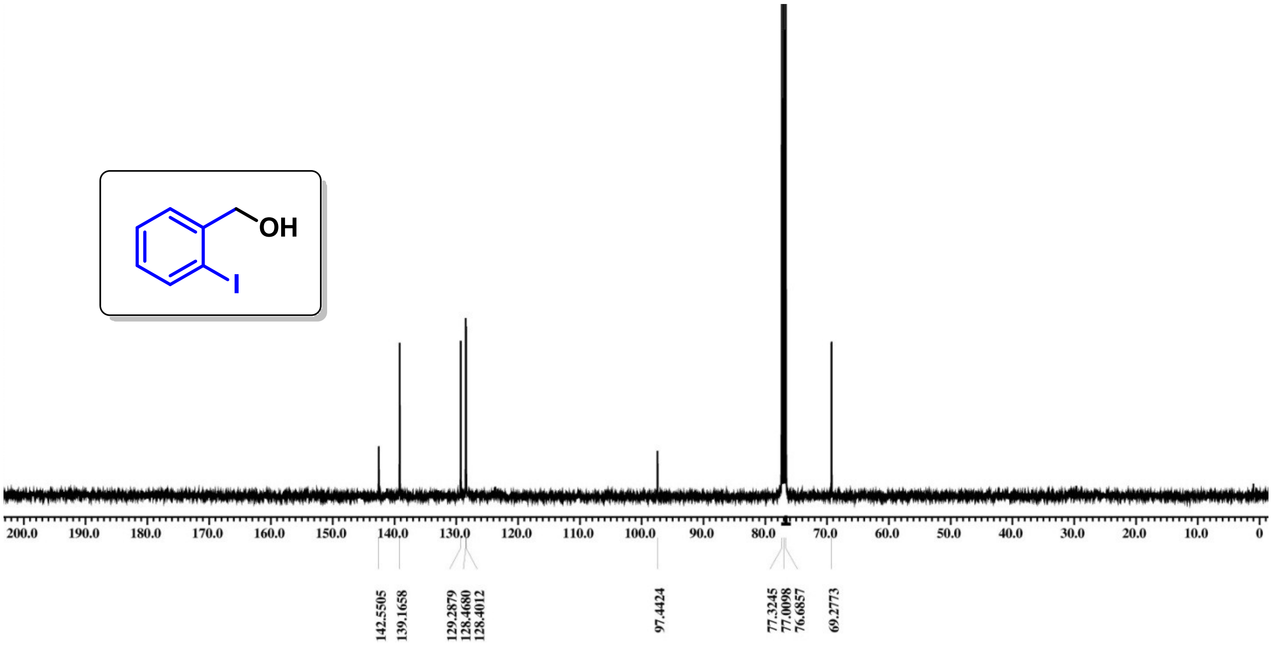

Figure S41. ^{13}C NMR spectrum of 2-iodobenzyl alcohol, **4r** recorded in $CDCl_3$.

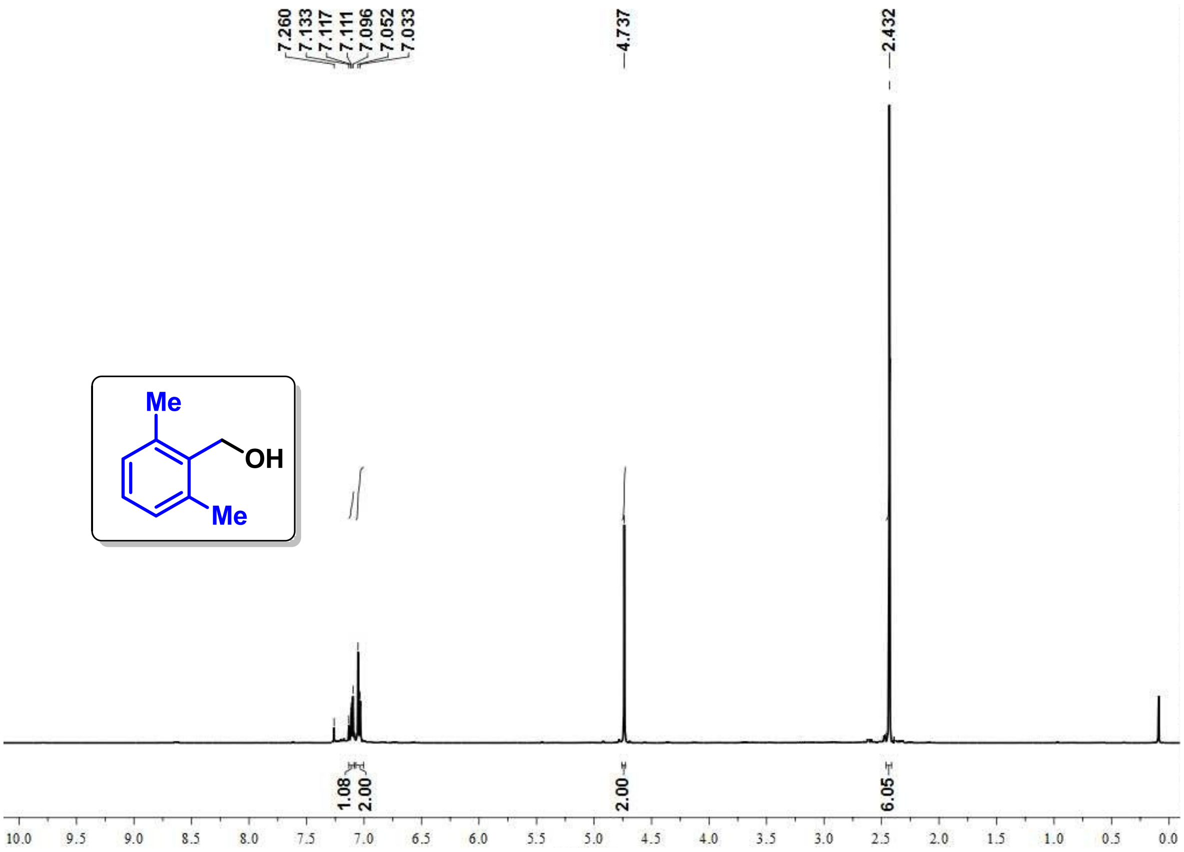

Figure S42. ^{1}H NMR spectrum of 2,6-dimethylbenzyl alcohol, **4s** recorded in $CDCl_3$.

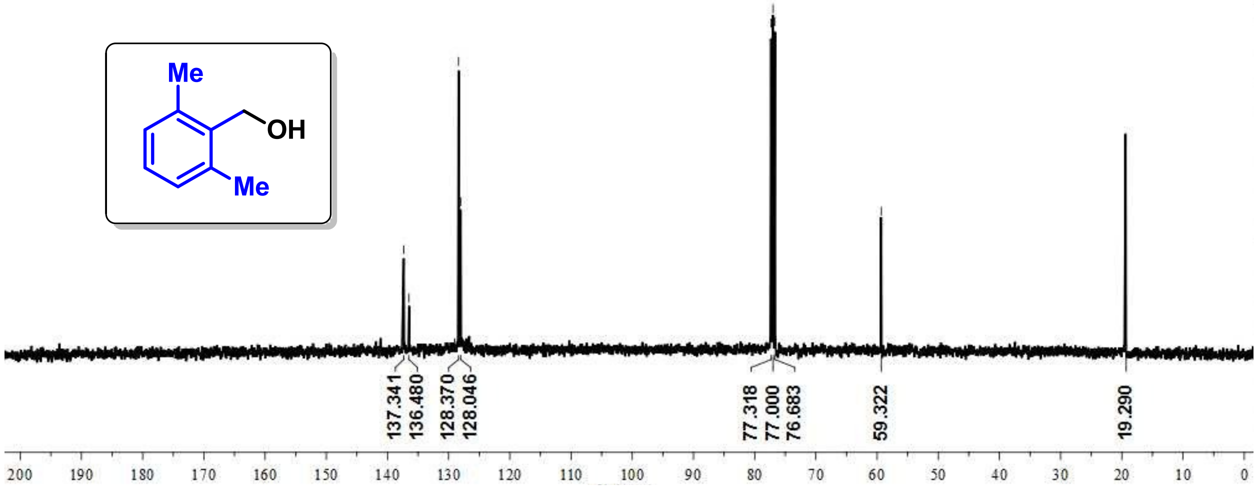

Figure S43. ^{13}C NMR spectrum of 2,6-dimethylbenzyl alcohol, **4s** recorded in $CDCl_3$.

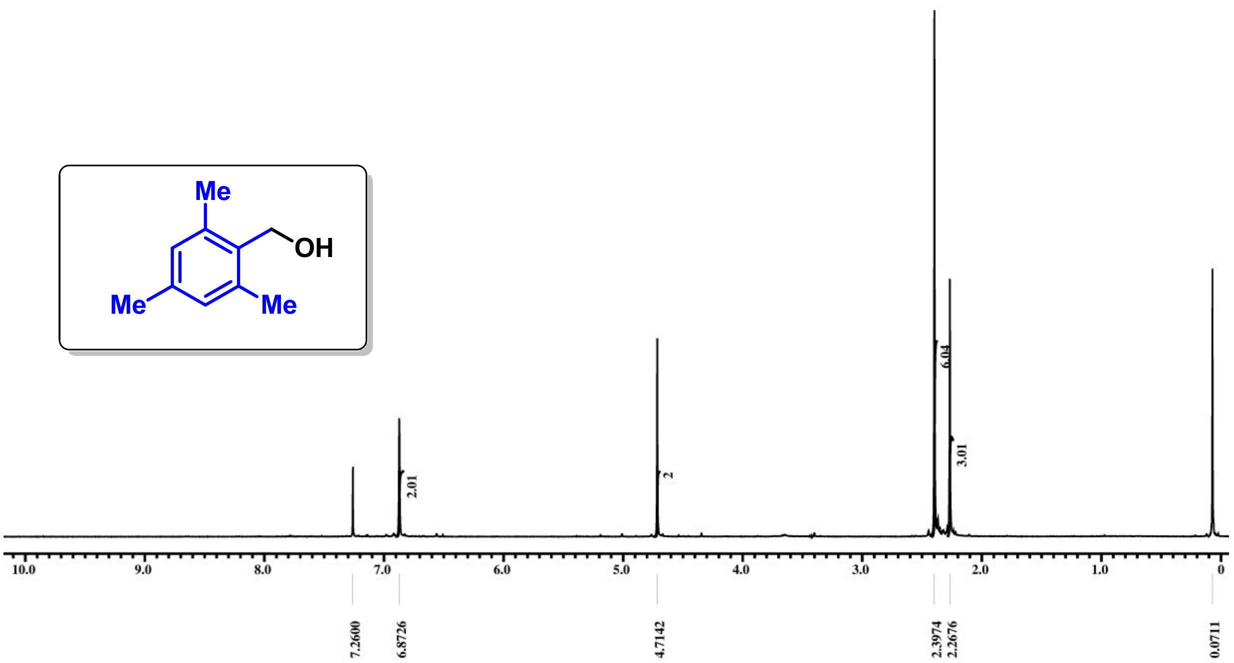

Figure S44. ¹H NMR spectrum of 2,4,6-trimethylbenzyl alcohol, **4u** recorded in $CDCl_3$.

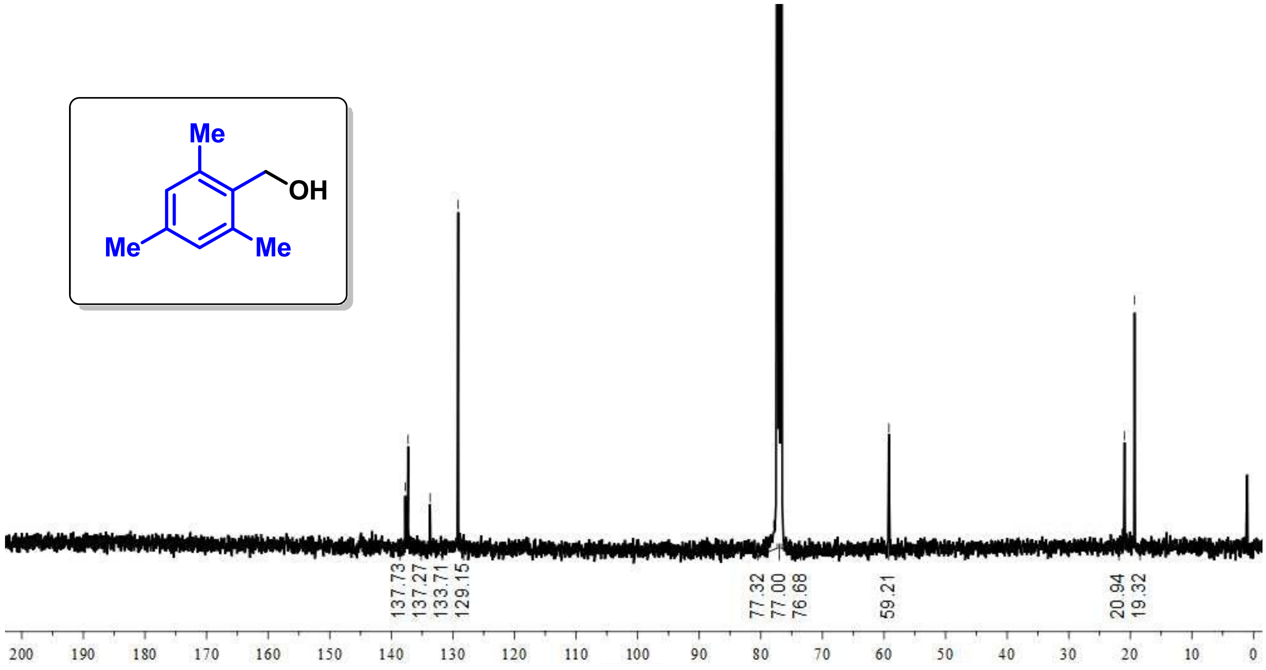

Figure S45. ¹³C NMR spectrum of 2,4,6-trimethylbenzyl alcohol, **4u** recorded in $CDCl_3$.

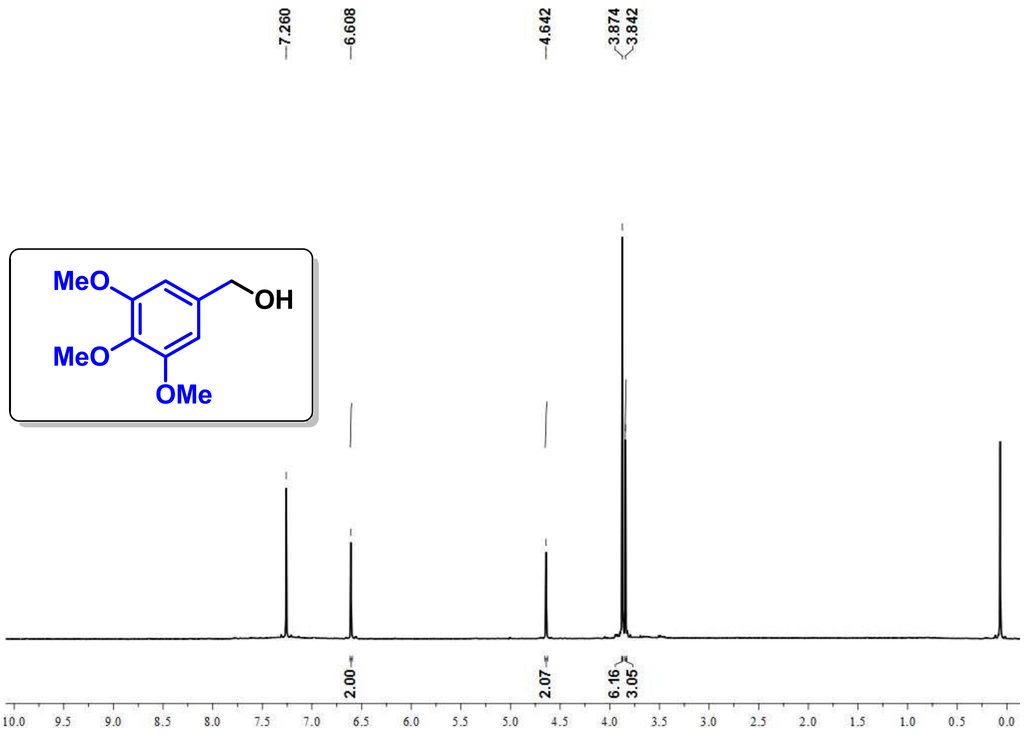

Figure S46. ^{1}H NMR spectrum of 3,4,5-trimethoxybenzyl alcohol, **4v** recorded in $CDCl_3$.

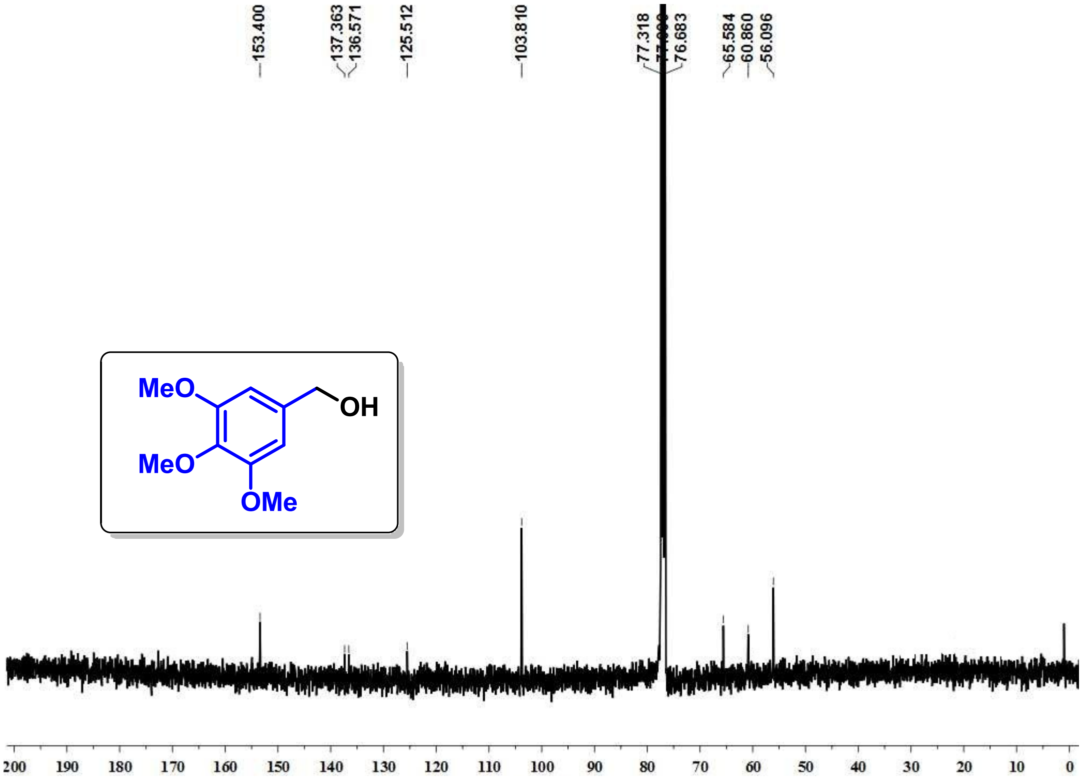

Figure S47. ^{13}C NMR spectrum of 3,4,5-trimethoxybenzyl alcohol, **4v** recorded in $CDCl_3$.

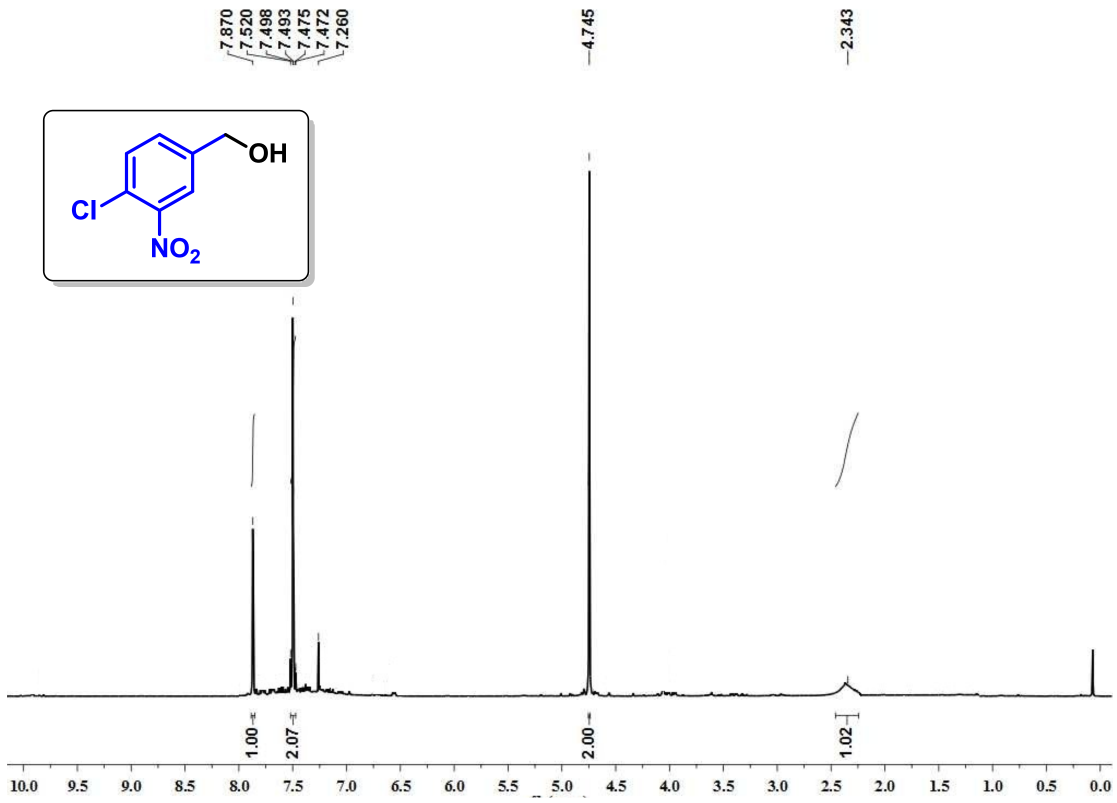

Figure S48. ^{1}H NMR spectrum of 4-chloro-3-nitrobenzyl alcohol, **4w** recorded in $CDCl_3$.

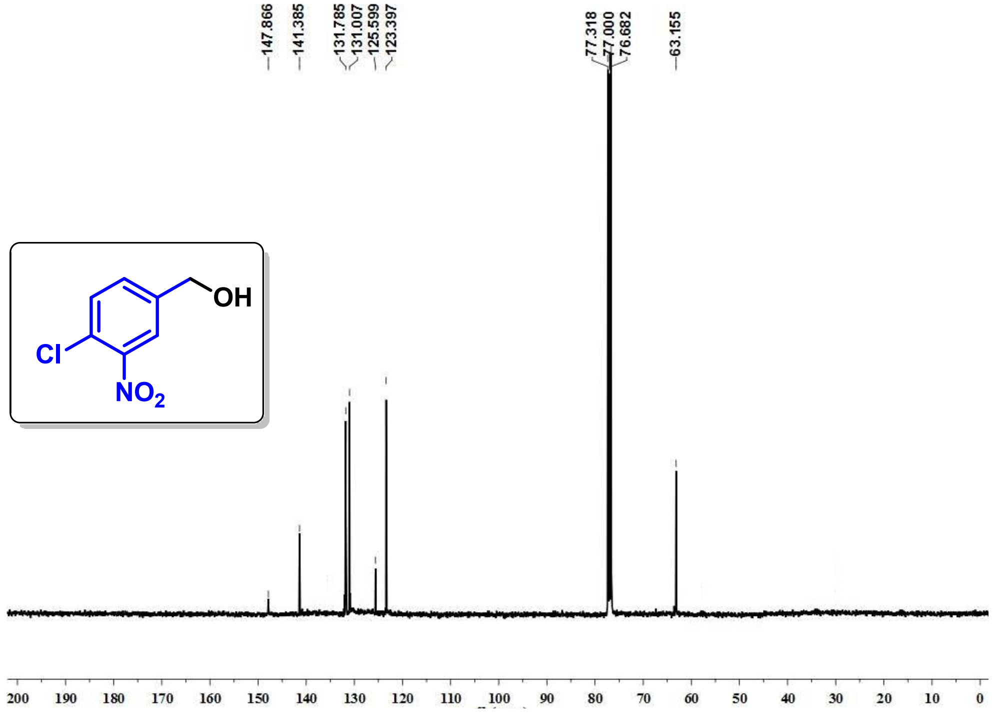

Figure S49. ^{13}C NMR spectrum of 4-chloro-3-nitrobenzyl alcohol, **4w** recorded in $CDCl_3$.

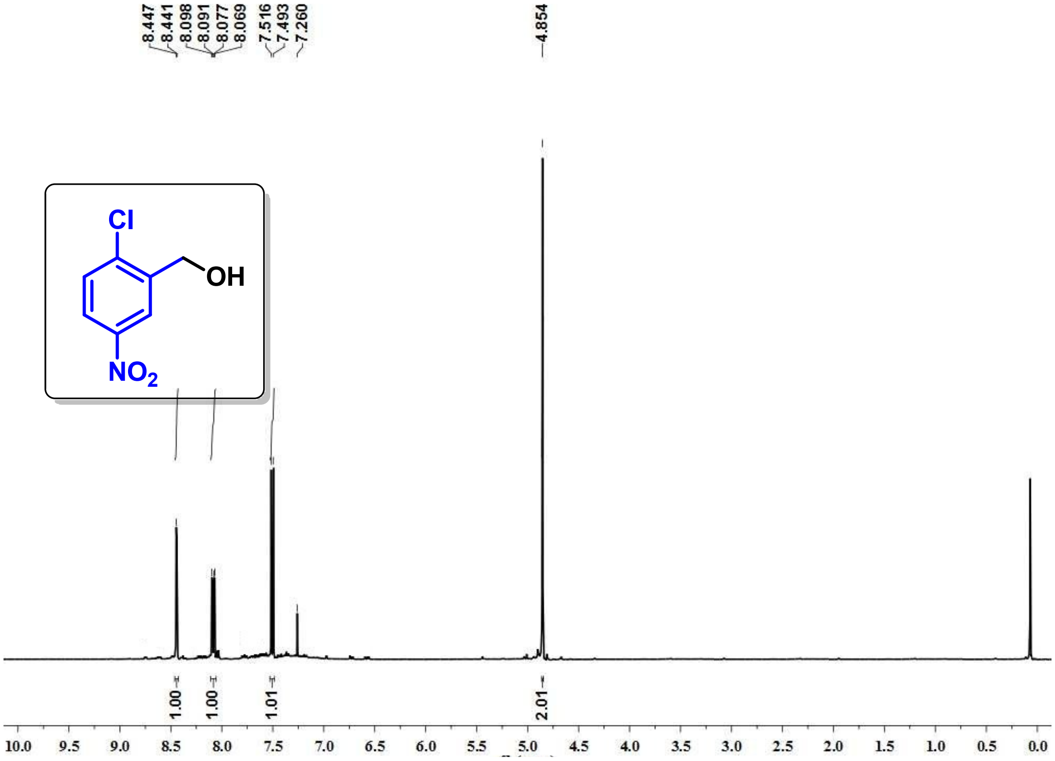

Figure S50. ^{1}H NMR spectrum of 2-chloro-5-nitrobenzyl alcohol, **4x** recorded in $CDCl_3$.

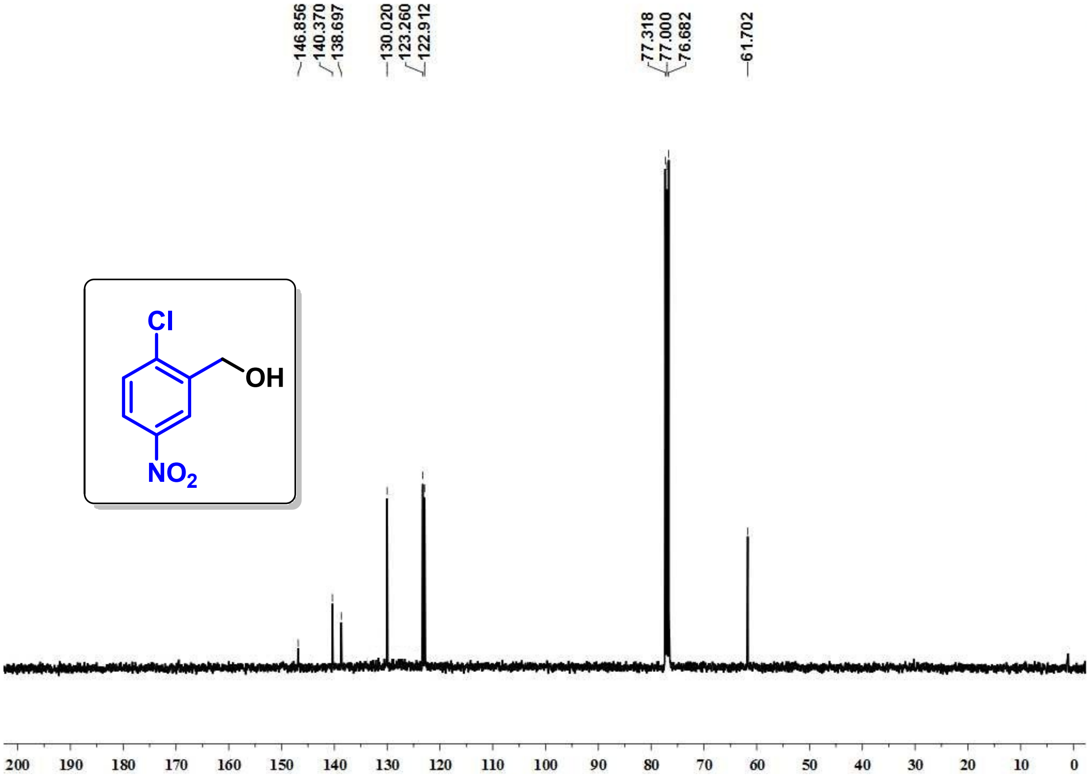

Figure S51. ^{13}C NMR spectrum of 2-chloro-5-nitrobenzyl alcohol, **4x** recorded in $CDCl_3$.

12. Spectroscopic characterization of stoichiometric reaction.

12a. Radical trapping experiment through TEMPO adducts formation.

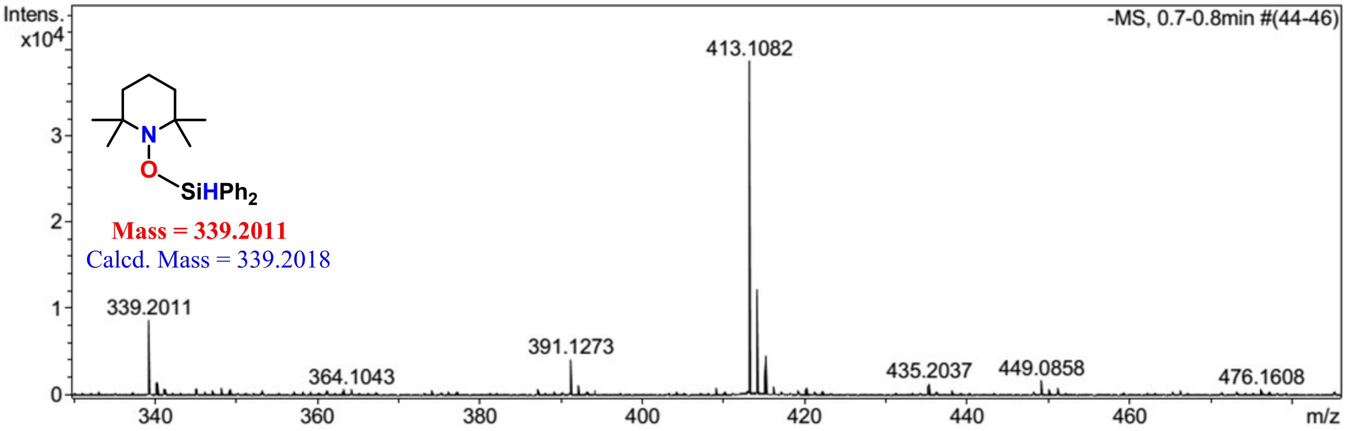

Figure S52. TEMPO trapped Ph_2SiH radical.

12b. NMR spectra of PLY-dimer (5).

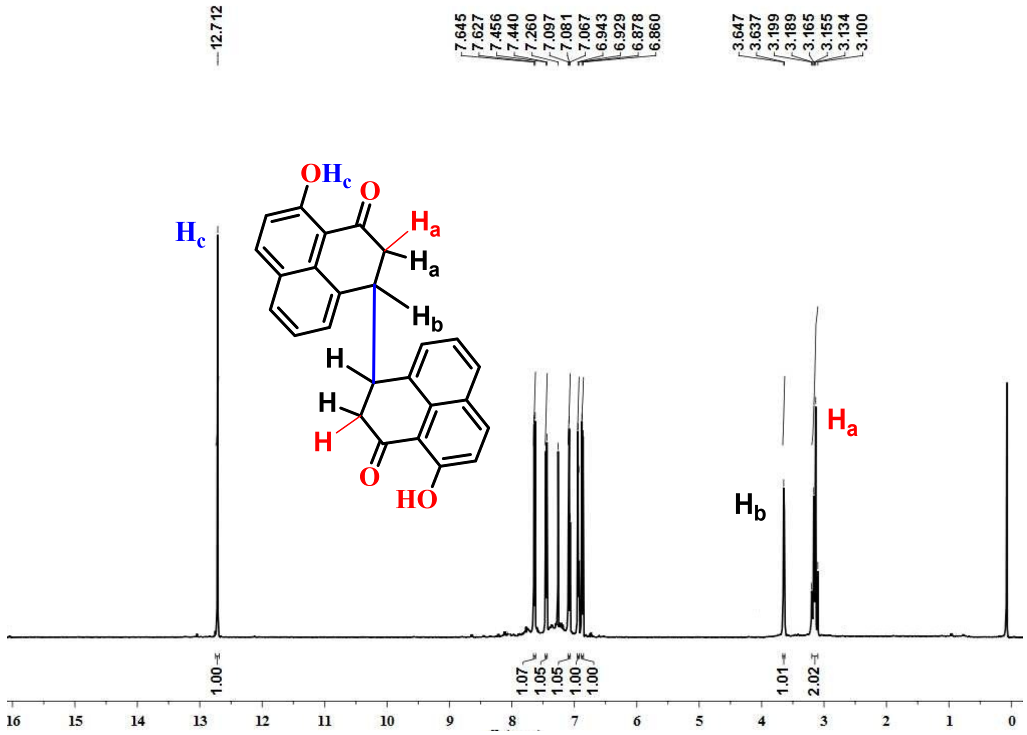

Figure S53. ^{1}H NMR spectrum of PLY-dimer, **5** recorded in $CDCl_3$.

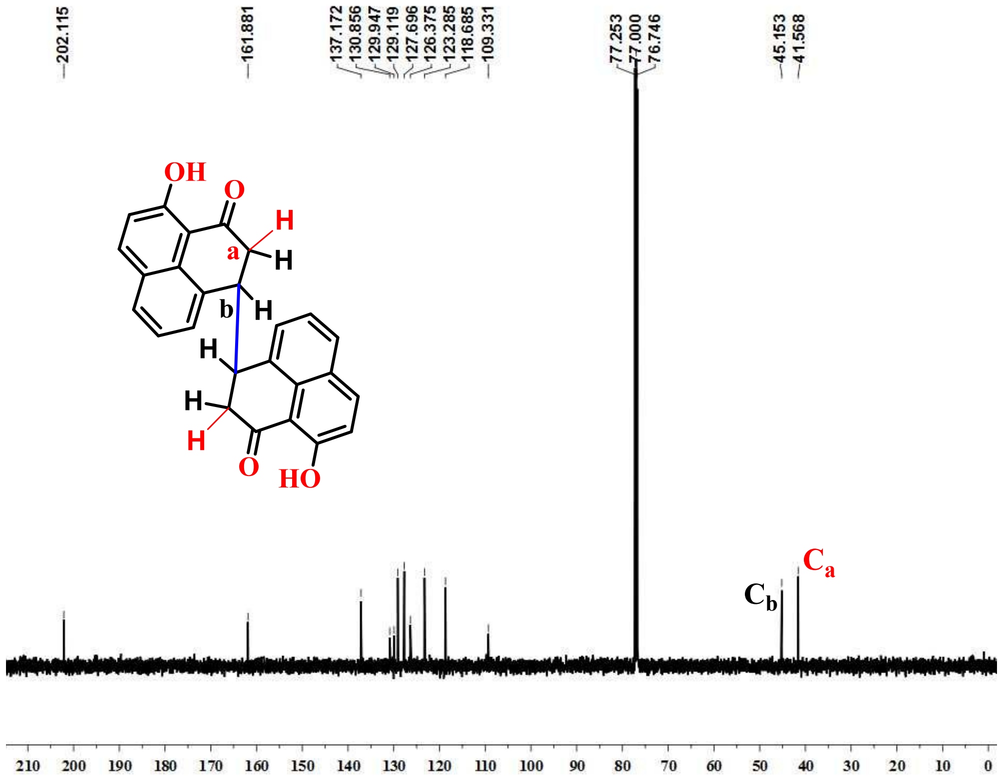

Figure S54. ^{13}C NMR spectrum of PLY-dimer, **5** recorded in $CDCl_3$.

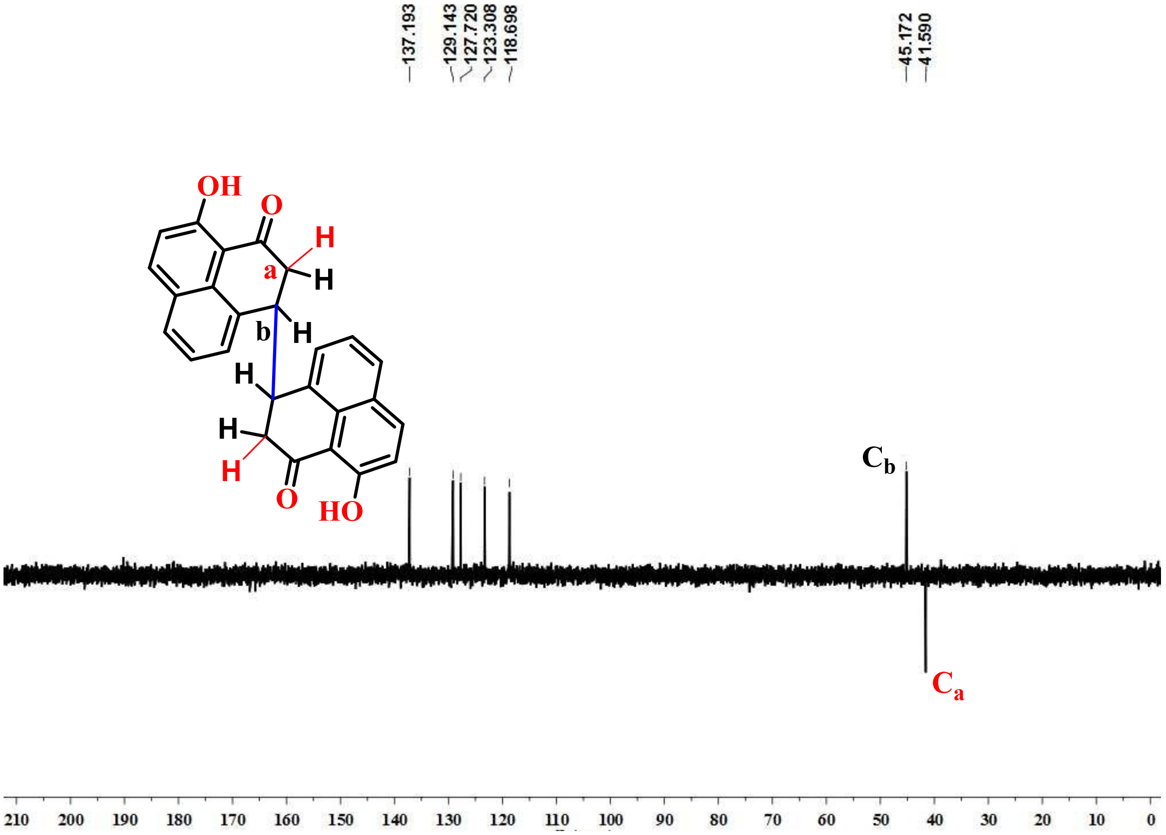

Figure S55. DEPT-135 NMR spectrum of PLY-dimer, **5** recorded in $CDCl_3$.

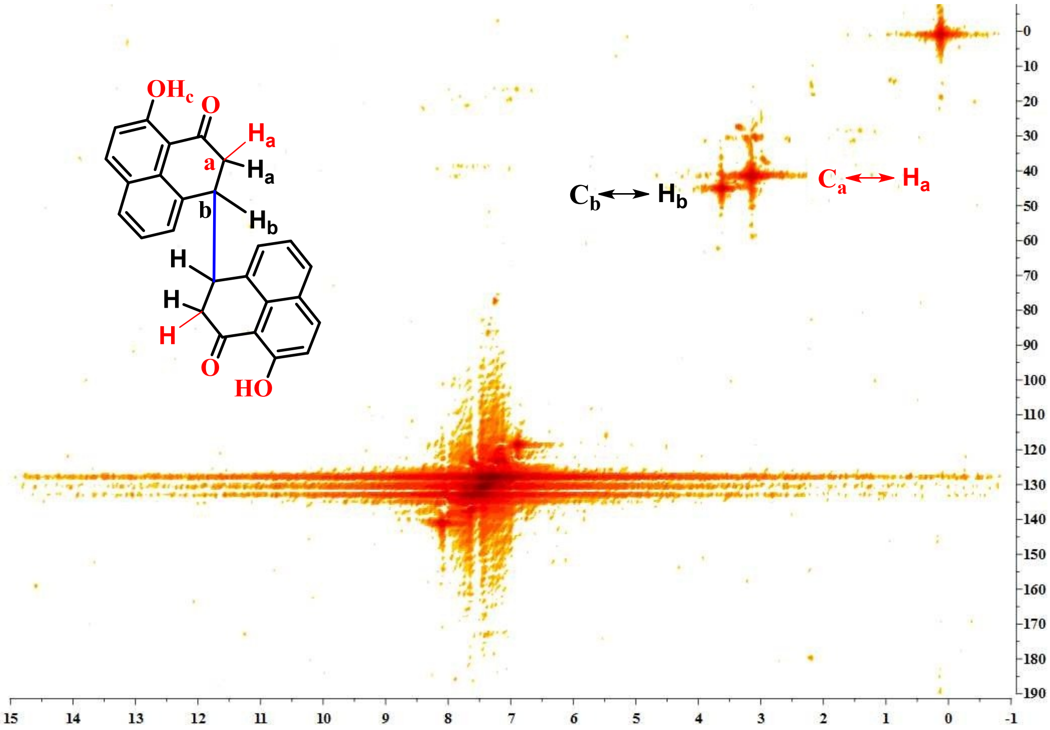

Figure S56. HMQC NMR of PLY-dimer, **5** in $CDCl_3$.

NMR spectrum of 9-hydroxyphenalenone.[S26]

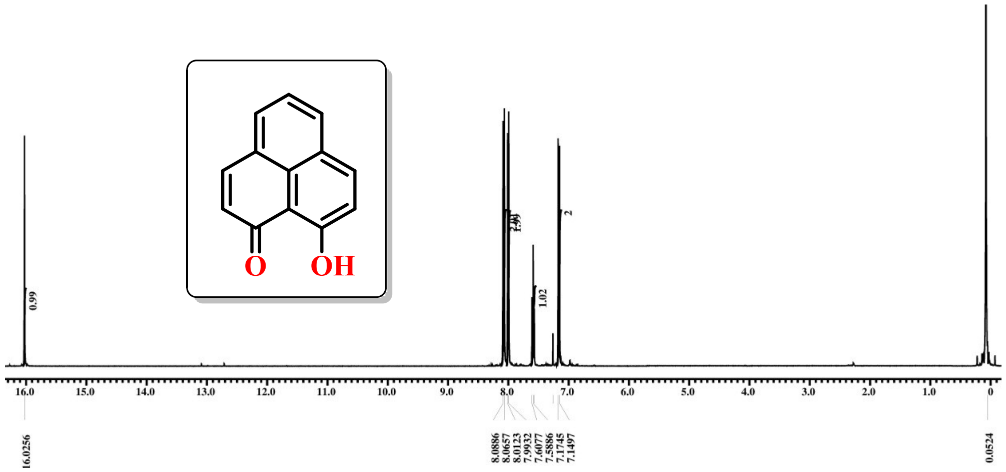

Figure S57. ^{1}H NMR spectrum of 9-hydroxyphenalenone recorded in $CDCl_3$.

12c. Detection of H_2 gas through ^{1}H NMR spectroscopy.

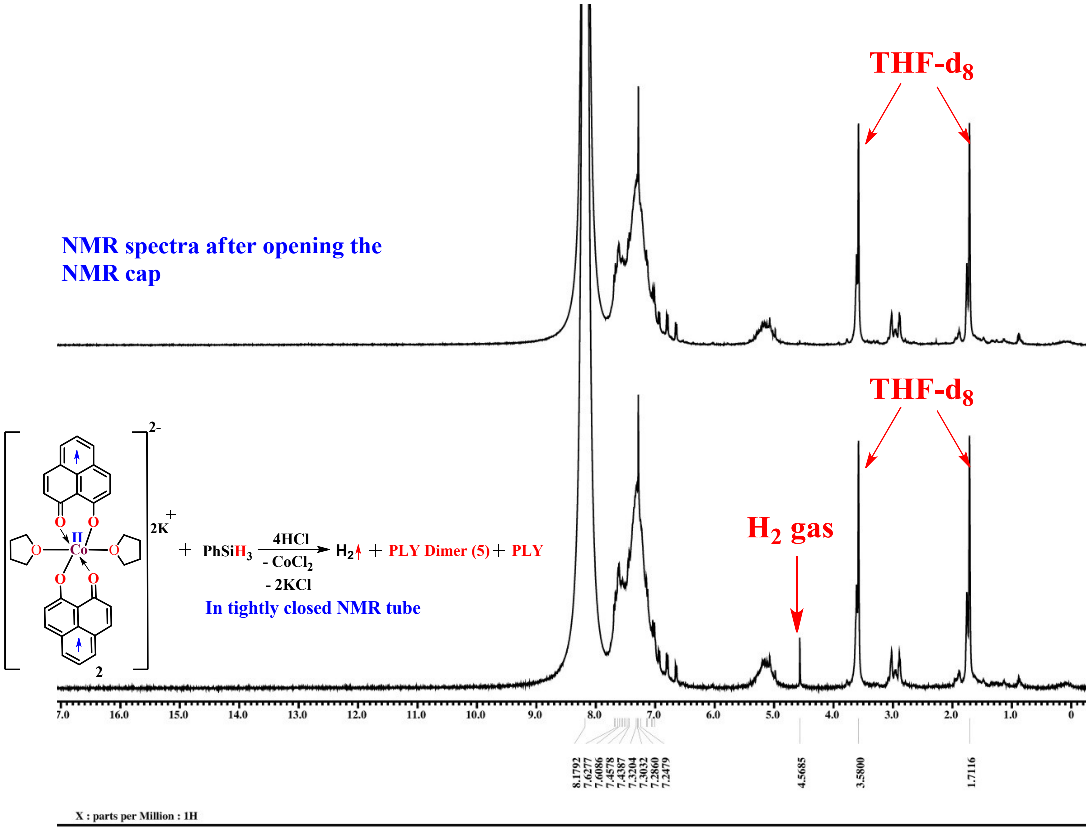

Figure S58. Stack plot of recorded ^{1}H NMR spectrum in THF-d_8 for detection of dihydrogen gas formation in a tightly closed NMR tube and immediately recorded ^{1}H NMR spectrum after removal of NMR tube cap in THF-d_8.

12d. NMR spectra of PLY Dimer-D2 (5_D).

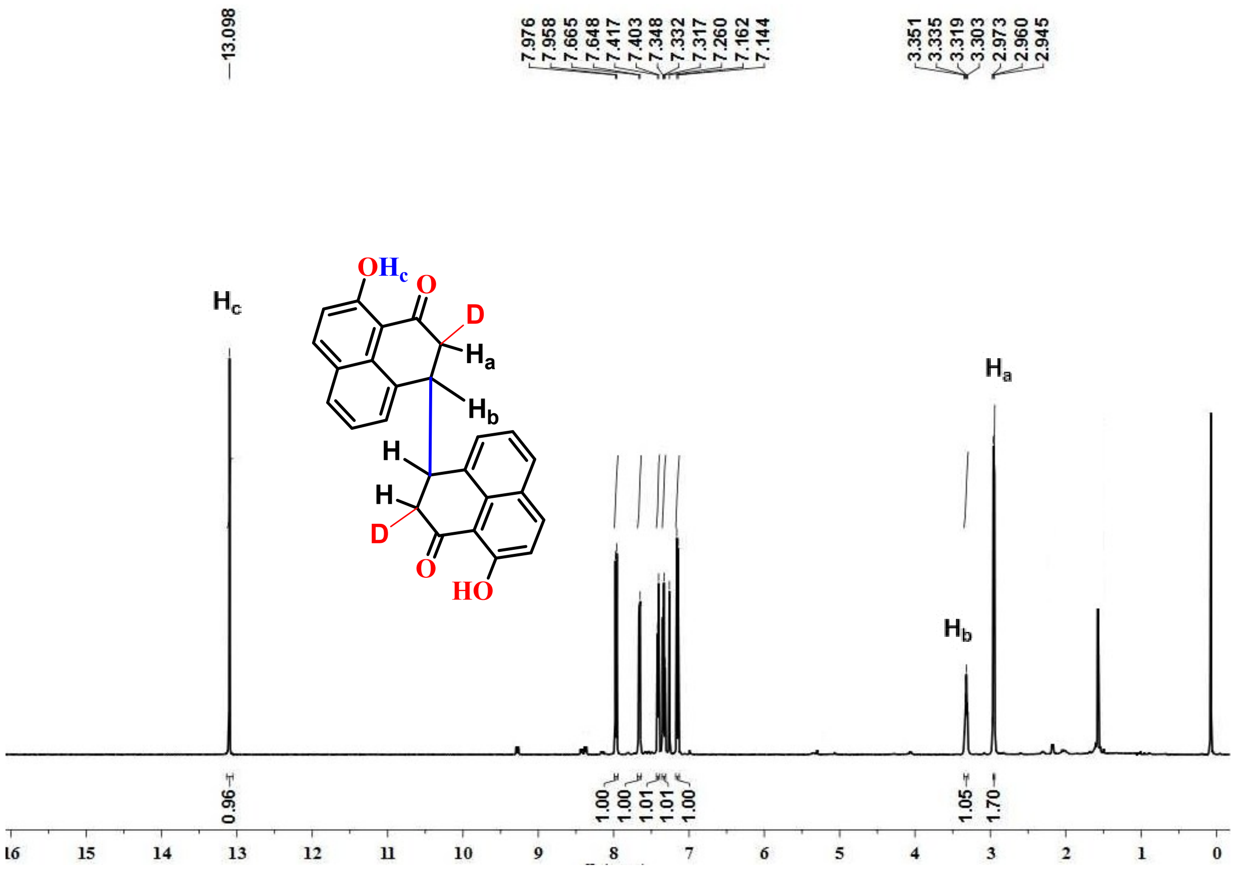

Figure S59. ^{1}H NMR spectrum of PLY dimer-D_2, $\mathbf{5_D}$ recorded in $CDCl_3$.

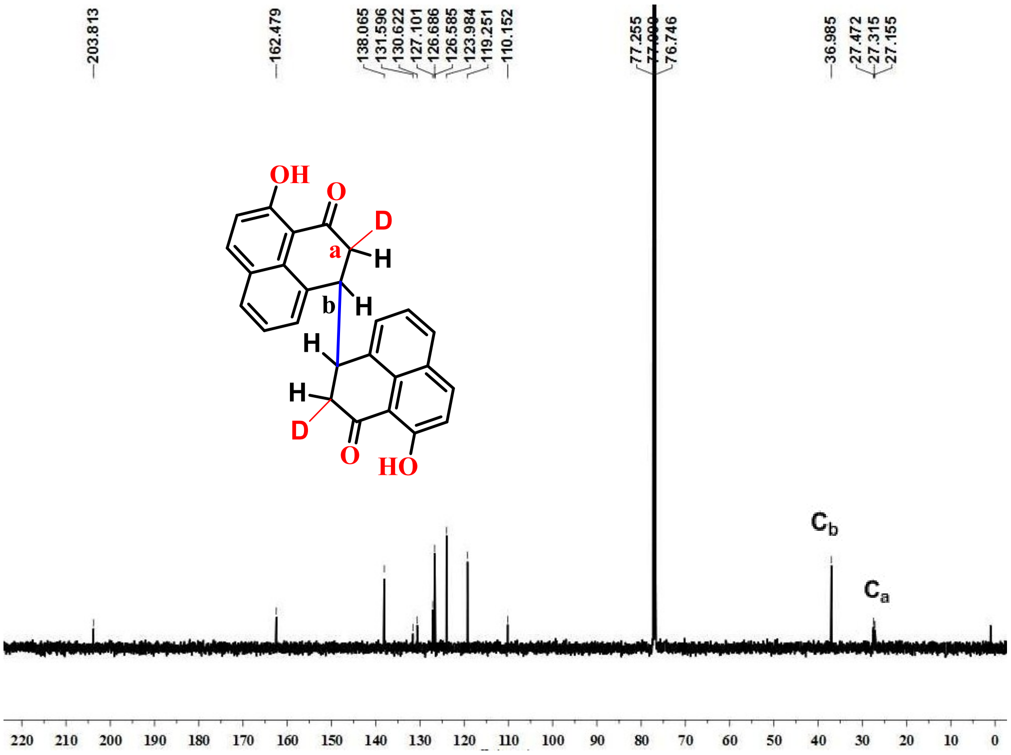

Figure S60. ^{13}C NMR spectrum of PLY dimer-D$_2$, **5$_D$** recorded in $CDCl_3$.

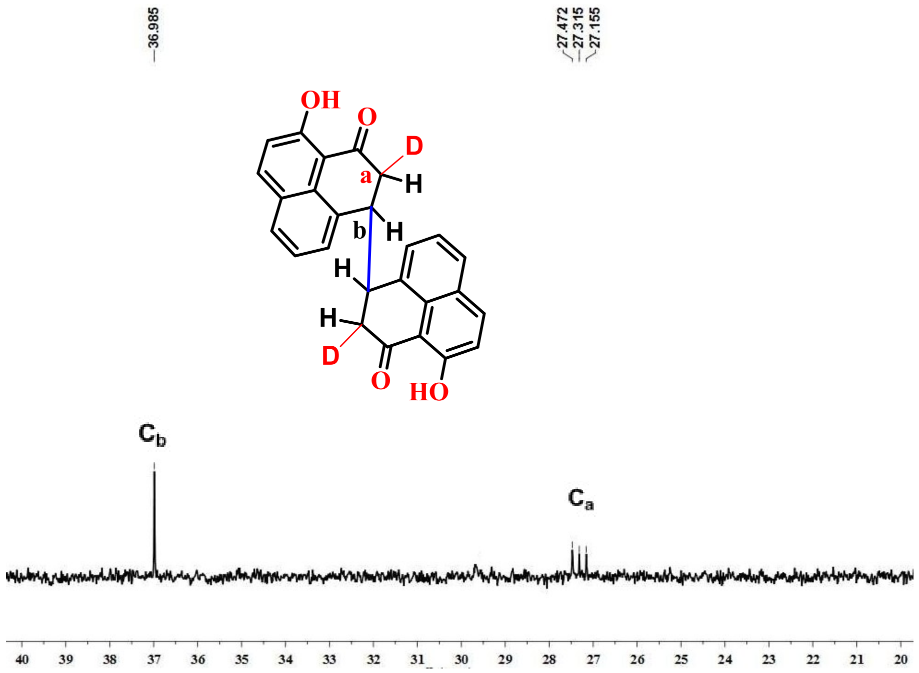

Figure S61. Zoomed version of ^{13}C NMR spectrum (40–20 ppm) of PLY dimer-D$_2$, **5$_D$** recorded in $CDCl_3$.

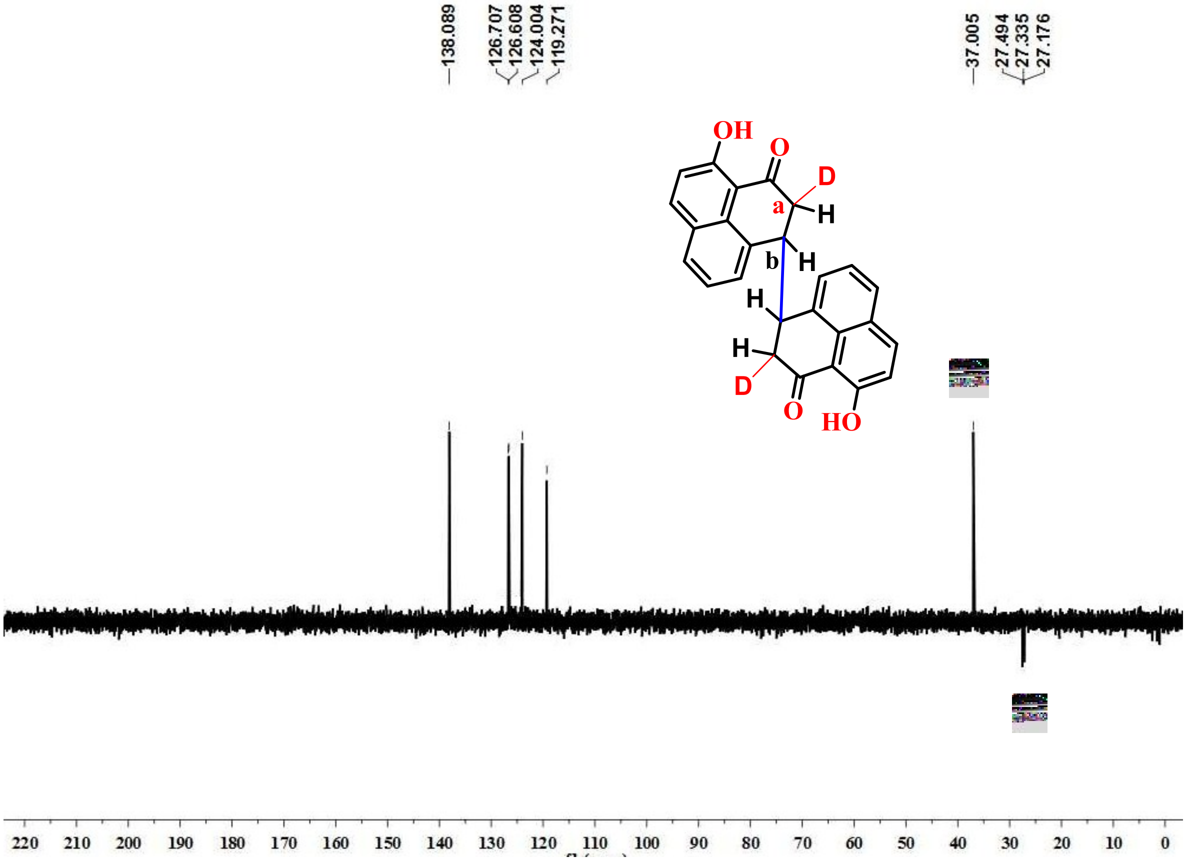

Figure S62. DEPT-135 NMR spectrum of PLY dimer-D_2, $\mathbf{5_D}$ recorded in $CDCl_3$.

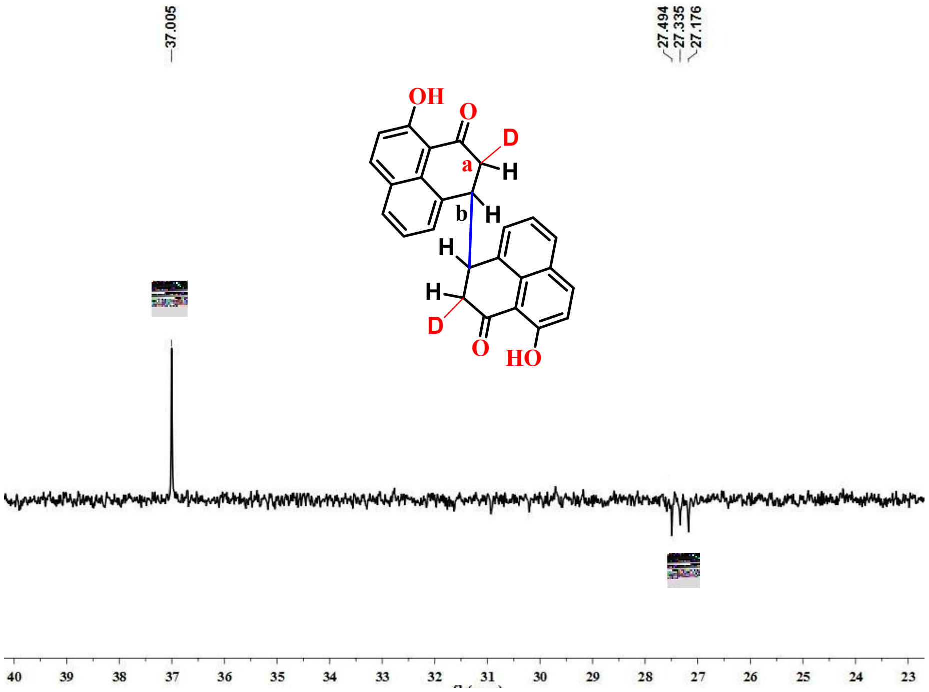

Figure S63. Zoomed version of DEPT-135 NMR spectrum from 40–23 ppm of PLY dimer-D_2, $\mathbf{5_D}$ recorded in $CDCl_3$.

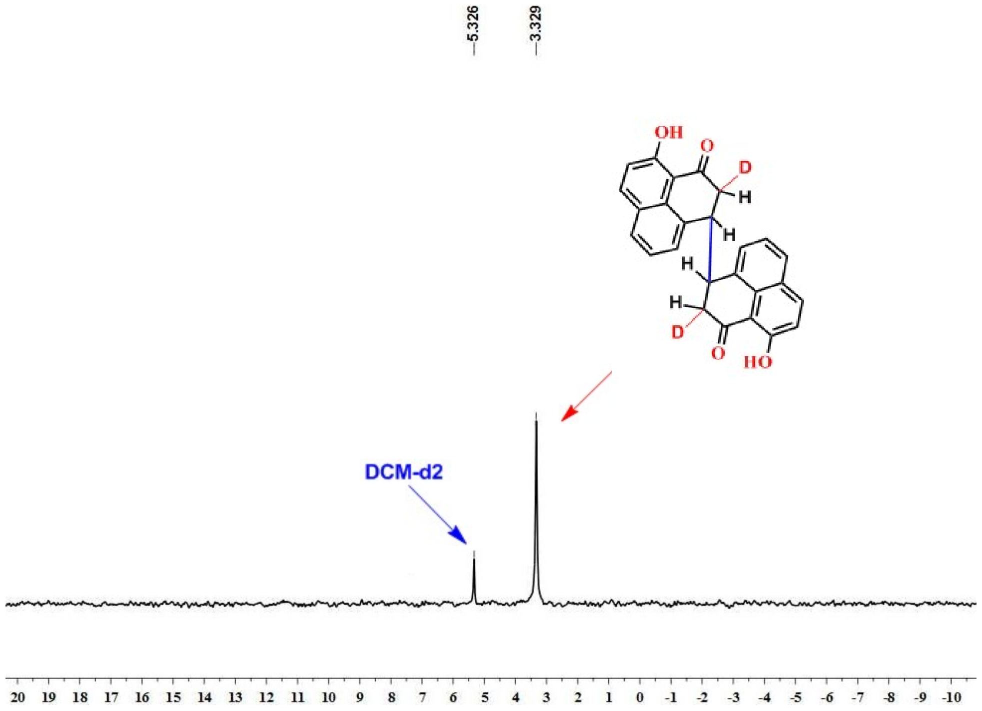

Figure S64. $^2H\{^1H\}$ NMR spectrum of PLY dimer-D_2, $\mathbf{5_D}$ recorded in dichloromethane.

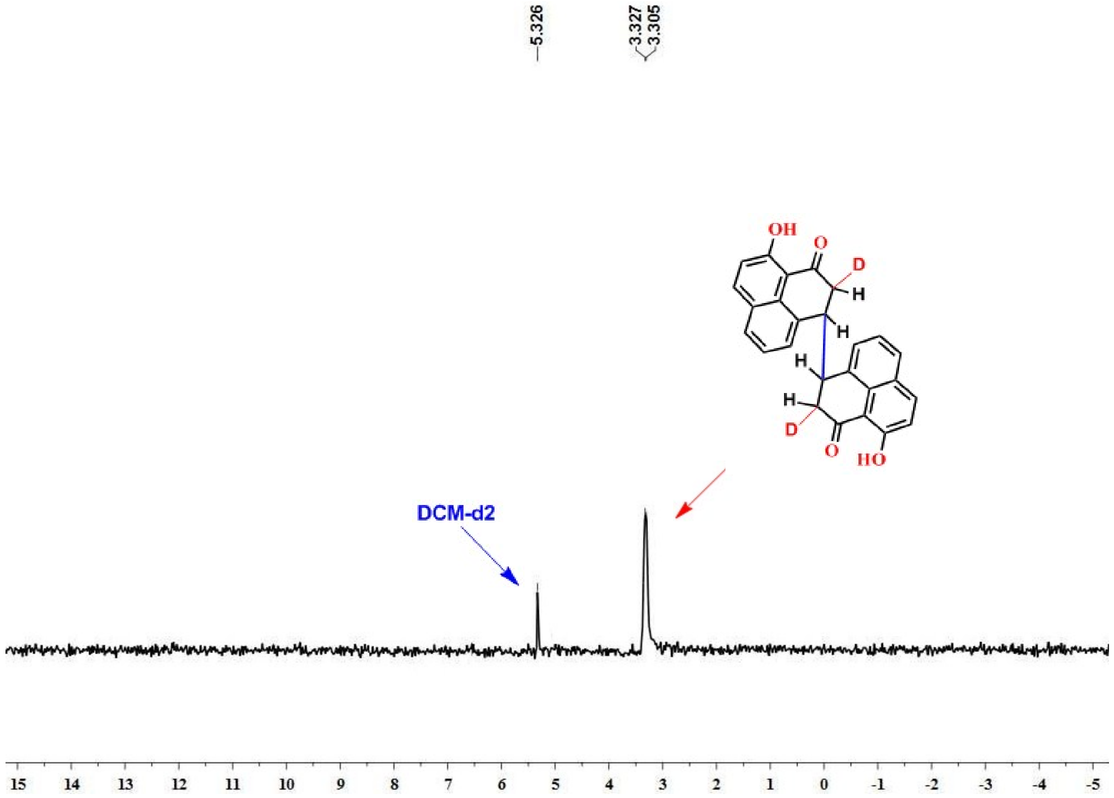

Figure S65. ^2H NMR spectrum of PLY dimer-D_2, $\mathbf{5_D}$ recorded in dichloromethane.

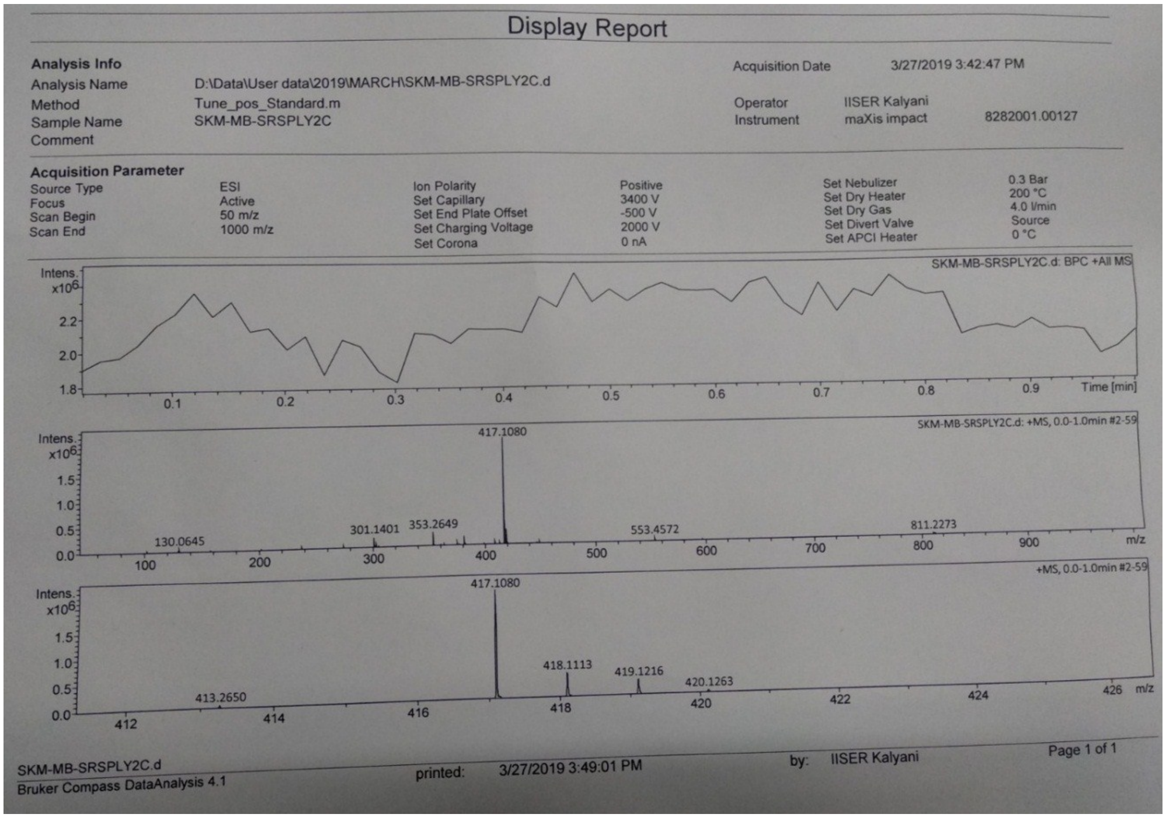

Figure S66. HRMS spectrometry of PLY Dimer-D_2, $\mathbf{5_D}$.

12e. NMR characterization of byproduct silanol.

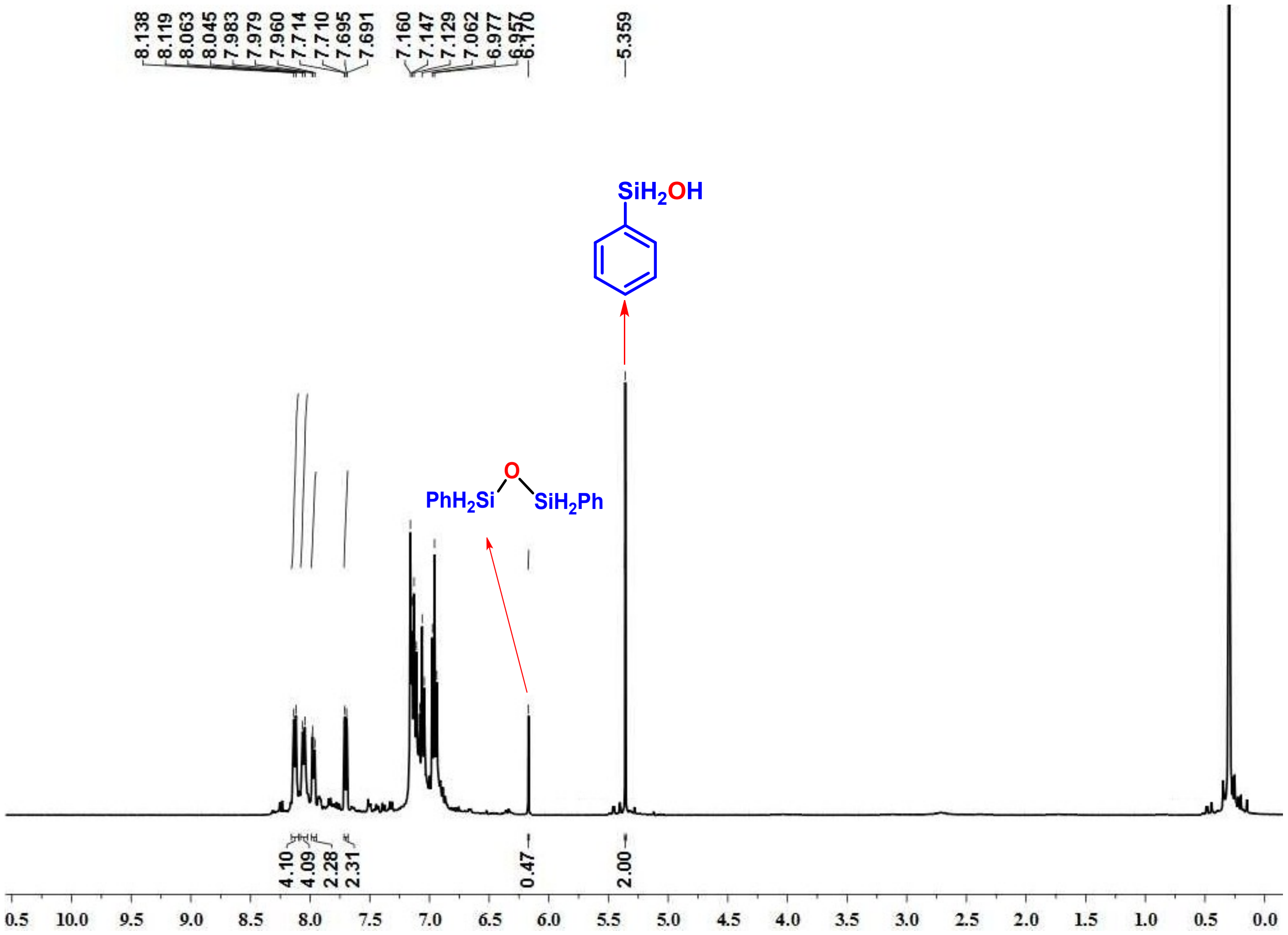

Figure S67. ^{1}H NMR spectrum of reaction mixture revealing phenylsilanol and silylether recorded in C_6D_6.

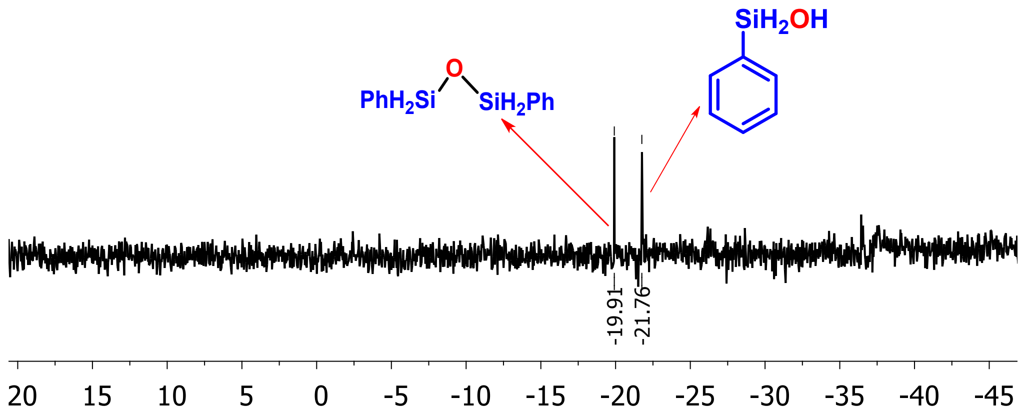

Figure S68. ^{29}Si NMR spectrum of reaction mixture reveals phenylsilanol and silylether recorded in C_6D_6.

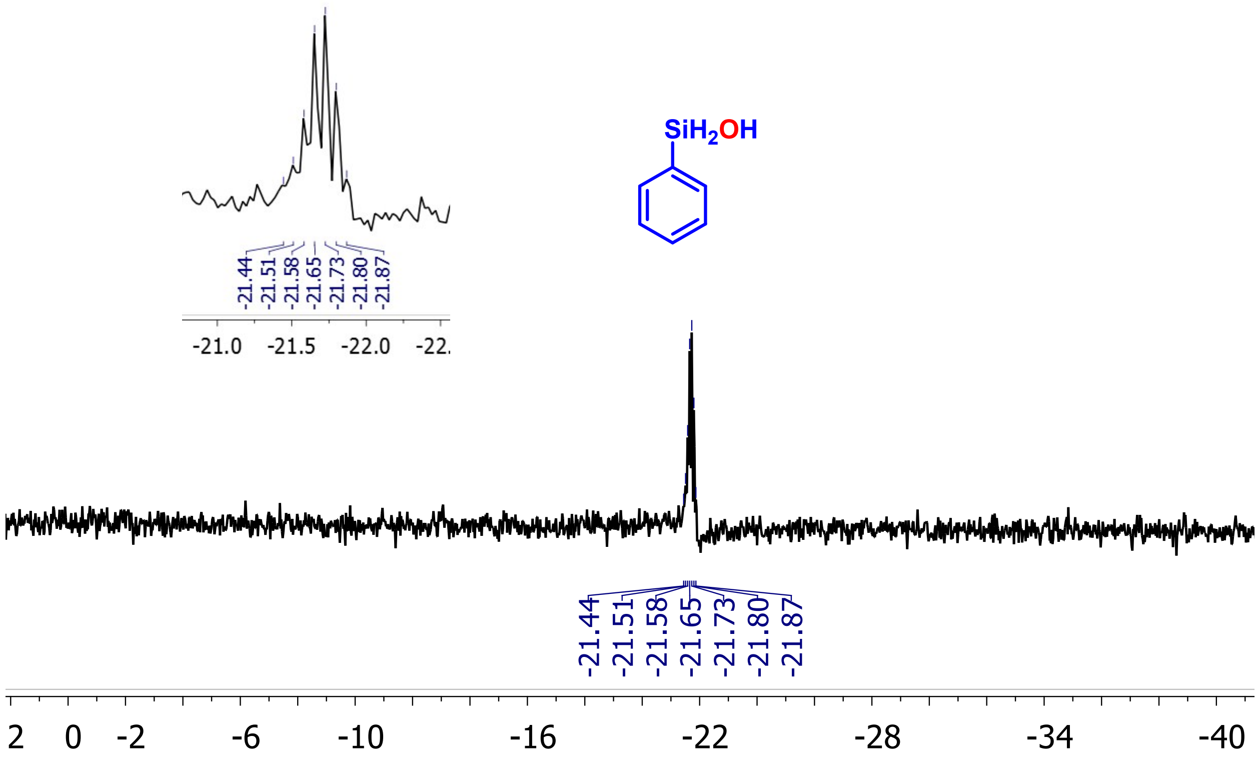

Figure S69. ^{29}Si NMR spectrum of phenylsilanol recorded in C_6D_6.

12f. NMR characterization of hydrosilylated product (9) of benzoic acid.

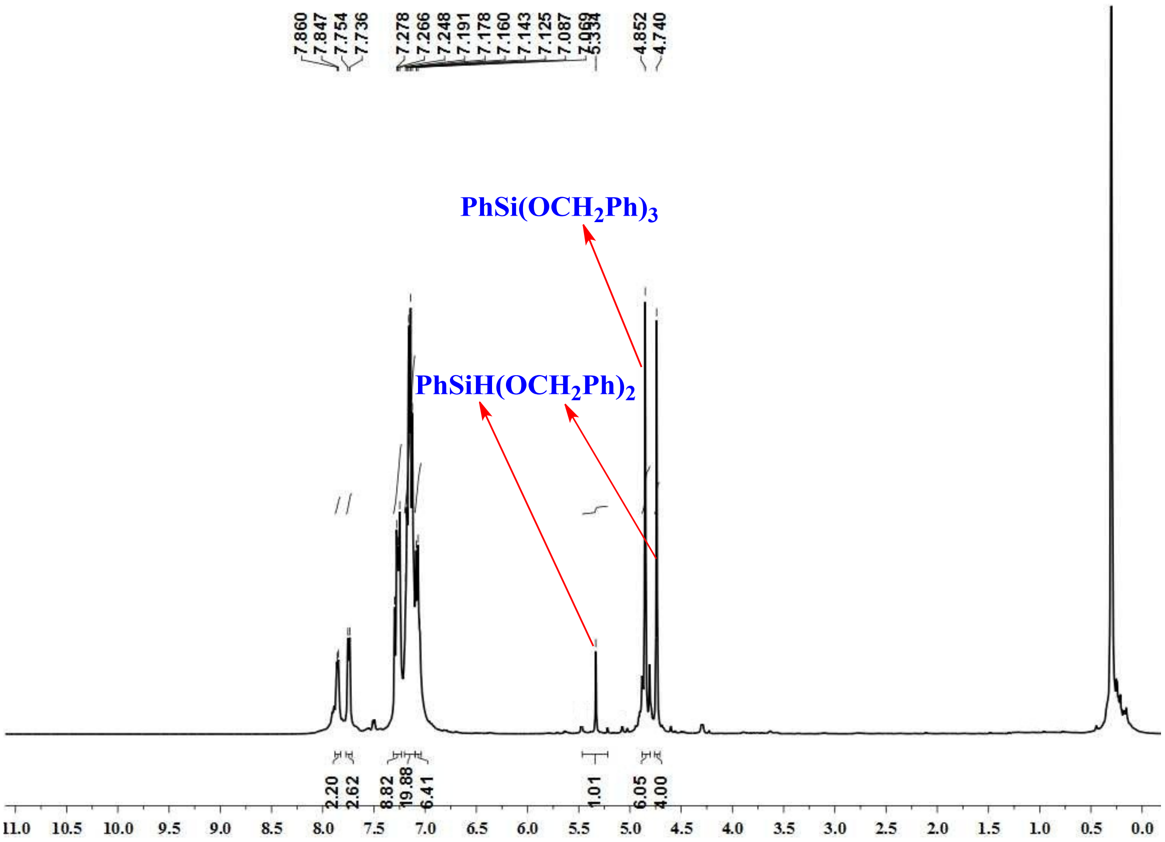

Figure S70. ^{1}H NMR spectrum of hydrosilylated product, **9** recorded in C_6D_6 generating from benzoic acid.

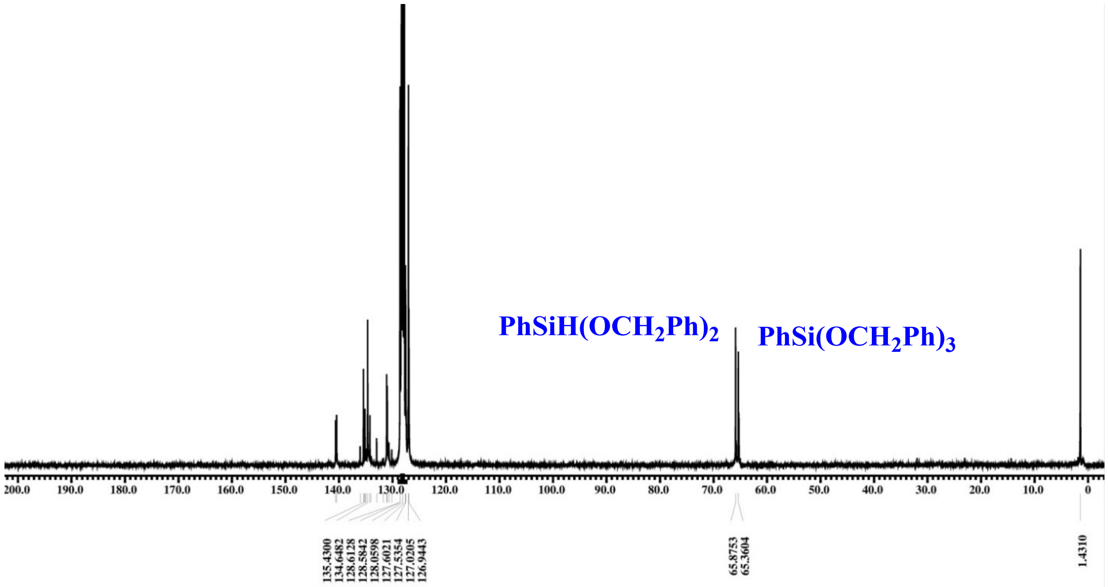

Figure S71. ^{13}C NMR spectrum of hydrosilylated product, **9** recorded in C_6D_6.

12g. Characterization of deuterium scrambling experiment

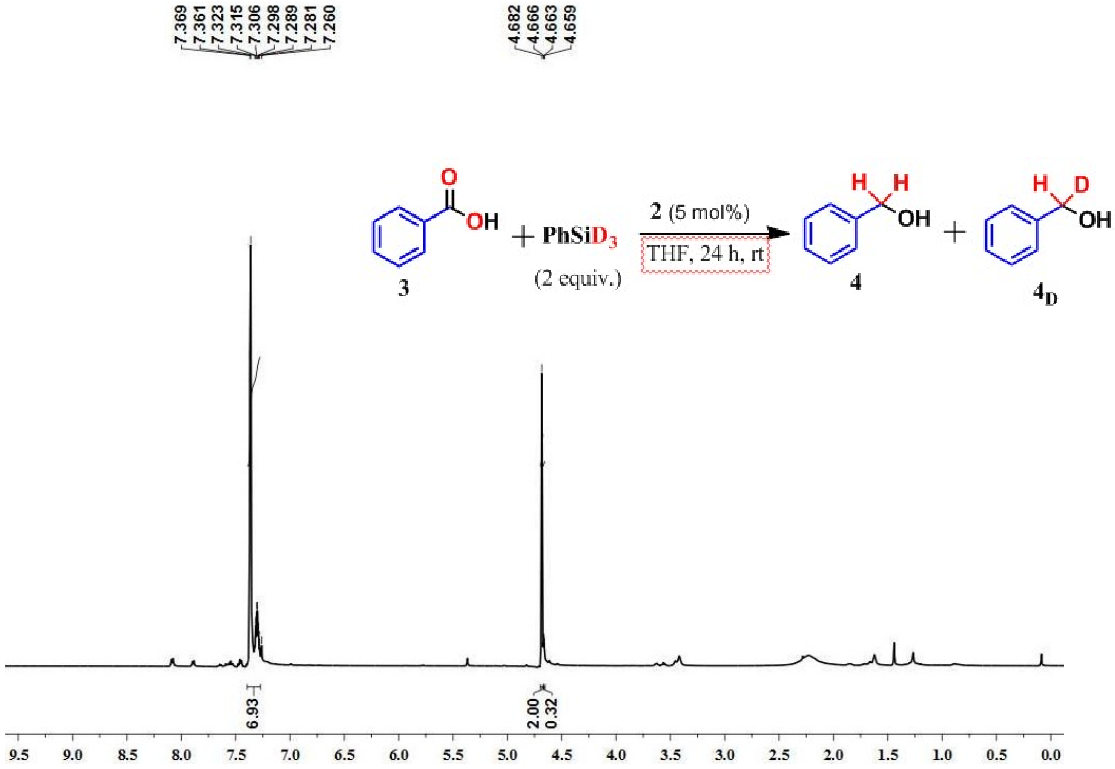

Figure S72. ^{1}H NMR spectrum for mixture of $PhCH_2OH$ (**4**) and PhCHDOH (**4_D**) recorded in $CDCl_3$, when the catalytic reaction was performed in THF.

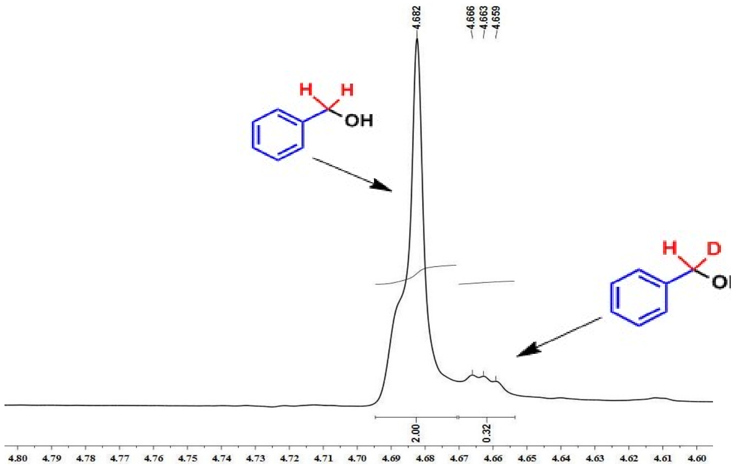

Figure S73. Zoomed version of ^{1}H NMR spectrum (4.80 – 4.60) ppm for mixture of $PhCH_2OH$ (**4**) and PhCHDOH (**4_D**) recorded in $CDCl_3$, when the catalytic reaction was performed in THF.

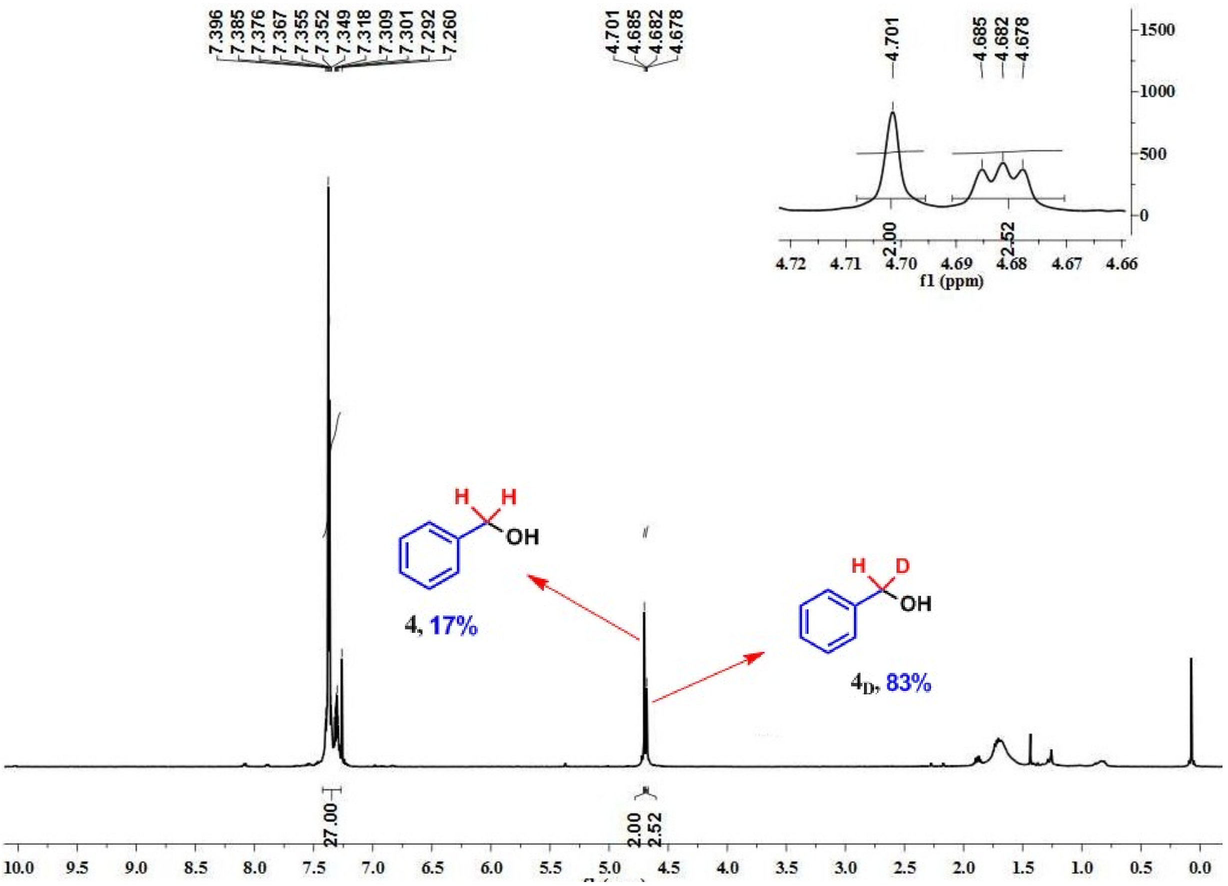

Figure S74. ^{1}H NMR spectrum for mixture of $PhCH_2OH$ (**4**) and PhCHDOH (**4_D**) recorded in $CDCl_3$, when the catalytic reaction was performed in THF-d_8.

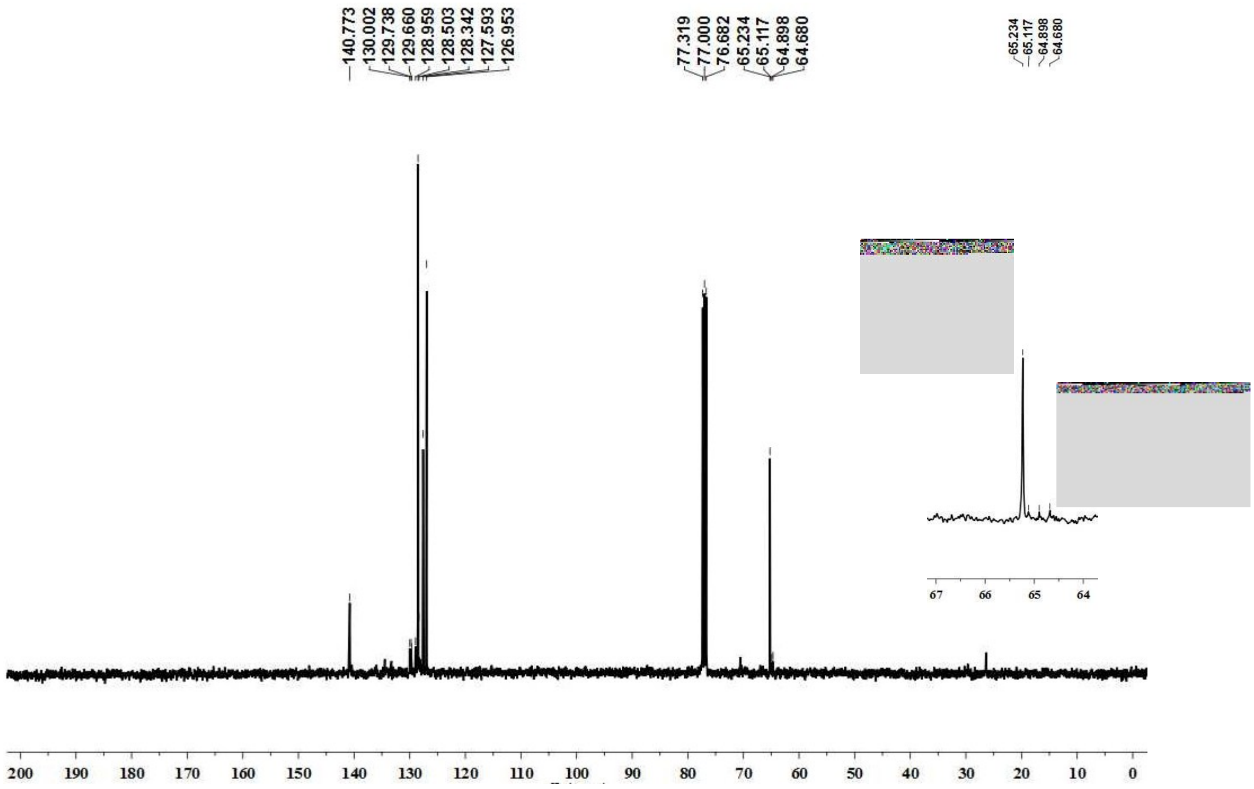

Figure S75. ^{13}C NMR spectrum for mixture of $PhCH_2OH$ (**4**) and PhCHDOH (**4_D**) recorded in $CDCl_3$ and zoomed version of ^{13}C (67– 64 ppm) is shown in inset.

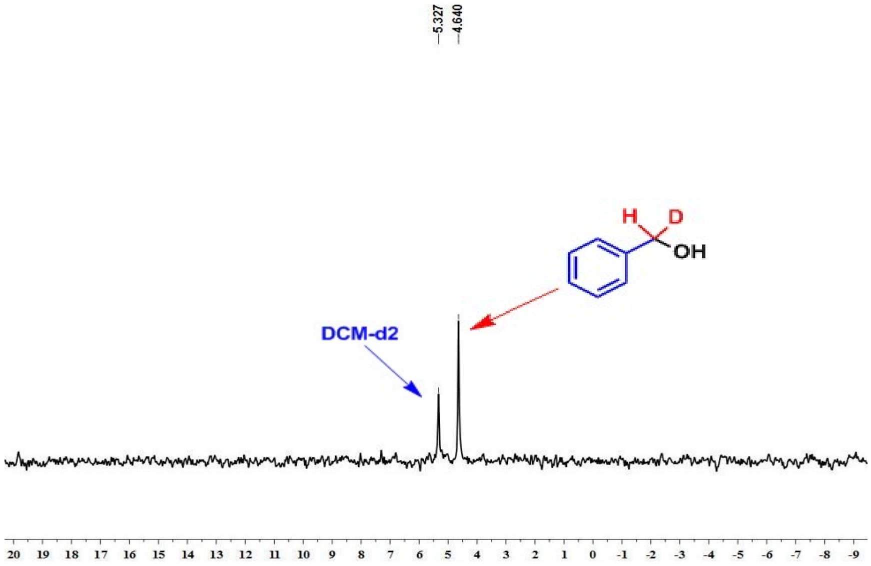

Figure S76. ^2H NMR spectrum for mixture of $PhCH_2OH$ (**4**) and PhCHDOH (**4_D**) recorded in Dichloromethane.

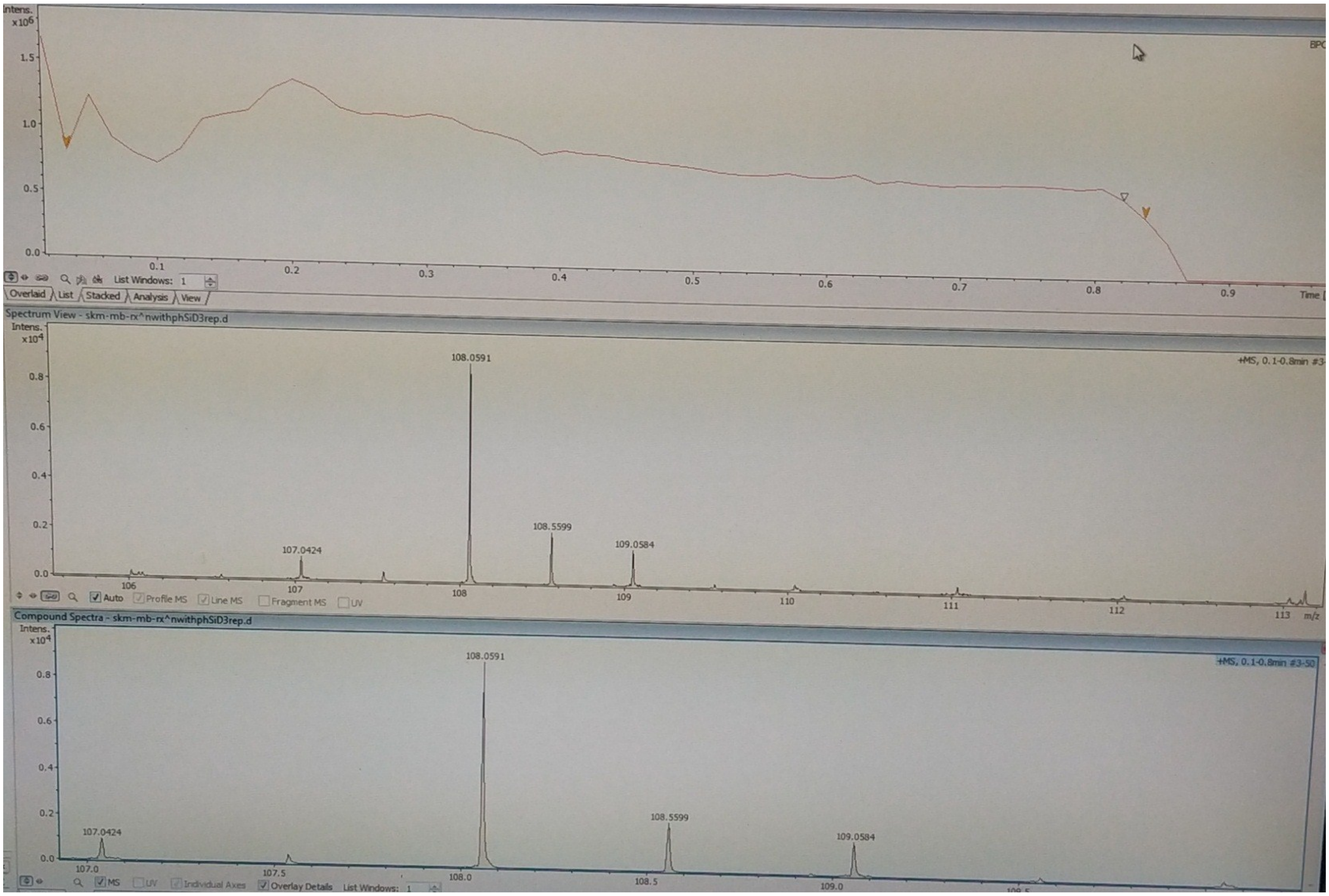

Figure S77. HRMS spectrometry for mixture of $PhCH_2OH$ (**4**) and PhCHDOH (**4_D**).

13. Computational Details

All calculations were carried out using Density Functional Theory as implemented in the Gaussian 09[S27] quantum chemistry programs. The geometries of stationary points were optimized with the generalized gradient approximation (GGA) by means of the Becke exchange functional along with Lee, Yang, Parr correlation functional (LYP). We used double-ζ basis set with the relativistic effective core potential of Hay and Wadt (LANL2DZ) for the cobalt atom and 6-31+G(d) basis set for other elements (H, C, O). The geometries were optimized without any symmetry constraints. For the optimization, full model was chosen with furan as the weakly coordinating ligand. The symmetry broken DFT solution was detected using the Gaussian keyword Stable=opt. Harmonic force constants were computed at the optimized geometries to characterize the stationary points as minima. The molecular orbitals were visualized and spin density was plotted using Gaussview.

Coordinates for calculated structures:

1

C	3.48767100	-0.00014400	-2.47992300
C	2.69793600	-0.00006700	-1.26433100
C	3.39990700	0.00000300	0.00003300
C	4.83504000	-0.00000700	0.00002800
C	5.57220800	-0.00008500	-1.22387600
C	4.84606200	-0.00015300	-2.45963500
C	2.69794400	0.00008300	1.26440300
C	5.57221700	0.00006100	1.22392600
C	4.84607900	0.00014000	2.45969000
C	3.48768800	0.00015100	2.47998900
C	6.97622400	0.00004900	1.20213400
C	7.68003400	-0.00002800	0.00001800
C	6.97621600	-0.00009500	-1.20209300
H	7.51323600	-0.00015500	-2.14799100
H	8.76596200	-0.00003600	0.00001400
H	2.92811000	-0.00019600	-3.41034100
H	5.40935800	-0.00021300	-3.39073200
H	2.92813400	0.00021100	3.41041000

C	-6.97622500	0.00006700	1.20213500
C	-5.57221700	0.00007700	1.22392800
C	-4.84607900	0.00017700	2.45969200
C	-7.68003400	-0.00003000	0.00001900
C	-4.83504000	-0.00001300	0.00002900
C	-5.57220800	-0.00011100	-1.22387400
C	-6.97621600	-0.00011800	-1.20209200
C	-4.84606200	-0.00020200	-2.45963300
H	-5.40935800	-0.00027700	-3.39073100
C	-3.48767100	-0.00019500	-2.47992200
C	-2.69793600	-0.00009600	-1.26433000
C	-3.39990700	-0.00000300	0.00003500
C	-2.69794400	0.00009800	1.26440400
C	-3.48768900	0.00018700	2.47999000
H	-2.92813500	0.00026100	3.41041200
H	-8.76596300	-0.00003600	0.00001600
H	-7.51323600	-0.00019500	-2.14798900
H	-2.92811100	-0.00026200	-3.41033900
H	5.40938200	0.00019300	3.39078400
H	7.51325100	0.00010200	2.14802800
H	-5.40938200	0.00024500	3.39078500
H	-7.51325100	0.00013700	2.14802900
O	-1.42731600	-0.00009700	-1.40700800
O	-1.42732600	0.00011700	1.40709200
O	1.42731600	-0.00006600	-1.40701000
O	1.42732700	0.00009900	1.40709000
C	-0.00015000	3.16756700	1.10660500
C	-0.00010500	4.46900700	0.71911600
H	-0.00027500	2.64953800	2.05213300
C	0.00008900	4.46878100	-0.72009100
H	-0.00019400	5.33065300	1.37196200
H	0.00017800	5.33022300	-1.37320600

C	-0.00004100	-3.16735400	-1.10675500
C	-0.00001800	-4.46885900	-0.71948500
H	-0.00008600	-2.64916200	-2.05219400
C	0.00004000	-4.46887600	0.71972200
H	-0.00004300	-5.33039600	-1.37247500
H	0.00006800	-5.33042800	1.37269200
C	0.00013400	3.16722000	-1.10717200
C	0.00005700	-3.16738000	1.10702200
O	-0.00000700	2.35808000	-0.00015600
O	0.00000600	-2.35805400	0.00014300
H	0.00025900	2.64889100	-2.05253600
H	0.00010000	-2.64921400	2.05247500
Co	0.00000000	0.00001400	0.00005000

2 (sextet)

C	3.54157000	0.00174600	-2.45249100
C	2.75419000	0.00111300	-1.25569400
C	3.46015000	-0.00044600	0.02358800
C	4.90839700	-0.00130900	0.03597500
C	5.66290400	-0.00065600	-1.20508000
C	4.93630400	0.00089300	-2.43202500
C	2.72998900	-0.00106600	1.28953300
C	5.64025800	-0.00283400	1.28985700
C	4.89106900	-0.00340100	2.50362900
C	3.49789900	-0.00253900	2.49992900
C	7.07345300	-0.00368900	1.27340700
C	7.78117300	-0.00305400	0.06198200
C	7.09569000	-0.00155400	-1.16254000
H	7.64805300	-0.00103900	-2.11085800
H	8.87985100	-0.00372400	0.07197900
H	2.98896400	0.00288200	-3.39856300
H	5.49989600	0.00139400	-3.37515300

H	2.92866600	-0.00297200	3.43575300
C	-7.09449400	-0.00059800	1.16123400
C	-5.66171200	0.00021400	1.20385500
C	-4.93518800	0.00152400	2.43084500
C	-7.77990900	-0.00185400	-0.06332700
C	-4.90713400	-0.00028100	-0.03715800
C	-5.63892500	-0.00155100	-1.29107700
C	-7.07211800	-0.00232600	-1.27470900
C	-4.88966800	-0.00195700	-2.50481200
H	-5.43680100	-0.00289600	-3.45761400
C	-3.49650700	-0.00119600	-2.50103700
C	-2.72864500	-0.00000900	-1.29059800
C	-3.45888500	0.00047000	-0.02469400
C	-2.75300500	0.00183900	1.25463200
C	-3.54044900	0.00231800	2.45138900
H	-2.98789500	0.00330000	3.39749100
H	-8.87858500	-0.00246200	-0.07338700
H	-7.60698300	-0.00329200	-2.23296400
H	-2.92721900	-0.00151500	-3.43682700
H	5.43824900	-0.00454000	3.45640500
H	7.60837400	-0.00484600	2.23163000
H	-5.49883400	0.00189500	3.37394000
H	-7.64690900	-0.00020900	2.10952100
O	-1.41592900	0.00059500	-1.39424200
O	-1.43811400	0.00266600	1.38077900
O	1.43931700	0.00197200	-1.38176900
O	1.41726600	-0.00033200	1.39322900
C	-0.90818800	3.69148500	0.63849100
C	-0.59346000	5.01126200	0.41726400
H	-1.68766700	3.16234400	1.17866300
C	0.59078900	5.01297300	-0.41203400
H	-1.13620100	5.87697700	0.79500600

H	1.13190500	5.88024900	-0.78852900
C	0.90864800	-3.69291400	-0.63018000
C	0.58934600	-5.01156000	-0.40876800
H	1.69266000	-3.16668700	-1.16662600
C	-0.59987300	-5.00901100	0.41337300
H	1.13206400	-5.87921000	-0.78208300
H	-1.14554200	-5.87433500	0.78777700
C	0.90797800	3.69410300	-0.63515000
C	-0.91470000	-3.68901400	0.63313200
O	0.00064400	2.88053800	0.00106500
O	-0.00166400	-2.87868400	0.00092600
H	1.68847100	3.16724200	-1.17608500
H	-1.69692400	-3.15942100	1.16887700
Co	0.00068800	0.00126300	-0.00047100

2 (doublet)

C	-3.40837000	-0.00073500	2.42738600
C	-2.63059000	0.00138300	1.23751800
C	-3.33223300	-0.00006800	-0.02534400
C	-4.76967400	-0.00309000	-0.03918400
C	-5.51822800	-0.00489600	1.19190100
C	-4.79309800	-0.00366800	2.40971100
C	-2.60170400	0.00151700	-1.27409000
C	-5.49276100	-0.00436500	-1.28437900
C	-4.74165300	-0.00262900	-2.48778100
C	-3.35962200	0.00010500	-2.47935000
C	-6.91525600	-0.00730500	-1.27113400
C	-7.61807900	-0.00900700	-0.06843100
C	-6.94076100	-0.00785000	1.14878300
H	-7.49116200	-0.00920200	2.08939100
H	-8.70918100	-0.01127800	-0.07986500
H	-2.85798700	0.00025400	3.36623300

H	-5.34944700	-0.00509700	3.34795800
H	-2.79110800	0.00131200	-3.40681000
C	6.94078800	-0.00710500	-1.14821200
C	5.51825300	-0.00410200	-1.19135200
C	4.79314200	-0.00253200	-2.40916700
C	7.61808500	-0.00856800	0.06901300
C	4.76968300	-0.00258300	0.03972700
C	5.49275100	-0.00415100	1.28493300
C	6.91525000	-0.00712600	1.27170700
C	4.74162600	-0.00264200	2.48832100
H	5.28001000	-0.00379500	3.43708100
C	3.35959100	0.00012600	2.47986900
C	2.60169300	0.00178300	1.27460500
C	3.33223900	0.00046500	0.02586900
C	2.63062500	0.00229300	-1.23700100
C	3.40840500	0.00046900	-2.42685200
H	2.85803700	0.00173400	-3.36570900
H	8.70918800	-0.01087100	0.08046200
H	7.44589700	-0.00829200	2.22355900
H	2.79106400	0.00115000	3.40732200
H	-5.28005300	-0.00356900	-3.43653200
H	-7.44591700	-0.00824300	-2.22297900
H	5.34949800	-0.00374100	-3.34740900
H	7.49120400	-0.00824000	-2.08881200
O	1.30411900	0.00431300	1.37635600
O	1.32798700	0.00561300	-1.36383400
O	-1.32798100	0.00458800	1.36433400
O	-1.30414100	0.00406400	-1.37585300
C	0.74745000	3.33035500	-0.80637400
C	0.48909000	4.63915400	-0.52781200
H	1.39105200	2.80312700	-1.49324700
C	-0.49058000	4.63973900	0.52578200

H	0.93895500	5.49814600	-1.00805200
H	-0.94028000	5.49926100	1.00522900
C	-0.75314100	-3.32480000	0.79982300
C	-0.49225000	-4.63320200	0.52178000
H	-1.40191200	-2.79888500	1.48284000
C	0.49471300	-4.63227900	-0.52496200
H	-0.94505100	-5.49285400	0.99805900
H	0.94822900	-5.49108900	-1.00207700
C	-0.74919900	3.33124600	0.80553300
C	0.75452400	-3.32339200	-0.80173300
O	-0.00095400	2.53297700	-0.00004700
O	0.00036200	-2.52620900	-0.00055800
H	-1.39297700	2.80483000	1.49286500
H	1.40284500	-2.79626200	-1.48424000
Co	-0.00001100	0.00457800	0.00025600

14. **References**.

S1. S. Elangovan, M. Garbe, H. Jiao, A. Spannenberg, K. Junge and M. Beller, *Angew.Chem.,Int. Ed.,* 2016, **55**, 15364 –15368.

S2. C. P. Prasanth, E. Joseph, A. Abhijith, D. S. Nair, I. Ibnusaud, J. Raskatov and B. Singaram, *J. Org. Chem.,* 2018, **83**, 1431−1440.

S3. Z. Wang, X. Chen, B. Liu, Q.-b. Liu, G. A. Solan, X. Yang and W.-H. Sun, *Catal. Sci. Technol.,* 2017, **7**, 1297–1304.

S4. R. Wang, Y. Tang, M. Xu, C. Meng and F. Li, *J. Org. Chem.,* 2018, **83**, 2274−2281.

S5. L. C. M. Castro, D. Bèzier, J.-B. Sortais and C. Darcel, *Adv. Synth. Catal.,* 2011, **353**, 1279–1284.

S6. H. Yang, Y. Li, M. Jiang, J. Wang and H. Fu, *Chem. Eur. J.,* 2011, **17**, 5652–5660.

S7. D. A. Everson, R. Shrestha and D. J. Weix, *J. Am. Chem. Soc.,* 2010, **132**, 920–921.

S8. N. Murai, M. Yonaga and K. Tanaka, *Org. Lett.,* 2012, **14**, 1278–1281.

S9. S. Baldino, S. Facchetti, H. G. Nedden, A. Zanotti-Gerosa and W. Baratta, *ChemCatChem,* 2016, **8**, 3195-3198.

S10. H. Seo, A. Liu and T. F. Jamison, *J. Am. Chem. Soc.,* 2017, **139**, 13969−13972.

S11. S. R. Tamang and M. Findlater, *J. Org. Chem.,* 2017, **82**, 12857−12862.

S12. M. B. Widegren, G. J. Harkness, A. M. Z. Slawin, D. B. Cordes and M. L. Clarke, *Angew. Chem., Int. Ed.,* 2017, **56**, 5825–5828.

S13. C. Doebelin, R. Patouret, R. D. Garcia-Ordonez, M. R. Chang, V. Dharmarajan, D. S. Kuruvilla, S. J. Novick, L. Lin, M. D. Cameron, P. R. Griffin and T. M. Kamenecka, *ChemMedChem,* 2016, **11**, 2607–2620.

S14. Z. Yang, Z. Zhu, R. Luo, X. Qiu, J. Liu, J.-K. Yang and W. Tang, *Green Chem.,* 2017, **19**, 3296–3301.

S15. Y. Zou, J. E. Melvin, S. S. Gonzales, M. J. Spafford and A. B. Smith, III, *J. Am. Chem. Soc.,* 2015, **137**, 7095−7098.

S16. A. L. Gill, M. Frederickson, A. Cleasby, S. J. Woodhead, M. G. Carr, A. J. Woodhead, M. T. Walker, M. S. Congreve, L. A. Devine, D. Tisi, M. O'Reilly, L. C. A. Seavers, D. J. Davis, J. Curry, R. Anthony, A. Padova, C. W. Murray, R. A. E. Carr and H. Jhoti, *J. Med. Chem.,* 2005, **48**, 414−426.

S17. D. P. Hari, P. Schroll and B. König, *J. Am. Chem. Soc.,* 2012, **134**, 2958−2961.

S18. G. Vijaykumar, A. Pariyar, J. Ahmed, B. K. Shaw, D. Adhikari and S. K. Mandal, *Chem. Sci.,* 2018, **9**, 2817-2825.

S19. B. Wang and Z. Cao, *RSC Adv.,* 2013, **3**, 14007–14015.

S20. J. Moineau, M. Granier and G. F. Lanneau, *Langmuir,* 2004, **20**, 3202-3207.

S21. T. K. Mukhopadhyay, C. L. Rock, M. Hong, D. C. Ashley, T. L. Groy, M.-H. Baik and R. J. Trovitch, *J. Am. Chem. Soc.,* 2017, **139**, 4901−4915.

S22. O. V. Dolomanov, L. J. Bourhis, R. J. Gildea, J. A. K. Howard and H. Puschmann, *J. Appl. Cryst.,* 2009, **42**, 339-341.

S23. L. Palatinus and G. Chapuis, *J. Appl. Cryst.,* 2007, **40**, 786-790.

S24. G. M. Sheldrick, *Acta Cryst.,* 2008, **A64**, 112-122.

S25. L. J. Barbour, *X-Seed, Graphical Interface to SHELX97 and POV-Ray*; University of Missouri-Columbia: Columbia, MO, 1999.

S26. R. C. Haddon, R. Rayford and A. M. Hirani, *J. Org. Chem.,* 1981, **46**, 4587-4588.

S27. M. J. Frisch, G. W. Trucks, H. B. Schlegel, G. E. Scuseria, M. A. Robb, J. R. Cheeseman, G. Scalmani, V. Barone, B. Mennucci, G. A. Petersson, H. Nakatsuji, M. Caricato, X. Li, H. P. Hratchian, A. F. Izmaylov, J. Bloino, G. Zheng, J. L. Sonnenberg, M. Hada, M. Ehara, K. Toyota, R. Fukuda, J. Hasegawa, M. Ishida, T. Nakajima, Y. Honda, O. Kitao, H. Nakai, T. Vreven, J. A. Montgomery, Jr., J. E. Peralta, F. Ogliaro, M. Bearpark, J. J. Heyd, E. Brothers, K. N. Kudin, V. N. Staroverov, R. Kobayashi, J. Normand, K. Raghavachari, A. Rendell, J. C. Burant, S. S. Iyengar, J. Tomasi, M. Cossi, N. Rega, J. M. Millam, M. Klene, J. E. Knox, J. B. Cross, V. Bakken, C. Adamo, J. Jaramillo, R. Gomperts, R. E. Stratmann, O. Yazyev, A. J. Austin, R. Cammi, C. Pomelli, J. W. Ochterski, R. L. Martin, K. Morokuma, V. G. Zakrzewski, G. A. Voth, P. Salvador, J. J. Dannenberg, S. Dapprich, A. D. Daniels, Ö. Farkas, J. B. Foresman, J. V. Ortiz, J. Cioslowski and D. J. Fox, Gaussian 09, Gaussian Inc.,Wallingford, CT, 2010.
